# Reciprocal associations between confidence in getting social support and academic expectancies and subjective task values: Stronger for first‐generation and transfer students

**DOI:** 10.1111/bjep.12751

**Published:** 2025-02-26

**Authors:** Hanna Gaspard, Cora Parrisius, Luise von Keyserlingk, Charlott Rubach, Katsumi Yamaguchi‐Pedroza, Hye Rin Lee, Marion Spengler, Christian Fischer, Jutta Heckhausen, Jacquelynne S. Eccles

**Affiliations:** ^1^ Center for Research on Education and School Development TU Dortmund University Dortmund Germany; ^2^ Hector Research Institute of Education Sciences and Psychology University of Tübingen Tübingen Germany; ^3^ Karlsruhe University of Education Karlsruhe Germany; ^4^ University of Rostock Rostock Germany; ^5^ Department of Psychology The Pennsylvania State University University Park Pennsylvania USA; ^6^ Owens Institute for Behavioral Research University of Georgia Athens Georgia USA; ^7^ Medical School Berlin Berlin Germany; ^8^ Department of Psychological Science University of California Irvine California USA; ^9^ School of Education University of California Irvine California USA

**Keywords:** first‐generation students, situated expectancy–value theory, social support, transfer students

## Abstract

**Background:**

Social support is assumed to play a key role in motivation at university, particularly for disadvantaged students, such as first‐generation and community college transfer students. However, longitudinal research investigating reciprocal associations between social support and motivation is lacking.

**Aims:**

We examined such associations between confidence in getting support from faculty and peers and students' expectancies and subjective task values in their most difficult and most important course.

**Sample:**

Data stemmed from two cohorts of undergraduate students (*n* = 320/417 in Fall 2019/2020) at a diverse Southern Californian university.

**Methods:**

Students reported on their confidence in getting support and their expectancies and subjective task values at the beginning, in the middle and (only for motivation) at the end of the academic term.

**Results:**

Results indicated no differences in confidence in getting support based on university generation or transfer student status. Cross‐lagged panel models provided some evidence for reciprocal associations between students' confidence in getting support and their expectancies and subjective task values. Findings were similar across the Fall 2019 and Fall 2020 cohorts, providing support for the generalizability across in‐person vs. remote learning settings. Longitudinal associations tended to be stronger for first‐generation and transfer students.

**Conclusions:**

Future research should, therefore, examine whether university programmes targeting social support are especially effective for disadvantaged students.

## INTRODUCTION

Entering university is an important, yet often challenging transition, especially for students who traditionally experience disadvantages at university (Saenz et al., 2007). Students' experiences in this new context can affect their academic motivation, which can be an important antecedent of later achievement and dropout (e.g. Benden & Lauermann, 2022; Robinson et al., 2019).

Several theoretical approaches, including stage–environment fit theories and models of university retention, postulate that social support and students' academic motivation are interrelated and drive a successful transition into new environments such as university (Eccles & Roeser, 2009; Tinto, 1993). Although prior research has generally supported this assumption (e.g. Credé & Niehorster, 2012; Gillen‐O'Neel, 2021; Hausmann et al., 2007), longitudinal research examining the reciprocal associations between the various dimensions of social support and academic motivation amongst university students is lacking. In this study, we investigate such longitudinal associations, differentiating between confidence in getting support by peers and faculty, who both constitute important sources of social support in university. Furthermore, we draw on situated expectancy‐value theory (SEVT; Eccles & Wigfield, 2020) to conceptualize academic motivation in terms of students' expectancies of success in different university courses, the positive values attached to these courses and the perceived cost of engaging in them.

Social support may be a particularly important resource for groups of students who are disadvantaged at university. In the United States, these groups of students include first‐generation (FG) and transfer students (i.e. students who transfer to four‐year universities from community colleges). These students often face the following challenges: reduced academic preparation for university education, lower family financial resources, limited family familiarity with the university system and higher personal doubts about belonging at university (Chin‐Newman & Shaw, 2013; Gillen‐O'Neel, 2021; Jenkins et al., 2013; Pascarella et al., 2004; Saenz et al., 2007). Thus, we compared the associations between confidence in getting support and academic motivation for different subgroups of students.

### Confidence in getting social support during one's university years

According to the stage–environment fit approach, educational adaptation is facilitated by the fit between students' needs for support and the expected support available through the learning environment (Eccles & Roeser, 2009). Tinto (1993) posited the importance of both the academic and social systems for a successful university experience and suggested that students' perceptions of the availability of needed social support from both faculty and peers are critical for academic success and social integration. Faculty and peers both constitute important socialization figures in university who can support students study‐related matters (e.g. providing help in the academic context) or with more personal matters (e.g. availability to talk about personal matters). In support of these suggestions, several scholars (e.g. Meeuwisse et al., 2010; Severiens & Wolff, 2008) have shown that the availability of faculty and peer social support is associated with students' adjustment to university, with support from faculty being most strongly related to academic adjustment and support from peers being particularly important for social adjustment (Credé & Niehorster, 2012).

In the following, we review research on both social support and social belonging, a construct closely related to social support (e.g. Hausmann et al., 2007; Meeuwisse et al., 2010). Sense of belonging is typically defined as a sense of affiliation and fit with the university community and can thus be conceptually differentiated from more concrete forms of social support. In line with such a definition, it is often measured with short scales assessing an overall sense of belonging (e.g. “I feel as if I really belong at this school”; Hausmann et al., 2007; Murphy & Zirkel, 2015; Ostrove & Long, 2007). However, other studies assess sense of belonging with scales tapping different forms of perceived social support (e.g. Hoffman et al., 2002; Pittman & Richmond, 2008) so that the differentiation between these two constructs becomes even more difficult. In our study, we more specifically assessed students' confidence in receiving social support from peers and faculty. Students finding themselves in an environment where they feel comfortable in asking for help and confident they can get support if needed is a central contributing factor to experiencing a sense of belonging (e.g. Hoffman et al., 2002). We believe this measure is more meaningful than asking for the amount of support received in a fixed past set of time because some students likely need more support than others and thus variations in amount of support received are confounded with the amount of support needed.

Notably, previous research has found that students report high mean levels of perceived support by faculty and—in particular—peers and that there is little average change in perceived support over the first college semesters (Pan & Gauvain, 2012; Pittman & Richmond, 2008). Although this can limit the possibility of finding strong longitudinal associations with outcomes, there is also interindividual variation in change over time (Pittman & Richmond, 2008). Moreover, whereas perceived social support was typically high in “regular” in‐person learning settings, social support was considerably reduced under remote learning settings during the COVID‐19 pandemic (Von Keyserlingk et al., 2024). It is thus important to investigate links between perceived social support and academic outcomes under various learning conditions.

### Associations between social support and academic motivation

Various motivational theories describe social support as an important precondition for maintaining students' academic motivation; we use SEVT (Eccles & Wigfield, 2020) as it provides a comprehensive theoretical framework to explain achievement‐related behaviour and choices in educational settings. This theory describes expectancies of success and subjective task values (STVs) as proximal drivers of achievement‐related behaviour and choices. Expectancies of success describe the extent to which students believe that they can succeed in a task; this is conceptually similar (albeit different) to other competence‐related beliefs such as self‐efficacy (Wigfield & Eccles, 2000). STV refers to the value a student attaches to a task. According to SEVT, the overall value of a task is comprised of at least the following four components: intrinsic value (i.e. the enjoyment related to engaging in a task), attainment value (i.e. the perceived importance of being good in a task), utility value (i.e. the perceived utility of a task for individual goals) and cost (i.e. the perceived costs of engaging in a task; Eccles & Wigfield, 2020). Research has shown that expectancies and positive STVs predict university students' achievement, course choices and persistence positively, whereas cost predicts these outcomes negatively (e.g. Benden & Lauermann, 2022; Kryshko et al., 2022; Perez et al., 2019; Robinson et al., 2019). SEVT also postulates that the beliefs and behaviours of socializers, such as parents, educators and peers, contribute to the formation of students' academic motivation.

A rich body of literature broadly supports the associations between perceived social support and academic motivation, although more studies have focused on middle and high school (e.g. Ruzek et al., 2016; Song et al., 2015; Wentzel et al., 2010, 2017) than on university (e.g. Freeman et al., 2007; Pan & Gauvain, 2012). A few studies have explicitly investigated the link between social support or belonging and students' expectancies and STVs in university. In a sample with participants from 5th grade until university, Rice et al. (2013) showed that perceived social support by parents, educators and peers were similarly positively correlated with self‐efficacy and STV in math and science in all age groups. Focusing on university freshmen, Freeman et al. (2007) found that class‐level sense of belonging, university‐level belonging, and perceived social acceptance at university (by peers and personnel) were all related to self‐efficacy and STV in a specific course. In one of the few longitudinal studies, Pittman and Richmond (2008) found that university freshmen's positive changes in their sense of belonging in university were associated with positive changes in perceived academic competence. Finally, Rubach et al. (2022a, 2022b) found that perceived academic support from the instructor in university courses predicted later expectancies and STVs in these courses.

These studies provide evidence of how social support and belonging relate to academic motivation in university. However, most studies had a cross‐sectional design (Freeman et al., 2007; Rice et al., 2013) or did not control for the initial levels of motivation (Rubach et al., 2022a, 2022b), which hinders conclusions about directionality. Indeed, longitudinal studies examining the associations between social support and motivation more generally have not only provided evidence for predictive effects of perceived social support on motivation (e.g. Pan & Gauvain, 2012; Pittman & Richmond, 2008; Ruzek et al., 2016; Song et al., 2015) but also for predictive effects of students' motivation on the levels of social support they receive (Skinner & Belmont, 1993) or perceive (Bostwick et al., 2022; Jang et al., 2012). In line with transactional models of development (Sameroff, 2009), students' motivation (e.g. their perceived competence or value in a specific domain) can influence how they engage in class and thus how their instructors and peers interact with them. For instance, instructors might be more willing to provide support to more motivated students, but students might also actively solicit this support more. Insofar, motivation and social support can reinforce each other over time (for similar arguments, see Daumiller & Hemi, 2025; Jang et al., 2012; Skinner & Belmont, 1993).

In addition, there is a lack of previous research investigating multiple dimensions of social support and academic motivation. Although initial evidence suggests that both perceived peer and faculty support are associated with students' academic expectancies and STVs (Freeman et al., 2007; Rice et al., 2013), more research is needed to test whether longitudinal links between social support and academic motivation vary by dimension of social support or motivation.

### At‐risk groups in university and the meaning of social support for them

Social support is important for university students' successful positive academic development (e.g. Arroyo et al., 2021; Gillen‐O'Neel, 2021; Wilcox et al., 2005). However, some groups of students, such as FG and transfer students, can experience disadvantages in their university education, not only in terms of their academic background but also the financial and academic support their families can provide as well as negative stereotypes, discrimination, and social identity threats (Croizet & Claire, 1998; Saenz et al., 2007). Therefore, these students might benefit from social support to compensate for these disadvantages; yet unfortunately, they may actually experience lower levels of support than more advantaged students.

In the United States, 54% of all undergraduate students identified as *FG students* in 2020 (RTI International, 2023). These students could be at risk of being disadvantaged at university due to a lack of knowledge about postsecondary education (Saenz et al., 2007) or because of experiencing a mismatch between the norms from their social backgrounds and the norms prevalent in university (Stephens et al., 2012). FG students might also be less socially integrated in university because they often live with their families and need to work to compensate for having fewer financial resources (Saenz et al., 2007). Therefore, FG students may be challenged in meeting academic demands (Pascarella et al., 2004; Pedler et al., 2022; Tinto, 1993). Empirical evidence regarding FG students' perceived social support and sense of belonging is mixed—some studies indicated no differences between FG and continuing generation (CG) students, whereas others found differences favouring CG students (Gillen‐O'Neel, 2021; McPartlan et al., 2020; Pedler et al., 2022; Stebleton et al., 2014).

*Transfer students* usually spend their first year(s) in higher education at a (two‐year) community college before transitioning to a four‐year institution. The number of transfer students is growing, with 13.2% of all undergraduate students in the United States representing transfer students in Fall 2021 (Gardner et al., 2024). Notably, social support structures for these transitions are understudied in the field. From interviews, Shaw and Chin‐Newman (2017) found that transfer students face enormous challenges after transitioning to a four‐year university and that various forms of social support can help in adjusting to this new environment. Thus, transfer students may experience difficulties when entering this new academic environment, encountering different academic norms, especially as they enter as juniors and thus have less time to integrate themselves compared to traditional university freshmen.

Social support might help these groups of students and, therefore, associations between perceived social support and academic motivation could be more pronounced for FG and transfer students compared to their more privileged peers. Some studies found evidence for a more important role of social support and belonging for students' academic development for disadvantaged students. For instance, a study examining day‐to‐day fluctuations in sense of belonging found that FG students' in‐class engagement was especially sensitive to such fluctuations in sense of belonging (Gillen‐O'Neel, 2021). Moreover, based on a large longitudinal data set, Pascarella et al. (2004) showed that both perceived academic and social integration in university were more important for FG students' university success compared with CG students (for similar findings, see Lohfink & Paulsen, 2005). Along similar lines of argument, interventions to increase sense of belonging have been found to help racial minority and FG students but not their more socially advantaged peers (Murphy et al., 2020; Walton & Cohen, 2007; Yeager et al., 2016). To the best of our knowledge, prior research has not compared the role of social support for students' academic development between transfer and non‐transfer students. More research is thus needed to systematically examine whether the associations between social support and academic motivation differ between subgroups of students while also considering different dimensions of social support.

### The present study

In the present study, we examined the longitudinal associations between students' confidence in getting social support and their course‐specific expectancies of success and STVs collected across several time points of the academic term. We focused on expectancies and STVs in two types of courses: their most difficult course and their most important course, as determined by the students (see Rubach et al., 2022a, for students' rationale for their ratings). This approach allowed us to examine differences across courses for generalization. Moreover, social support might be particularly relevant in these courses. Students reported lower expectancies in their most difficult course and perceived content in this course as overwhelming. In their most important course, students reported higher STV, but these courses were also often identified as important because they were a requirement for their major, which can come along with high pressure.

We used data from two cohorts of freshman and junior students at a diverse U.S. university collected in Fall 2019 and 2020, which allowed us to also test the generalizability of such associations across in‐person and virtual learning settings. Although students in this sample reported lower confidence in getting support in virtual learning settings (Von Keyserlingk et al., 2024), we did not have specific expectations about potential differences in the associations between confidence in getting support and academic motivation across these settings.

We examined the following research questions (RQs):

1. To what extent are there differences in confidence in getting support between FG students, transfer students and their peers?
  Prior research has found mixed evidence concerning social support for FG and CG students (Gillen‐O'Neel, 2021; McPartlan et al., 2020; Pedler et al., 2022; Stebleton et al., 2014). Therefore, we examined this RQ in an exploratory manner.
2. Is confidence in getting support by faculty and peers reciprocally linked with academic expectancies and STVs over time?
  Based on the theoretical importance of the availability of social support and prior research (e.g. Freeman et al., 2007; Rubach et al., 2022a, 2022b), we predicted that higher confidence in getting support would be associated with higher academic expectancies and STVs at later time points. Because some prior research provided evidence for reciprocal effects between social support and academic motivation (e.g. Jang et al., 2012), we also explored the predictive effects of these expectancies and STVs on later confidence in getting support. Due to a lack of research investigating longitudinal associations between multiple dimensions of social support and academic expectancies and STVs, we did not have differentiated hypotheses for peer and faculty support.
3. Is confidence in getting support by faculty and peers differentially associated with academic expectancies and STVs for student subgroups?
  Based on prior research (e.g. Gillen‐O'Neel, 2021; Pascarella et al., 2004), we predicted more pronounced associations amongst FG and transfer students than amongst their peers (i.e. CG and non‐transfer students).

## METHOD

### Sample and procedure

Data were collected as part of a large longitudinal project, the UCI Measuring Undergraduate Success Trajectories Project (https://sites.uci.edu/ucimustproject/) at a large, diverse public research university in Southern California.^1^ Notably, this university is federally designated as a Hispanic‐Serving Institution and Asian American and Native American Pacific Islander‐Serving Institution. The overall study was designed to investigate undergraduates' experiences and success and received Institutional Review Board approval.

In 2019 and 2020, all undergraduate students beginning their freshman or junior year were invited via email to participate in the study. In 2019, 1275 students consented to participate in the project. A subsample of 359 students agreed to participate in additional weekly web‐based surveys over the academic year. In the current study, we used data from the weekly surveys in the Fall 2019 term for 320 students (cohort 1) who completed at least one of the scales relevant to our RQs. In 2020, 1323 students consented to participate in the project, of which 498 students consented to participate in the additional weekly surveys. A total of 417 students completed at least one of the scales relevant to our RQs in the weekly surveys in the Fall 2020 term and thus formed the subsample we used from cohort 2. Sample characteristics (including gender and ethnicity) for both cohorts are reported in Table 1 (for a comparison between the study sample and the undergraduate student university population, see the Supplemental Materials). Whereas learning in Fall 2019 happened in person, learning in Fall 2020 was fully remote, and campus housing was not available to students due to the COVID‐19 pandemic.

**TABLE 1:** Sample demographics for Fall 2019 and Fall 2020 data.

	Fall 2019 (*n* = 320)		Fall 2020 (*n =* 417)	
	*n*	%	*n*	%
Gender				
Female	219	68.4	299	71.7
Male	100	31.3	115	27.6
Grade level				
Freshman	221	69.1	249	59.7
Junior	99	30.9	167	40.1
Transfer student status				
Transfer student	65	20.3	102	24.5
Non‐transfer student	255	79.7	314	75.3
University‐going generation				
CG student	136	42.5	172	41.2
FG student	180	56.3	224	53.7
Race/ethnicity				
Asian American	151	47.2	201	48.2
Latinx American	111	34.7	131	32.0
European American	40	12.5	59	14.1
Other	10	3.1	19	4.6

### Instruments

Table 2 presents sample items and scale reliabilities. More information about the instruments, including results of confirmatory factor analyses and measurement invariance, can be found in the Supplemental Materials. The instruments and the timing were the same for the Fall 2019 and Fall 2020 assessments, unless noted otherwise.

**TABLE 2:** Sample items and reliabilities (Cronbach's alpha) for all study scales.

Scale	Sample item	Fall 2019			Fall 2020		
		*α*_T1_	*α*_T2_	*α*_T3_	*α*_T1_	*α*_T2_	*α*_T3_
Peer support	How confident are you that you could call another student if you had a question about an assignment?	.88	.87	–	.81	.86	–
Faculty support	How confident are you that a faculty member would take the time to talk to you if you needed help?	.87	.90	–	.89	.91	–
Expectancy	How good do you think you will be at learning the new material in X?	.84/.84	.92/.92	.86/.90	.80/.87	.90/.91	.88/.91
Positive STV	How much do you expect that X will be important to you personally in terms of your values and identities?	.86/.83	.88/.86	.89/.88	.87/.87	.88/.90	.90/.91
Cost	How much do you expect that X will be stressful?	.63/.74	.74/.84	.71/.75	.58/.75	.73/.82	.70/.80

*Note*: For expectancy, positive STV, and cost, values before the dash represent Cronbach's alpha in students' most difficult course, and values behind the dash represent Cronbach's alpha in students' most important course. The wording of these items varied depending on the time in the academic term.

Confidence in getting support by peers and faculty was assessed in weeks 2 and 6 of the term, and thus in the beginning (T1) and middle (T2) of the academic term. The items were mostly taken from a well‐established scale measuring social support in university (Hoffman et al., 2002).

Students rated their academic expectancies and their STVs for their subjectively most difficult and most important courses^2^ in the first week of the term (T1), after they had received their midterm results (T2), and in the first week of the next term after they received their final grades (T3). During weeks 6/5 (in Fall 2019/2020) to 9/8, students responded to the respective items after receiving their midterm results. Items were worded specifically to refer to the time point in the academic term. At T1, items were asked in a way to assess students' expectancies and STVs before engaging much in the course. At T2, students were asked to report on their expectancies and STVs based on experiences so far in the course. At T3, students rated their expectancies and STVs considering all their experiences in the course.

Information about student demographics, including FG and transfer student status, race/ethnicity, high school grade point average (GPA) and gender, was taken from the university's administrative data.

### Statistical analyses

Because of our somewhat limited sample size, particularly when it comes to subgroup analyses, we relied on manifest scale scores for all our statistical analyses addressing RQ1–3. For RQ1, we tested mean differences at T1 and T2 in confidence in getting support between subgroups of students using independent samples *t*‐tests. For RQ2 and RQ3, we conducted path analyses in Mplus 8.2 (Muthén & Muthén, 1998–2017). These models used robust standard errors to deal with potential nonnormality of the data. All data analysis scripts are available at https://osf.io/4ct2g/.

#### Linking confidence in getting support with academic expectancies and STVs

**FIGURE 1:**
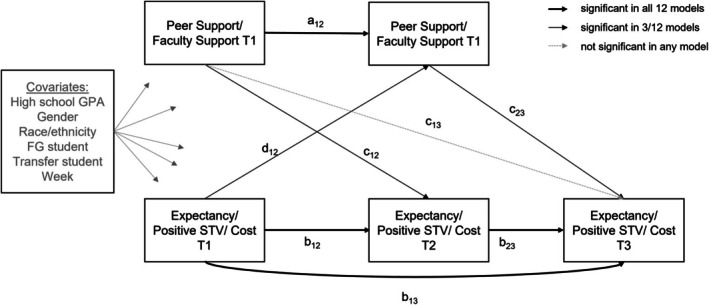
Visual summary of the results of path models examining longitudinal associations between social support and expectancies and STVs. Social support, expectancies, and STVs at T1 to T3 were regressed on all covariates. Covariances were allowed between the covariates as well as the constructs assessed at the same time point; these covariances are not depicted in the sake of clarity. FG, first‐generation; GPa, grade point average; STV, subjective task value.

For RQ2, we analysed the reciprocal associations between students' confidence in getting support and their expectancies and STVs across time (Figure 1). We used traditional cross‐lagged panel models, in which confidence in getting support and motivation at later time points were regressed onto confidence in getting support and motivation at earlier time points, and thus focused on predictive effects of interindividual differences in one construct (e.g. confidence in getting support) onto changes in another construct (e.g. motivation) compared with other students. These models were fully saturated. We specified one model each for the different combinations of courses (most difficult and most important course), dimensions of social support (peer and faculty support), and motivational components (expectancies, positive STVs, and cost), resulting in 12 models for each cohort. Separate models for dimensions of social support and motivation were computed to avoid overly complex models. In all models, we controlled for students' high school GPA, gender, race/ethnicity, FG student status and transfer student status. Finally, we included three dummy variables as control variables indicating the respective week in which students reported on their midterm expectancies and STVs (weeks 7 through 9/6 through 8, with week 6/5 as the reference category in the Fall 2019/Fall 2020 data). All continuous variables were standardized across individuals before conducting the analyses so that all regression coefficients can be understood as standardized regression coefficients with respect to the variance in the outcome variables.

#### Multi‐group analyses: Examining differential associations for student subgroups

For RQ3, we tested for differences in the stability and cross‐lagged coefficients between student subgroups. For this purpose, we conducted multi‐group analyses comparing FG and CG students and transfer and non‐transfer students, respectively. In line with the statistical analyses for RQ2, we controlled for students' high school GPA, gender, race/ethnicity, as well as FG and transfer student status if they varied between the groups in the focus of the analysis. Because of the limited sample sizes per group, we simplified the model and did not include the three dummy variables for the respective weeks as covariates in these analyses. The effects of the covariates were freely estimated between subgroups. Differences regarding the stability and cross‐lagged coefficients between groups were tested using the model constraint in Mplus, which relies on the delta method (Raykov & Marcoulides, 2004).

#### Missing data

Missing data for the social support and SEVT scales assessed at T1 to T3 ranged from 3.4% to 19.4% in the Fall 2019 data and from 6.2% to 25.4% in the Fall 2020 data. Further investigation of missing data showed that, in the Fall 2019 data, students with missing data at one of the time points differed in terms of their race/ethnicity (*χ*(3) = 9.16, *p* = .027) and showed a lower high school GPA (*t*(317) = 2.44, *p* = .015) compared with students with data available at all time points. No differences were found in terms of FG student status, transfer student status, and gender. In the Fall 2020 data, there were no significant differences for any of these variables. In line with recommendations to deal with missing data in longitudinal research (Graham, 2009), we used the full information maximum likelihood approach implemented in Mplus for our path analyses, which takes all available information into account when the model parameters are estimated. We furthermore included high school GPA, gender, race/ethnicity, FG student status, and transfer student status as covariates in our analyses to make the missing at random assumption more plausible (Collins et al., 2001).

## RESULTS

### Differences in confidence in getting support between student subgroups

We observed little differences in confidence in getting support between student subgroups (see Table 3). When comparing FG and CG students, all observed differences were small (*d* = −0.19 to 0.19) and non‐significant. Differences between transfer and non‐transfer students were also small (*d* = −0.16 to 0.08) and non‐significant.

**TABLE 3:** Mean differences in confidence in getting support between subgroups of students.

Fall 2019	FG student			CG student			*t*	*df*	*p*	Cohen's *d*
	*N*	*M*	*SD*	*N*	*M*	*SD*				
Peer support T1	171	62.2	28.3	130	59.6	28.9	0.77	299	.440	0.09
Faculty support T1	170	57.4	22.6	130	56.9	19.8	0.20	298	.841	0.02
Peer support T2	164	66.4	28.1	125	61.0	29.2	1.58	287	.116	0.19
Faculty support T2	163	54.4	24.0	125	55.2	22.9	−0.30	286	.768	−0.04

Fall 2019	Transfer student			Non‐transfer student			*t*	*df*	*p*	Cohen's *d*
	*N*	*M*	*SD*	*N*	*M*	*SD*				
Peer support T1	62	61.0	29.8	243	61.4	28.2	−0.12	303	.906	−0.02
Faculty support T2	62	56.8	23.1	242	57.7	21.1	−0.30	302	.766	−0.04
Peer support T1	60	64.4	27.3	233	64.3	28.9	0.02	291	.985	0.00
Faculty support T2	60	56.3	23.4	232	54.5	23.4	0.52	290	.605	0.08

Fall 2020	FG student			CG student						
	*N*	*M*	*SD*	*N*	*M*	*SD*	*t*	*df*	*p*	Cohen's *d*
Peer support T1	198	43.5	28.8	154	43.4	26.9	0.02	350	.983	0.00
Faculty support T1	201	51.6	24.0	155	53.3	23.3	−0.65	354	.516	−0.07
Peer support T2	189	40.4	30.5	149	41.5	30.1	−0.33	336	.741	−0.04
Faculty support T2	192	45.7	24.5	149	50.4	25.5	−1.73	339	.084	−0.19

Fall 2020	Transfer student			Non‐transfer student						
	*N*	*M*	*SD*	*N*	*M*	*SD*	*t*	*df*	*p*	Cohen's *d*
Peer support T1	92	40.2	31.9	275	44.7	26.9	−1.23^a^	137.0	.221	−0.16
Faculty support T1	93	50.7	26.6	278	52.6	22.6	−0.61^a^	138.9	.546	−0.08
Peer support T2	87	41.2	31.5	268	40.7	29.9	0.13	353	.894	0.02
Faculty support T2	88	47.9	27.2	270	48.0	24.5	−0.03	356	.979	0.00

Abbreviations: CG, continuing‐generation; FG, first‐generation.

^a^ Corrected *t*‐values are reported because a Levene test indicated that variances differed significantly (*p* < .05) between groups.

#### Linking confidence in getting support with academic expectancies and STVs

Results for path models investigating reciprocal associations between confidence in getting support and students' expectancies and STVs are presented in Table 4 (Fall 2019) and Table 5 (Fall 2020). We report only the stability and cross‐lagged coefficients here; the results for the full models including covariates can be found in the Supplemental Materials. Overall, social support and academic motivation scales showed substantial stabilities over time, although stabilities were higher for the social support (*β* = 0.63 to 0.72) than for academic motivation measures (*β* = 0.23 to 0.56).

**TABLE 4:** Stability and cross‐lagged coefficients from path models in the Fall 2019 data.

	Most difficult course						Most important course					
	Peer support			Faculty support			Peer support			Faculty support		
	*β*	95% CI	*p*	*β*	95% CI	*p*	*β*	95% CI	*p*	*β*	95% CI	*p*
Expectancy												
Stability coefficients												
Support T1 ➔ Support T2 (a_12_)	**0.71**	**[0.63, 0.80]**	**<.001**	**0.63**	**[0.53, 0.72]**	**<.001**	**0.71**	**[0.62, 0.79]**	**<.001**	**0.64**	**[0.55, 0.74]**	**<.001**
Expectancy T1 ➔ Expectancy T2 (b_12_)	**0.28**	**[0.14, 0.42]**	**<.001**	**0.25**	**[0.11, 0.39]**	**<.001**	**0.28**	**[0.17, 0.40]**	**<.001**	**0.27**	**[0.16, 0.38]**	**<.001**
Expectancy T1 ➔ Expectancy T3 (b_13_)	**0.25**	**[0.11, 0.38]**	**<.001**	**0.25**	**[0.10, 0.39]**	.**001**	**0.27**	**[0.11, 0.43]**	.**001**	**0.26**	**[0.11, 0.41]**	.**001**
Expectancy T2 ➔ Expectancy T3 (b_23_)	**0.29**	**[0.15, 0.42]**	**<.001**	**0.28**	**[0.14, 0.42]**	**<.001**	**0.27**	**[0.16, 0.38]**	**<.001**	**0.27**	**[0.16, 0.38]**	**<.001**
Cross‐lagged coefficients												
Support T1 ➔ Expectancy T2 (c_12_)	0.01	[−0.12, 0.13]	.924	0.10	[−0.03, 0.23]	.124	−0.01	[−0.12, 0.11]	.895	**0.13**	**[0.00, 0.25]**	.**045**
Support T1 ➔ Expectancy T3 (c_13_)	0.10	[−0.07, 0.27]	.244	0.03	[−0.13, 0.19]	.703	0.00	[−0.16, 0.17]	.983	−0.13	[−0.28, 0.02]	.097
Support T2 ➔ Expectancy T3 (c_23_)	−0.11	[−0.27, 0.05]	.189	0.01	[−0.13, 0.14]	.939	0.06	[−0.10, 0.22]	.470	**0.20**	**[0.06, 0.34]**	.**005**
Expectancy T1 ➔ Support T2 (d_12_)	0.02	[−0.07, 0.12]	.655	**0.11**	**[0.02, 0.21]**	.**024**	0.02	[−0.06, 0.11]	.579	0.09	[−0.01, 0.18]	.079
Positive STV												
Stability coefficients												
Support T1 ➔ Support T2 (a_12_)	**0.71**	**[0.62, 0.80]**	**<.001**	**0.64**	**[0.55, 0.74]**	**<.001**	**0.69**	**[0.61, 0.78]**	**<.001**	**0.65**	**[0.55, 0.74]**	**<.001**
Positive STV T1 ➔ Positive STV T2 (b_12_)	**0.56**	**[0.45, 0.67]**	**<.001**	**0.55**	**[0.44, 0.67]**	**<.001**	**0.52**	**[0.42, 0.62]**	**<.001**	**0.51**	**[0.41, 0.61]**	**<.001**
Positive STV T1 ➔ Positive STV T3 (b_13_)	**0.37**	**[0.22, 0.53]**	**<.001**	**0.38**	**[0.22, 0.54]**	**<.001**	**0.39**	**[0.27, 0.50]**	**<.001**	**0.38**	**[0.27, 0.50]**	**<.001**
Positive STV T2 ➔ Positive STV T3 (b_23_)	**0.27**	**[0.13, 0.41]**	**<.001**	**0.25**	**[0.09, 0.40]**	.**002**	**0.41**	**[0.30, 0.52]**	**<.001**	**0.41**	**[0.30, 0.53]**	**<.001**
Cross‐lagged coefficients												
Support T1 ➔ Positive STV T2 (c_12_)	0.04	[−0.07, 0.16]	.444	0.07	[−0.05, 0.20]	.256	0.10	[−0.02, 0.22]	.106	0.11	[0.00, 0.22]	.059
Support T1 ➔ Positive STV T3 (c_13_)	0.10	[−0.05, 0.25]	.200	0.02	[−0.12, 0.15]	.811	0.10	[−0.04, 0.23]	.156	−0.03	[−0.14, 0.08]	.581
Support T2 ➔ Positive STV T3 (c_23_)	−0.07	[−0.23, 0.09]	.373	0.08	[−0.05, 0.22]	.224	−0.06	[−0.18, 0.06]	.324	0.05	[−0.06, 0.16]	.353
Positive STV T1 ➔ Support T2 (d_12_)	0.06	[−0.03, 0.14]	.188	0.04	[−0.06, 0.13]	.469	**0.11**	**[0.03, 0.20]**	.**011**	0.04	[−0.06, 0.14]	.457
Cost												
Stability coefficients												
Support T1 ➔ Support T2 (a_12_)	**0.72**	**[0.64, 0.81]**	**<.001**	**0.65**	**[0.56, 0.74]**	**<.001**	**0.71**	**[0.63, 0.80]**	**<.001**	**0.65**	**[0.56, 0.74]**	**<.001**
Cost T1 ➔ Cost T2 (b_12_)	**0.42**	**[0.29, 0.54]**	**<.001**	**0.41**	**[0.29, 0.54]**	**<.001**	**0.53**	**[0.41, 0.65]**	**<.001**	**0.53**	**[0.41, 0.65]**	**<.001**
Cost T1 ➔ Cost T3 (b_13_)	**0.42**	**[0.29, 0.56]**	**<.001**	**0.41**	**[0.27, 0.55]**	**<.001**	**0.37**	**[0.24, 0.50]**	**<.001**	**0.39**	**[0.26, 0.51]**	**<.001**
Cost T2 ➔ Cost T3 (b_23_)	**0.26**	**[0.13, 0.39]**	**<.001**	**0.26**	**[0.13, 0.39]**	**<.001**	**0.31**	**[0.20, 0.42]**	**<.001**	**0.31**	**[0.20, 0.42]**	**<.001**
Cross‐lagged coefficients												
Support T1 ➔ Cost T2 (c_12_)	−0.02	[−0.13, 0.09]	.702	−0.04	[−0.15, 0.07]	.496	0.01	[−0.09, 0.11]	.877	0.01	[−0.10, 0.11]	.884
Support T1 ➔ Cost T3 (c_13_)	−0.03	[−0.18, 0.11]	.640	0.02	[−0.13, 0.16]	.804	0.08	[−0.08, 0.24]	.307	0.11	[−0.04, 0.26]	.160
Support T2 ➔ Cost T3 (c_23_)	**0.18**	**[0.03, 0.33]**	.**019**	0.09	[−0.04, 0.22]	.155	−0.01	[−0.16, 0.14]	.892	−0.04	[−0.20, 0.12]	.654
Cost T1 ➔ Support T2 (d_12_)	**0.09**	**[0.00, 0.17]**	.**041**	−0.04	[−0.15, 0.06]	.406	0.06	[−0.02, 0.14]	.143	−0.07	[−0.16, 0.02]	.149

*Note*: Coefficients with *p* < .05 are highlighted in bold. Path models included high school GPA, gender, ethnicity/race, FG student status, transfer student status, and the week in which SEVT constructs were assessed at T2 as control variables.

**TABLE 5:** Stability and cross‐lagged coefficients from path models in the Fall 2020 data.

	Most difficult course						Most important course					
	Peer support			Faculty support			Peer support			Faculty support		
	*β*	95% CI	*p*	*β*	95% CI	*p*	*β*	95% CI	*p*	*β*	95% CI	*p*
Expectancy												
Stability coefficients												
Support T1 ➔ Support T2 (a_12_)	**0.71**	**[0.64, 0.78]**	**<.001**	**0.63**	**[0.53, 0.73]**	**<.001**	**0.72**	**[0.65, 0.79]**	**<.001**	**0.65**	**[0.56, 0.74]**	**<.001**
Expectancy T1 ➔ Expectancy T2 (b_12_)	**0.27**	**[0.15, 0.38]**	**<.001**	**0.23**	**[0.10, 0.35]**	**<.001**	**0.41**	**[0.32, 0.50]**	**<.001**	**0.39**	**[0.30, 0.48]**	**<.001**
Expectancy T1 ➔ Expectancy T3 (b_13_)	**0.43**	**[0.32, 0.53]**	**<.001**	**0.42**	**[0.31, 0.52]**	**<.001**	**0.53**	**[0.42, 0.64]**	**<.001**	**0.55**	**[0.44, 0.66]**	**<.001**
Expectancy T2 ➔ Expectancy T3 (b_23_)	**0.34**	**[0.24, 0.44]**	**<.001**	**0.31**	**[0.21, 0.42]**	**<.001**	**0.18**	**[0.07, 0.29]**	.**001**	**0.18**	**[0.07, 0.29]**	.**002**
Cross‐lagged coefficients												
Support T1 ➔ Expectancy T2 (c_12_)	0.08	[−0.04, 0.20]	.185	**0.17**	**[0.04, 0.30]**	.**008**	0.02	[−0.09, 0.13]	.693	**0.15**	**[0.05, 0.25]**	.**005**
Support T1 ➔ Expectancy T3 (c_13_)	−0.06	[−0.20, 0.09]	.441	0.06	[−0.06, 0.18]	.302	0.14	[0.00, 0.29]	.057	−0.05	[−0.19, 0.08]	.457
Support T2 ➔ Expectancy T3 (c_23_)	0.08	[−0.07, 0.23]	.316	0.04	[−0.08, 0.16]	.493	−0.12	[−0.26, 0.03]	.121	0.00	[−0.13, 0.13]	.982
Expectancy T1 ➔ Support T2 (d_12_)	0.02	[−0.05, 0.09]	.565	0.05	[−0.06, 0.14]	.379	−0.01	[−0.09, 0.07]	.767	0.04	[−0.05, 0.12]	.428
Positive STV												
Stability coefficients												
Support T1 ➔ Support T2 (a_12_)	**0.72**	**[0.65, 0.79]**	**<.001**	**0.64**	**[0.55, 0.73]**	**<.001**	**0.72**	**[0.65, 0.79]**	**<.001**	**0.66**	**[0.57, 0.75]**	**<.001**
Positive STV T1 ➔ Positive STV T2 (b_12_)	**0.61**	**[0.53, 0.69]**	**<.001**	**0.60**	**[0.52, 0.68]**	**<.001**	**0.61**	**[0.53, 0.69]**	**<.001**	**0.59**	**[0.51, 0.68]**	**<.001**
Positive STV T1 ➔ Positive STV T3 (b_13_)	**0.42**	**[0.30, 0.54]**	**<.001**	**0.41**	**[0.29, 0.53]**	**<.001**	**0.60**	**[0.49, 0.71]**	**<.001**	**0.61**	**[0.49, 0.72]**	**<.001**
Positive STV T2 ➔ Positive STV T3 (b_23_)	**0.38**	**[0.27, 0.49]**	**<.001**	**0.37**	**[0.26, 0.49]**	**<.001**	**0.24**	**[0.14, 0.34]**	**<.001**	**0.25**	**[0.14, 0.35]**	**<.001**
Cross‐lagged coefficients												
Support T1 ➔ Positive STV T2 (c_12_)	−0.01	[−0.10, 0.08]	.883	0.04	[−0.05, 0.13]	.376	0.05	[−0.03, 0.14]	.230	0.08	[0.00, 0.17]	.055
Support T1 ➔ Positive STV T3 (c_13_)	−0.09	[−0.20, 0.04]	.164	0.01	[−0.11, 0.13]	.836	0.00	[−0.14, 0.15]	.991	−0.02	[−0.13, 0.10]	.792
Support T2 ➔ Positive STV T3 (c_23_)	0.07	[−0.05, 0.19]	.266	0.06	[−0.06, 0.17]	.343	0.02	[−0.11, 0.16]	.739	−0.01	[−0.12, 0.09]	.814
Positive STV T1 ➔ Support T2 (d_12_)	−0.04	[−0.11, 0.04]	.326	0.04	[−0.04, 0.13]	.344	0.01	[−0.07, 0.09]	.860	0.00	[−0.09, 0.08]	.951
Cost												
Stability coefficients												
Support T1 ➔ Support T2 (a_12_)	**0.71**	**[0.64, 0.78]**	**<.001**	**0.64**	**[0.56, 0.73]**	**<.001**	**0.72**	**[0.65, 0.79]**	**<.001**	**0.66**	**[0.57, 0.74]**	**<.001**
Cost T1 ➔ Cost T2 (b_12_)	**0.37**	**[0.27, 0.47]**	**<.001**	**0.37**	**[0.27, 0.47]**	**<.001**	**0.31**	**[0.21, 0.42]**	**<.001**	**0.31**	**[0.21, 0.42]**	**<.001**
Cost T1 ➔ Cost T3 (b_13_)	**0.41**	**[0.31, 0.52]**	**<.001**	**0.41**	**[0.30, 0.52]**	**<.001**	**0.56**	**[0.46, 0.67]**	**<.001**	**0.56**	**[0.46, 0.66]**	**<.001**
Cost T2 ➔ Cost T3 (b_23_)	**0.31**	**[0.20, 0.42]**	**<.001**	**0.31**	**[0.20, 0.43]**	**<.001**	**0.24**	**[0.14, 0.35]**	**<.001**	**0.24**	**[0.14, 0.35]**	**<.001**
Cross‐lagged coefficients												
Support T1 ➔ Cost T2 (c_12_)	−0.01	[−0.11, 0.09]	.869	0.01	[−0.10, 0.12]	.896	0.01	[−0.09, 0.12]	.797	0.01	[−0.10, 0.11]	.926
Support T1 ➔ Cost T3 (c_13_)	0.07	[−0.07, 0.22]	.313	−0.05	[−0.18, 0.07]	.404	−0.07	[−0.20, 0.06]	.305	−0.06	[−0.18, 0.07]	.363
Support T2 ➔ Cost T3 (c_23_)	−0.13	[−0.27, 0.02]	.082	0.01	[−0.13, 0.14]	.945	**0.13**	**[0.01, 0.25]**	.**036**	0.04	[−0.09, 0.16]	.531
Cost T1 ➔ Support T2 (d_12_)	−0.01	[−0.09, 0.07]	.784	−0.01	[−0.09, 0.08]	.861	0.06	[−0.02, 0.13]	.148	0.01	[−0.07, 0.09]	.836

*Note*: Coefficients with *p* < .05 are highlighted in bold. Path models included high school GPA, gender, ethnicity/race, FG student status, transfer student status, and the week in which SEVT constructs were assessed at T2 as control variables.

In Fall 2019, we found a few significant cross‐lagged effects between confidence in getting support and academic motivation. Confidence in getting peer support was not reciprocally associated with expectancies, but the results revealed some associations for confidence in getting faculty support. In students' most difficult course, expectancies at T1 predicted higher confidence in getting faculty support at T2 (*β* = 0.11). In students' most important course, confidence in getting faculty support at T1 predicted higher expectancies at T2 (*β* = 0.13) and T3 (*β* = 0.20). Findings showed little evidence regarding reciprocal associations between confidence in getting support and positive STVs. Only in students' most important course did positive STVs at T1 predict higher confidence in getting peer support at T2 (*β* = 0.11). Regarding reciprocal effects with cost, in students' most difficult course, confidence in getting peer support at T1 predicted higher perceived cost at T3 (*β* = 0.18), and higher cost at T1 predicted higher confidence in getting peer support at T2 (*β* = 0.09).

In Fall 2020, we observed three significant cross‐lagged effects. In both students' most difficult and important course, confidence in getting faculty support at T1 predicted higher expectancies at T2 (*β* = 0.17 and *β* = 0.15, respectively). Moreover, confidence in getting peer support at T1 predicted higher perceived cost at T3 in students' most important course (*β* = 0.13). That is, not all cross‐lagged effects were replicated in the Fall 2020 data, although the overall pattern was similar across cohorts.^3^

#### Multi‐group analyses: Examining differential associations for student subgroups

**FIGURE 2:**
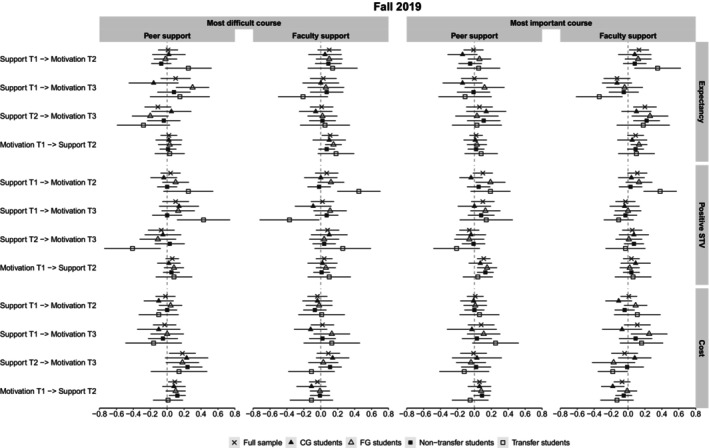
Cross‐lagged coefficients for the total sample and different subgroups in the Fall 2019 data.

**FIGURE 3:**
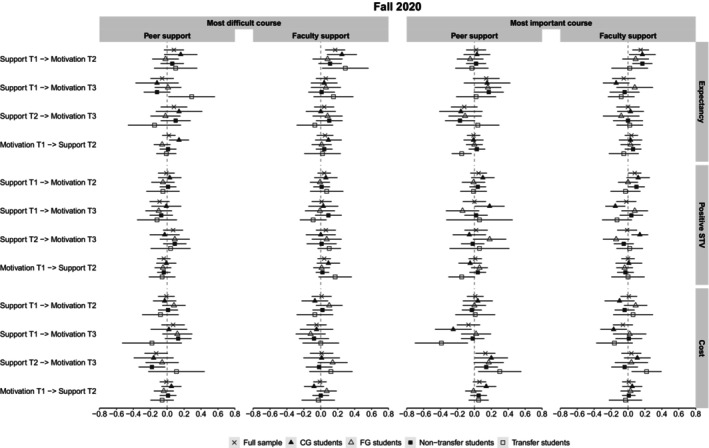
Cross‐lagged coefficients for the total sample and different subgroups in the Fall 2020 data.

Figure 2 displays the cross‐lagged coefficients for the entire sample and for all subgroups in the Fall 2019 data. Similarly, Figure 3 displays these coefficients in Fall 2020. The stability and cross‐lagged coefficients for each subgroup and the differences in these coefficients between the respective subgroups are reported in the Supplemental Materials.

From the 72 comparisons (per cohort) between cross‐lagged coefficients from confidence in getting support to later motivation for which we expected differential associations across subgroups, 9 (12.5%) were statistically significant in the Fall 2019 data (FG vs. CG: 8.3%, Transfer vs. non‐Transfer: 16.7%) and 6 were significant (8.3%) in the Fall 2020 data (FG vs. CG: 8.3%, Transfer vs. non‐Transfer: 8.3%). We further describe differences in the coefficients that were significant.

Comparing FG and CG students in *Fall 2019*, confidence in getting peer support at T2 positively predicted FG students' expectancies at T3 in their most difficult course (*β* = 0.30), whereas this was not the case for CG students (*β* = −0.16). Moreover, confidence in getting peer support at T1 predicted FG students' positive STVs at T2 in students' most important course (*β* = 0.19), which was not the case for CG students (*β* = −0.04). Finally, confidence in getting faculty support at T2 predicted higher cost in students' most important course at T3 for FG students (*β* = 0.25), which again was not observed for CG students (*β* = −0.07). In *Fall 2020*, in students' most important course, confidence in getting peer support at T2 predicted positive STVs at T3 for CG students (*β* = 0.18), but not for FG students (*β* = −0.14). Moreover, in students' most important course, confidence in getting faculty support at T2 negatively predicted positive STVs at T3 for CG students (*β* = −0.15), while confidence in getting faculty support at T1 positively predicted positive STVs at T3 for these students (*β* = 0.14). Both coefficients were non‐significant for FG students (*β* = 0.08 and *β* = −0.14, respectively).

Comparing transfer and non‐transfer students in the *Fall 2019* data, confidence in getting peer support at T1 predicted transfer students' expectancies in their most difficult course at T2 (*β* = 0.25), which was not the case for non‐transfer students (*β* = −0.07). Similarly, confidence in getting peer support at T2 predicted students' positive STVs in their most difficult course at T3 for transfer students (*β* = 0.43), but not for non‐transfer students (*β* = 0.00). However, confidence in getting peer support at T1 negatively predicted transfer students' positive STVs in their most difficult course at T3 (*β* = −0.41), which was again not found for non‐transfer students (*β* = 0.03). Confidence in getting faculty support at T1 positively predicted transfer students' positive STVs at T2 in their most difficult (*β* = 0.45) and their most important course (*β* = 0.38), which was not the case for non‐transfer students (*β* = −0.02 and *β* = 0.03, respectively). However, in students' most difficult course, confidence in getting faculty support at T2 negatively predicted transfer students' positive STVs at T3 (*β* = −0.37), which, again, was not the case for non‐transfer students (*β* = 0.07). In the *Fall 2020* data, confidence in getting peer support at T2 predicted higher expectancies in students' most difficult course at T3 for transfer students (*β* = 0.29) but not for non‐transfer students (*β* = −0.12). Confidence in getting peer support at T2 also negatively predicted cost in students' most important course at T3 for transfer students (*β* = −0.39) but not for non‐transfer students (*β* = −0.02). Finally, confidence in getting faculty support at T1 positively predicted cost in students' most important course at T3 for transfer students (*β* = 0.22) but not for non‐transfer students (*β* = −0.04).

Overall, there was some evidence for stronger associations between confidence in getting support and later motivation for FG and particularly for transfer students. Many of these differences suggest that FG and transfer students may benefit more from high confidence in getting support.

## DISCUSSION

Social support is often assumed to play a key role in students' academic development in university, particularly for more disadvantaged students, such as FG and transfer students. Based on data from repeated surveys in a diverse sample of university students, we investigated the longitudinal, reciprocal associations between different dimensions of confidence in getting support and course‐specific expectancies and STVs. Our findings provide some support for small reciprocal associations when considering the whole sample. This is noteworthy insofar as prior research in university settings has not sufficiently relied on longitudinal data and has not investigated the potential influence of academic motivation on social support. Contrary to expectations and some prior research, the FG and transfer students in this study did not report lower confidence in getting support overall. However, in line with expectations, we observed more pronounced associations between students' confidence in getting support and their later motivation for and especially for transfer students. Overall, the findings were similar for the Fall 2019 and Fall 2020 cohorts, which speak for the generalizability of these findings across regular in‐person and pandemic‐affected virtual learning settings.

### Reciprocal associations between confidence in getting support and expectancies, and STVs

To the best of our knowledge, our study is the first to demonstrate evidence for reciprocal associations between perceived social support and academic motivation in university. Our study provides evidence that students who perceive higher levels of social support by faculty also tend to develop higher expectancies and STVs over time, which aligns with SEVT (Eccles & Wigfield, 2020) and student integration theory (Tinto, 1993). For university, this means that interventions that strengthen students' confidence in getting social support when needed could boost students' academic motivation and thus their study success. To this end, students should know where and how they can access support and feel comfortable asking for it. However, students with high initial expectancies and STVs can also develop stronger confidence in getting social support over time. Students who are highly motivated academically from the beginning may perceive faculty and peers more positively but might also be more likely to solicit supportive behaviour in others, ask for support themselves, and be more confident in receiving such support (Jang et al., 2012; Karabenick & Berger, 2013; Skinner & Belmont, 1993). Notably, we assessed students' confidence in getting support if needed rather than actual support. Although we believe this is psychologically more meaningful, it is therefore unclear whether the reciprocal effects we found also translate into actual social support. However, some prior research shows that school teachers provide more support to engaged students (Skinner & Belmont, 1993).

Concerning the different dimensions of social support, we found that confidence in getting faculty support was more closely associated with expectancies and positive STVs than peer support. This aligns with the assumptions of Tinto (1993) that interactions with faculty are more important for academic integration, whereas interactions with peers should matter mostly for social integration (see also Credé & Niehorster, 2012). However, Freeman et al. (2007) found similar cross‐sectional associations with university freshmen's academic motivation for support provided by professors and social acceptance by peers at the university level. It is possible that the relative importance of different sources of social support varies between contexts and groups of students. When looking at the whole sample, it seems that programs to increase confidence in getting support by faculty are more important to increase student motivation compared with peer programs. However, this is not the case when looking at subgroups such as FG and transfer students. We discuss this point below.

Notably, peer support was both positively linked with positive STVs (in Fall 2019) and with cost across cohorts. Although the positive associations with perceived cost are somewhat counterintuitive, it could be that students who perceive a specific course as stressful and time‐consuming are the ones who are more likely to anticipate needing social support. This perceived need for support from peers, in turn, might come with pressure and thus perceived costs (for costs of help‐seeking, see Karabenick & Berger, 2013). Interestingly, students' positive STVs and cost in both courses were positively (albeit weakly) associated. This positive association is not typical in SEVT‐related research in university contexts (e.g. Benden & Lauermann, 2022; Perez et al., 2019; Robinson et al., 2019). However, it is in line with Atkinson's (1957) original conceptualization of STV, who defined value as the inverse of the probability of success and thus expected individuals to have higher values for harder tasks requiring more effort. In our study, the positive association could arise from students' self‐identification of their most difficult/important courses. Therefore, students who perceived these courses as very valuable could also be willing to invest more effort while perceiving this required effort as stressful.

Overall, the longitudinal associations that we found between confidence in getting support and students' expectancies and STVs were small. However, this is probably to be expected when the initial levels of these constructs are controlled for, especially given the relatively high stabilities we observed in confidence in getting support and STVs. Prior research investigating similar longitudinal associations between confidence in getting support and academic motivation in school settings also reported small time‐lagged associations (e.g. Bostwick et al., 2022; Jang et al., 2012; Song et al., 2015). Moreover, whereas we assessed expectancies and STVs for specific courses, our confidence in getting support scales focused on faculty and peers at university more generally. Drawing on the specificity matching principle (Swann et al., 2007), more pronounced associations would be expected when confidence in getting support and academic motivation are measured at the same level of specificity versus generality. Indeed, Freeman et al. (2007) found that sense of belonging at the class level was more strongly related to students' concurrent course‐specific academic self‐efficacy, intrinsic motivation, and STV than sense of belonging at the university level. Therefore, it is important to note that we still found some evidence for longitudinal associations.

We tested the generalizability of the associations between social support and students' academic motivation across two cohorts and thus the robustness across in‐person and virtual learning settings. Although we found somewhat fewer significant cross‐lagged associations in the Fall 2020 data, the overall pattern and size of these cross‐lagged effects were similar across the data sets. Von Keyserlingk et al. (2024) had already shown that students in this sample perceived lower confidence in getting support from peers and faculty in the virtual setting during the COVID‐19 pandemic. Still, it seems that the links between social support and academic motivation are relatively robust to such differences in the learning context. Moreover, we investigated the robustness of the associations across two different courses. Again, the pattern of effects was relatively similar across these two courses, although not all effects could be found in both courses.

### The role of confidence in getting support for expectancies and STVs for FG and transfer students

We were particularly interested in FG and transfer students because these students face particular challenges at university. It is often assumed that these students are likely to perceive lower levels of social support and belonging at university than more privileged students. However, we found that FG students and transfer students reported the same levels of confidence in getting support as their peers. Prior research yielded mixed evidence regarding such differences, which vary likely by context and the extent to which these groups are integrated at specific institutions. Notably, prior studies reporting substantial differences were conducted at predominantly White universities with low ratios of FG students (Gillen‐O'Neel, 2021; Stebleton et al., 2014). In contrast, our study was conducted at a racially diverse institution where around half of the undergraduate students are FG students. Therefore, it seems likely that FG students are integrated socially to a greater extent in this context. The university also has a high ratio of transfer students (around 1/3 of all enrolments in Fall 2019 and around 1/5 of all enrolments in Fall 2020) and offers special resources and programs for these students.

Confidence in getting support tended to play a more significant role for FG and transfer students. This aligns with prior studies showing that student integration and sense of belonging predict academic development more strongly for FG students compared with CG students (Gillen‐O'Neel, 2021; Lohfink & Paulsen, 2005; Pascarella et al., 2004). Researchers have commonly assumed that FG students are more vulnerable to negative social experiences because of social identity threats and doubts about belonging to university (Murphy et al., 2020; Yeager et al., 2016). In addition, these students may need more support from faculty and peers to compensate for a lack of academic preparation and lower family support in academic matters (Jenkins et al., 2013; Saenz et al., 2007). These explanations might also be applicable to transfer students (Shaw & Chin‐Newman, 2017). In line with prior research, this study thus shows the role that social support can play for disadvantaged students. Universities should therefore provide specific programs, making sure to integrate these students and provide them with the necessary knowledge on how they can access support if needed. However, along with higher expectancies and positive STVs, confidence in getting support also predicted higher perceived costs for these students. It is possible that a perceived need for getting support by faculty or peers is perceived as effortful by these students (cf. Payne et al., 2023), similar to the points discussed for the whole sample above. Whereas our study investigated both faculty and peer support, we did not take support by parents into account. Research has found that parent support may also be of particular importance for FG students (Saenz et al., 2007; Sy et al., 2011). Thus, future research should consider parents as an additional source of support alongside faculty and peer support.

Interestingly, whereas we observed closer links with students' expectancies and positive STVs for confidence in getting faculty compared with peer support when considering all students, confidence in getting peer support was more strongly predictive of motivation for FG and transfer students. Thus, it seems that although these students are well‐integrated socially, they have a particularly strong need for building good academic relations with their peers in order to develop high expectancies and STVs. This may be because these students have doubts about their belonging at university and are more vulnerable to fluctuations in their sense of belonging (Chin‐Newman & Shaw, 2013; Gillen‐O'Neel, 2021; Murphy et al., 2020; Yeager et al., 2016) or because they have stronger interdependent norms and thus may rely on such support more strongly (e.g. Stephens et al., 2012). Asking peers for support might also be perceived as less challenging for these students compared with faculty, and peer programs therefore can be a valuable resource. When it comes to interventions for FG and transfer students in particular, universities should thus also aim to foster peer support, for instance, by increasing academic interactions between students. Such interventions could be an option to empower FG and transfer students. At the same time, interventions that address social support and academic motivation need to also consider the structural ways through which university contributes to inequalities (Smit, 2012).

### Limitations and future research

Although we used longitudinal data from a diverse sample, our study also has several limitations. First, although multiple time points were available for measures of social support and academic motivation, due to the items being embedded into a larger data collection and limitations in the length of the weekly surveys, the time points for the two constructs were not exactly parallel, and a third time point was missing for confidence in getting support. This limits the conclusions that can be drawn about the directionality of the longitudinal associations. Future research should therefore replicate our study with a fully matched longitudinal design. Moreover, we only examined within‐semester changes in confidence in getting support and academic motivation. Such changes can occur and were found to be important predictors of outcomes such as student engagement, exam performance, and dropout (e.g. Benden & Lauermann, 2022; Gillen‐O'Neel, 2021). Future research might also want to examine changes across semesters where stabilities in perceived support and academic motivation are likely lower than the ones found in our study.

Second, as already discussed above, confidence in getting support was measured at the university level, whereas academic motivation was assessed in specific courses. Therefore, it would be interesting to assess both confidence in getting support and academic motivation at the university and course levels to be able to investigate whether more pronounced associations are found when the level of specificity is the same.

Third, although our sample was diverse, it was not very large. Some of the subgroups (i.e. transfer students in particular) were relatively small, which led to relatively large confidence intervals in estimating longitudinal associations in these subgroups. We also simplified the model estimated in the multi‐group analyses, leaving out the timing of the SEVT measures for the second measurement time, which might have impacted the subgroup results if the timing systematically differed between subgroups. Sample size issues also refrained us from examining intersections between being a FG and a transfer student (see Supplemental Materials for the overlap between categories). Students who pertain to both of these categories might particularly benefit from social support, and future research might want to examine such intersections. Furthermore, we only had data available from a single university. In some sense, this is a strength because the university environment was the same for all students in the sample, and it allowed us to contextualize our findings, but it also constrains generalizability. Future research should aim to test the robustness of such longitudinal associations in larger samples from different contexts.

### Conclusions

Based on longitudinal data, our study showed some evidence that confidence in getting social support at university is reciprocally linked with students' course‐specific expectancies and STVs over time, although these effects were small. The reciprocal relations were similar across two different courses and also across regular and pandemic‐affected terms, which speaks to the generalizability across in‐person and virtual learning settings. There was no evidence for lower mean levels of confidence in getting social support for FG and transfer students. However, the longitudinal associations between students' confidence in getting support and their later motivation, found throughout the student population, tended to be stronger for FG and transfer students compared with their peers. Intervention research can build on our findings to examine whether programs to promote social support by faculty and peers indeed result in enhanced academic motivation. Such programs could be particularly beneficial for FG and transfer students, who have a higher risk of underperformance and dropout. However, some of the positive associations with perceived cost also suggest that confidence in getting support may not only have advantages for motivation but can also lead to an increase in perceptions of stress or having to give up other valued activities. Future research should aim to continue longitudinal research on the associations between social support and academic motivation in larger samples, across contexts, and over longer periods of time.

## AUTHOR CONTRIBUTIONS

**Hanna Gaspard:** Conceptualization; writing – original draft; writing – review and editing; visualization; formal analysis; methodology. **Cora Parrisius:** Conceptualization; formal analysis; visualization; writing – original draft; writing – review and editing. **Luise von Keyserlingk:** Conceptualization; writing – original draft; writing – review and editing; project administration; data curation. **Charlott Rubach:** Writing – review and editing; conceptualization; writing – original draft. **Katsumi Yamaguchi‐Pedroza:** Project administration; data curation; writing – review and editing; conceptualization. **Hye Rin Lee:** Conceptualization; writing – review and editing. **Marion Spengler:** Writing – review and editing; conceptualization. **Christian Fischer:** Conceptualization; writing – review and editing. **Jutta Heckhausen:** Writing – review and editing; conceptualization. **Jacquelynne S. Eccles:** Conceptualization; writing – review and editing.

## FUNDING INFORMATION

This research was supported by The Andrew W. Mellon Foundation (1806–05902).

## CONFLICT OF INTEREST STATEMENT

None.

## Supporting information


Appendix A.

## Data Availability

Data were collected as part of the UCI Measuring Undergraduate Success Trajectories Project, a large longitudinal project. It is planned to make the data collected by this larger research project openly available at a later time point.

## Footnotes

^1^
Data from this larger project has been used for other publications that partly overlap in the data being used, albeit their focus was on clearly different research questions. Von Keyserlingk et al. (2024) investigated the role of ability beliefs and confidence in getting support for positive academic and personal‐emotional adjustment before and during the pandemic. Furthermore, von Keyserlingk et al. (2022) examined how expectancies, subjective task values, and experiences in a difficult course predict the use of goal‐oriented control strategies.

^2^
In Fall 2019, students were asked to identify their most difficult and most important course at each individual time point without any constraints for these nominations in terms of overlapping courses or changes across time. In consequence, the most difficult course was also the most important course for 95 students (30%) at T1. Moreover, students' most difficult and most important course could change across the term: at T2, the most difficult course had changed compared to T1 for 64 students (20%) and the most important course had changed for 19 students (6%). At T3, the most difficult course had changed for 90 students (28%, missing information for 37%) and the most important course had changed for 85 students (27%, missing information for 25%). However, given that confidence in getting support was assessed at a general level, longitudinal effects across courses are still plausible. A robustness analysis with only those students whose most difficult/most important course did not change across the term showed no substantial differences in the findings compared to the total sample reported here (see Supplemental Materials). In Fall 2020, students were not allowed to change the courses they reported about across the term. Moreover, if their most important course was the same as their most difficult one, they were asked to report their expectancies and STVs in their second most important course.

^3^
To formally compare the stability and cross‐lagged coefficients in the Fall 2019 and Fall 2020 data, we conducted multi‐group analyses (see Supplemental Materials for the full results). We found no statistically significant differences for the stability coefficients for confidence in getting support (*p* ≥ .635). With respect to the stability coefficients of motivational beliefs, 11 out of 36 coefficients were statistically significantly different (*p* < .05). Most of these significant differences indicated higher stabilities in the Fall 2020 data for motivational beliefs from T2 to T3. These higher stabilities might be because students were not able to change their most difficult and most important course in this data collection. However, only two out of the 48 cross‐lagged coefficients showed significant differences (*p* < .05) between the two cohorts. The path from faculty support at T1 on expectancy in students' most important course at T3, which was positive and significant only in the Fall 2019 data, was significantly higher (*p* = .04) in the Fall 2019 data compared with the Fall 2020 data. Moreover, the path from peer support at T1 on cost in students' most difficult course at T3, which was positive and significant only in the Fall 2019 data, differed statistically significant from the path in the Fall 2020 data (*p* = .004), where it tended to be negative.
