# Supplementary material for: Reciprocal associations between confidence in getting social support and academic expectancies and subjective task values: Stronger for first‐generation and transfer students
Source: Br J Educ Psychol. 2025 Feb 26;95(4):1023–46. doi: 10.1111/bjep.12751 (PMC12590933; doi:10.1111/bjep.12751)
Supplement: Supplementary file 1 — Appendix A. [file BJEP-95-1023-s001.docx]

**Supplemental Materials to**

**Gaspard, H., Parrisius, C., von Keyserlingk, L., Rubach, C., Yamaguchi-Pedroza, K., Lee, H. R., Spengler, M., Fischer, C., Heckhausen, J., & Eccles, J. S.**

**Reciprocal Associations Between Confidence in Getting Social Support and Academic Expectancies and Subjective Task Values: Stronger for First-Generation and Transfer Students**

Table of Contents

Appendix A: Additional Information on Demographic Characteristics of the Sample

Appendix B: Instruments

Appendix C: Confirmatory Factor Analyses and Measurement Invariance Tests

Appendix D: Descriptive Statistics

Appendix E: Analyses For Those Students Whose Most Difficult/Most Important Course Remained the Same Across Time in the Fall 2019 Data

Appendix F: Missing Data Analyses

Appendix G: Tables with Covariates (Full Sample)

Appendix H: Differences in Stability and Cross-lagged Coefficients Between the Fall 2019 and Fall 2020 Data

Appendix I: Tables for Multi-Group Analyses

**Appendix A**

**Additional Information on Demographic Characteristics of the Sample**

**Table A1**

*Overlap Between First-Generation and Transfer Students*

|  | Fall 2019 | | Fall 2020 | |
| --- | --- | --- | --- | --- |
| College generation | Non-transfer student | Transfer student | Non-transfer student | Transfer student |
| CG student | 106 | 30 | 122 | 49 |
| FG student | 147 | 33 | 181 | 43 |

**Table A2**

*Student Characteristics of Study Participants and the Overall Undergraduate Student Population at the University*

|  | Fall 2019 | | Fall 2020 | |
| --- | --- | --- | --- | --- |
| Variable | Study sample (%) | Undergraduate student population (%) | Study sample (%) | Undergraduate student population (%) |
| Female | 68.4 | 51.8 | 71.7 | 52.4 |
| FG student | 56.3 | 46.7 | 53.7 | 47.1 |
| URM student | 37.8 | 29.8 | 36.0 | 29.1 |

**Appendix B**

**Instruments**

Confidence in Getting Support:

The items for assessing confidence in getting support by peers and faculty were mostly taken from a well-established scale measuring social support in university (Hoffman et al., n.d.). They were adapted to avoid agree-disagree statements and to instead label response anchors with construct-specific labels (Gehlbach, 2015; Saris et al., 2010). Feeling comfortable and confident one can receive support is an important source of students’ sense of belonging (Hoffman et al., n.d.), so these perceptions were used for the response anchors. Students rated their confidence in receiving support from peers on a slider from 0 = *not at all confident* to 100 = *extremely confident*. As we deemed academic support by peers to be more important for students’ academic motivation compared to emotional support (cf. Wentzel et al., 2010), we used three items referring to academic support for our analyses. To indicate faculty support, students rated their perceived comfort or confidence to get support from faculty on three items each on a slider from 0 = *not at all comfortable/confident* to 100 = *extremely comfortable/confident*. Both scales showed good internal consistencies across time and cohorts. Confirmatory factor analyses, in which residual correlations were allowed between faculty items that used the same response anchors (i.e., comfortable vs. confident), provided support for the separability of the two support dimensions at both time points in the Fall 2019 and Fall 2020 data (see Table B1). Furthermore, we found support for strict measurement invariance across time and subgroups, relying on the recommendations for changes in model fit indices provided by Chen (2007) and Cheung and Rensvold (2002; see Tables B2-B4).

*Peer support:*

How confident are you that you could:

- Call another student if you had a question about an assignment?
- Count on other students to be helpful in reminding you when assignments are due or when tests are approaching?
- Get the notes from other student(s) if you miss class

*Faculty support:*

How comfortable would you feel if you had to talk about an academic problem with faculty?

How comfortable would you feel if you had to talk about a personal problem with faculty?

How comfortable would you feel if you had to seek help from faculty outside of class?

How confident are you that faculty would take the time to talk to you if you needed help?

How confident are you that faculty would be sympathetic if you were upset?

How confident are you that faculty would be sensitive to difficulties if you shared them?

Expectancies and subjective task values

Expectancies and all STV value components (i.e., intrinsic, attainment, utility, and cost) were measured with two items each based on SEVT (Eccles & Wigfield, 2020). Only two items were used because of the study design involving weekly surveys (for a similar approach, see Benden & Lauermann, 2022; Beymer, Benden, et al., 2022; for the validity of short scales, see Beymer, Ferland, et al., 2022; Gogol et al., 2014).

In Fall 2019, expectancy and STVs items measured during T1 and T3 were on a 7-point Likert scale ranging from 1 (*not at all good*) to 7 (*extremely good*) for expectancy items, from 1 (*not at all expected*) to 7 (*very much expected*) for STV items at T1 and from 1 (*not at all*) to 7 (*very much*) for STV items at T3. Expectancy and STV items measured during T2 were on a 100-point slider from 0 (*not at all good*) to 100 (*extremely good*) for expectancy items and from 0 (*not at all*) to 100 (*very much*) for STV items but transformed to a 1 to 7 scale using linear interpolation to have a consistent metric across time. In Fall 2020, expectancy and STV items at all time points relied on a 7-point Likert scale. Construct-specific labels as response anchors were used in line with recommendations in the survey literature (Gehlbach, 2015; Saris et al., 2010). The expectancy scale showed high internal consistencies across cohorts, courses, and time points. For this study, we combined the intrinsic, attainment, and utility items into one scale assessing positive values because of their high intercorrelations in preliminary factor analyses (ρ = .67 to 1.00; for a similar approach, see Flake et al., 2015; Kryshko et al., 2022; Perez et al., 2014). The resulting scale had a good internal consistency across cohorts, courses, and time. The two cost items were used as a separate scale, which showed sufficient consistency across cohorts, courses, and time.

Confirmatory factor analyses, in which STV items indicating intrinsic, attainment, and utility values were set to load onto one latent factor with residual correlations between the items tapping the same component, provided support for the separability of the expectancy, STV, and cost scales across courses and time points in Fall 2019 and Fall 2020 data (see Table B5 and B9). Furthermore, we tested measurement invariance across time and subgroups, relying on the recommendations for changes in model fit indices provided by Chen (2007) and Cheung and Rensvold (2002; see Tables B6 to B8 and B10 to B12). Across time, we observed decreases in CFI larger than .01 when testing strong invariance (in both cohorts and across courses). Although this may indicate a lack of invariance in the item intercepts across time for these scales, the RMSEA and the SRMR did not decrease substantially, and the fit of the resulting models was still acceptable. Therefore, strong (and even strict) invariance across time is still defensible in our view. Moreover, we note that we did not compare the mean levels of these constructs. For tests across groups, with few exceptions, no substantial decreases in model fit occurred when imposing invariance constraints and the fit of the final models met recommendations for acceptable model fit (Hu & Bentler, 1999; Marsh et al., 2004) or was only slightly lower.

*Expectancies:*

How good do you think you will be learning the new material in X?

Compared to other subject areas, how good are you at learning things in X?

*Positive subjective task values:*

How much do you expect that X will:

- Be useful in everyday life?
- Be interesting to you?
- Be important to your identity to be knowledgeable in this class?
- Be intellectually challenging in a positive way?
- Important to you personally in terms of your values and identities?
- Be useful in terms of your long-term goals?

*Cost:*

How much do you expect that X will:

- Be stressful?
- Require you to give up on other valued activities?

**Appendix C**

**Confirmatory Factor Analyses and Measurement Invariance Tests**

**Table C1**

*Confirmatory Factor Analyses for Social Support Scales at Each Time Point*

| Model | χ² | df | CFI | RMSEA | SRMR |
| --- | --- | --- | --- | --- | --- |
| *Fall 2019* | | | | | |
| T1 | 49.00 | 20 | .973 | .070 | .032 |
| T2 | 48.74 | 20 | .977 | .070 | .028 |
| *Fall 2020* | | | | | |
| T1 | 36.87 | 20 | .988 | .048 | .031 |
| T2 | 33.94 | 20 | .983 | .044 | .024 |

*Note.* Separate factors were modeled for faculty and peer support. Residual correlations were allowed between faculty items that used the same response anchors (i.e., comfortable vs. confident). The two factors showed correlations between ρ = .44 and ρ = .54.

**Table C2**

*Tests of Measurement Invariance for Social Support Scales Across Time*

| Model | χ² | df | SCF | CFI | RMSEA | SRMR | Model  comparison | Δχ² | Δdf | *p* | ΔCFI | ΔRMSEA | ΔSRMR |
| --- | --- | --- | --- | --- | --- | --- | --- | --- | --- | --- | --- | --- | --- |
| *Fall 2019:* | | | | | | | | | | | | | |
| M1: configural invariance | 158.39 | 96 | 1.19 | 0.977 | 0.046 | 0.033 |  |  |  |  |  |  |  |
| M2: weak invariance | 170.27 | 103 | 1.17 | 0.976 | 0.046 | 0.038 | M2 vs. M1 | 11.95 | 7 | .102 | -0.001 | 0.000 | 0.005 |
| M3: strong invariance | 178.53 | 110 | 1.16 | 0.975 | 0.045 | 0.039 | M3 vs. M2 | 7.86 | 7 | .345 | -0.001 | -0.001 | 0.001 |
| M4: strict invariance | 184.49 | 119 | 1.16 | 0.976 | 0.042 | 0.041 | M4 vs. M3 | 5.30 | 9 | .807 | 0.001 | -0.003 | 0.002 |
| *Fall 2020:* | | | | | | | | | | | | | |
| M1: configural invariance | 117.07 | 96 | 1.18 | 0.994 | 0.024 | 0.033 |  |  |  |  |  |  |  |
| M2: weak invariance | 122.93 | 103 | 1.16 | 0.994 | 0.022 | 0.034 | M2 vs. M1 | 5.18 | 7 | .638 | 0.000 | -0.002 | 0.001 |
| M3: strong invariance | 132.79 | 110 | 1.15 | 0.994 | 0.023 | 0.035 | M3 vs. M2 | 10.10 | 7 | .183 | 0.000 | 0.001 | 0.001 |
| M4: strict invariance | 151.06 | 119 | 1.14 | 0.991 | 0.026 | 0.039 | M4 vs. M3 | 19.11 | 9 | .024 | -0.003 | 0.003 | 0.004 |

**Table C3**

*Tests of Measurement Invariance for Social Support Scales Across Groups: FG and CG Students*

| Model | χ² | *df* | SCF | CFI | RMSEA | SRMR | Model  comparison | Δχ² | Δdf | *p* | ΔCFI | ΔRMSEA | ΔSRMR |
| --- | --- | --- | --- | --- | --- | --- | --- | --- | --- | --- | --- | --- | --- |
| *Fall 2019* | | | | | | | | | | | | | |
| T1 |  |  |  |  |  |  |  |  |  |  |  |  |  |
| M1: configural invariance | 77.16 | 40 | 1.19 | 0.965 | 0.079 | 0.040 |  |  |  |  |  |  |  |
| M2: weak invariance | 85.51 | 47 | 1.16 | 0.964 | 0.074 | 0.050 | M2 vs. M1 | 7.69 | 7 | .360 | -0.001 | -0.005 | 0.010 |
| M3: strong invariance | 90.22 | 54 | 1.14 | 0.966 | 0.067 | 0.051 | M3 vs. M2 | 3.53 | 7 | .832 | 0.002 | -0.007 | 0.001 |
| M4: strict invariance | 94.24 | 63 | 1.15 | 0.971 | 0.057 | 0.055 | M4 vs. M3 | 4.31 | 9 | .890 | 0.005 | -0.010 | 0.004 |
| T2 |  |  |  |  |  |  |  |  |  |  |  |  |  |
| M1: configural invariance | 93.94 | 40 | 0.99 | 0.960 | 0.097 | 0.035 |  |  |  |  |  |  |  |
| M2: weak invariance | 102.87 | 47 | 1.00 | 0.958 | 0.091 | 0.048 | M2 vs. M1 | 9.48 | 7 | .220 | -0.002 | -0.006 | 0.013 |
| M3: strong invariance | 107.00 | 54 | 1.00 | 0.960 | 0.082 | 0.049 | M3 vs. M2 | 4.01 | 7 | .779 | 0.002 | -0.009 | 0.001 |
| M4: strict invariance | 120.90 | 63 | 1.04 | 0.957 | 0.080 | 0.058 | M4 vs. M3 | 14.54 | 9 | .104 | -0.003 | -0.002 | 0.009 |
| *Fall 2020* | | | | | | | | | | | | | |
| T1 |  |  |  |  |  |  |  |  |  |  |  |  |  |
| M1: configural invariance | 53.67 | 40 | 1.15 | 0.990 | 0.044 | 0.040 |  |  |  |  |  |  |  |
| M2: weak invariance | 64.07 | 47 | 1.12 | 0.988 | 0.045 | 0.046 | M2 vs. M1 | 10.61 | 7 | .157 | -0.002 | 0.001 | 0.006 |
| M3: strong invariance | 74.76 | 54 | 1.10 | 0.985 | 0.046 | 0.049 | M3 vs. M2 | 10.82 | 7 | .147 | -0.003 | 0.001 | 0.003 |
| M4: strict invariance | 86.05 | 63 | 1.08 | 0.983 | 0.045 | 0.048 | M4 vs. M3 | 11.16 | 9 | .265 | -0.002 | -0.001 | -0.001 |
| T2 |  |  |  |  |  |  |  |  |  |  |  |  |  |
| M1: configural invariance | 61.68 | 40 | 1.13 | 0.985 | 0.056 | 0.036 |  |  |  |  |  |  |  |
| M2: weak invariance | 69.41 | 47 | 1.11 | 0.985 | 0.053 | 0.043 | M2 vs. M1 | 7.46 | 7 | .383 | 0.000 | -0.003 | 0.007 |
| M3: strong invariance | 74.71 | 54 | 1.10 | 0.986 | 0.047 | 0.044 | M3 vs. M2 | 4.94 | 7 | .668 | 0.001 | -0.006 | 0.001 |
| M4: strict invariance | 79.97 | 63 | 1.11 | 0.989 | 0.040 | 0.043 | M4 vs. M3 | 5.60 | 9 | .779 | 0.003 | -0.007 | -0.001 |

**Table C4**

*Tests of Measurement Invariance for Social Support Scales Across Groups: Transfer and Non-transfer Students*

| Model | χ² | *df* | SCF | CFI | RMSEA | SRMR | Model  comparison | Δχ² | Δdf | *p* | ΔCFI | ΔRMSEA | ΔSRMR |
| --- | --- | --- | --- | --- | --- | --- | --- | --- | --- | --- | --- | --- | --- |
| *Fall 2019* | | | | | | | | | | | | | |
| T1 |  |  |  |  |  |  |  |  |  |  |  |  |  |
| M1: configural invariance | 71.46 | 40 | 1.17 | 0.971 | 0.072 | 0.039 |  |  |  |  |  |  |  |
| M2: weak invariance | 78.54 | 47 | 1.16 | 0.971 | 0.066 | 0.047 | M2 vs. M1 | 6.64 | 7 | .467 | 0.000 | -0.006 | 0.008 |
| M3: strong invariance | 89.24 | 54 | 1.11 | 0.968 | 0.065 | 0.051 | M3 vs. M2 | 10.36 | 7 | .169 | -0.003 | -0.001 | 0.004 |
| M4: strict invariance | 95.04 | 63 | 1.11 | 0.971 | 0.058 | 0.054 | M4 vs. M3 | 5.81 | 9 | .759 | 0.003 | -0.007 | 0.003 |
| T2 |  |  |  |  |  |  |  |  |  |  |  |  |  |
| M1: configural invariance | 82.04 | 40 | 0.98 | 0.969 | 0.085 | 0.035 |  |  |  |  |  |  |  |
| M2: weak invariance | 83.90 | 47 | 0.99 | 0.973 | 0.073 | 0.037 | M2 vs. M1 | 2.82 | 7 | .901 | 0.004 | -0.012 | 0.002 |
| M3: strong invariance | 93.93 | 54 | 0.99 | 0.971 | 0.071 | 0.039 | M3 vs. M2 | 10.03 | 7 | .187 | -0.002 | -0.002 | 0.002 |
| M4: strict invariance | 96.66 | 63 | 1.00 | 0.975 | 0.060 | 0.045 | M4 vs. M3 | 3.38 | 9 | .947 | 0.004 | -0.011 | 0.006 |
| *Fall 2020* | | | | | | | | | | | | | |
| T1 |  |  |  |  |  |  |  |  |  |  |  |  |  |
| M1: configural invariance | 74.09 | 40 | 1.09 | 0.977 | 0.068 | 0.038 |  |  |  |  |  |  |  |
| M2: weak invariance | 82.30 | 47 | 1.07 | 0.976 | 0.063 | 0.045 | M2 vs. M1 | 7.62 | 7 | .367 | -0.001 | -0.005 | 0.007 |
| M3: strong invariance | 94.51 | 54 | 1.06 | 0.973 | 0.063 | 0.049 | M3 vs. M2 | 12.20 | 7 | .094 | -0.003 | 0.000 | 0.004 |
| M4: strict invariance | 104.15 | 63 | 1.07 | 0.972 | 0.059 | 0.052 | M4 vs. M3 | 10.02 | 9 | .349 | -0.001 | -0.004 | 0.003 |
| T2 |  |  |  |  |  |  |  |  |  |  |  |  |  |
| M1: configural invariance | 63.06 | 40 | 1.09 | 0.985 | 0.057 | 0.030 |  |  |  |  |  |  |  |
| M2: weak invariance | 73.42 | 47 | 1.08 | 0.983 | 0.056 | 0.043 | M2 vs. M1 | 10.34 | 7 | .170 | -0.002 | -0.001 | 0.013 |
| M3: strong invariance | 87.33 | 54 | 1.06 | 0.979 | 0.059 | 0.047 | M3 vs. M2 | 14.40 | 7 | .044 | -0.004 | 0.003 | 0.004 |
| M4: strict invariance | 96.25 | 63 | 1.10 | 0.979 | 0.054 | 0.043 | M4 vs. M3 | 9.85 | 9 | .363 | 0.000 | -0.005 | -0.004 |

**Table C5**

*Confirmatory Factor Analyses for SEVT Scales in the Most Difficult Course at Each Time Point*

| Model | χ² | df | CFI | RMSEA | SRMR |
| --- | --- | --- | --- | --- | --- |
| *Fall 2019* | | | | | |
| T1 | 116.18 | 29 | .912 | .099 | .061 |
| T2 | 48.62 | 29 | .981 | .049 | .036 |
|  | 89.59 | 29 | .938 | .087 | .056 |
| *Fall 2020* | | | | | |
| T1 | 98.69 | 29 | .940 | .078 | .043 |
| T2 | 128.81 | 29 | .926 | .097 | .061 |
|  | 111.04 | 29 | .938 | .095 | .055 |

*Note.* Separate factors were modeled for expectancy, STV, and cost. Residual correlations were allowed between STV items indicating the same component (i.e., intrinsic, attainment, and utility value).

**Table C6**

*Tests of Measurement Invariance for SEVT Scales in the Most Difficult Course Across Time*

| Model | χ² | df | SCF | CFI | RMSEA | SRMR | Model  comparison | Δχ² | Δdf | *p* | ΔCFI | ΔRMSEA | ΔSRMR |
| --- | --- | --- | --- | --- | --- | --- | --- | --- | --- | --- | --- | --- | --- |
| *Fall 2019:* | | | | | | | | | | | | | |
| M1: configural invariance | 571.29 | 333 | 1.07 | 0.940 | 0.047 | 0.051 |  |  |  |  |  |  |  |
| M2: weak invariance | 587.76 | 347 | 1.07 | 0.940 | 0.047 | 0.054 | M2 vs. M1 | 16.31 | 14 | .295 | 0.000 | 0.000 | 0.003 |
| M3: strong invariance | 671.04 | 361 | 1.07 | 0.922 | 0.052 | 0.056 | M3 vs. M2 | 91.27 | 14 | <.001 | -0.018 | 0.005 | 0.002 |
| M4: strict invariance | 711.13 | 381 | 1.07 | 0.917 | 0.052 | 0.060 | M4 vs. M3 | 39.81 | 20 | .005 | -0.005 | 0.000 | 0.004 |
| *Fall 2020:* | | | | | | | | | | | | | |
| M1: configural invariance | 709.89 | 333 | 1.09 | 0.934 | 0.052 | 0.062 |  |  |  |  |  |  |  |
| M2: weak invariance | 727.54 | 347 | 1.09 | 0.933 | 0.051 | 0.063 | M2 vs. M1 | 16.95 | 14 | .259 | -0.001 | -0.001 | 0.001 |
| M3: strong invariance | 830.25 | 361 | 1.09 | 0.918 | 0.056 | 0.064 | M3 vs. M2 | 111.30 | 14 | <.001 | -0.015 | 0.005 | 0.001 |
| M4: strict invariance | 865.29 | 381 | 1.09 | 0.915 | 0.055 | 0.070 | M4 vs. M3 | 35.74 | 20 | .016 | -0.003 | -0.001 | 0.006 |

*Note*. Residual correlations were allowed for items indicating the same value component (i.e., intrinsic value, attainment value, and utility value) and for the same items across time.

**Table C7**

*Tests of Measurement Invariance for SEVT Scales in the Most Difficult Course Across Groups for FG and CG Students*

| Model | χ² | *df* | SCF | CFI | RMSEA | SRMR | Model  comparison | Δχ² | Δdf | *p* | ΔCFI | ΔRMSEA | ΔSRMR |
| --- | --- | --- | --- | --- | --- | --- | --- | --- | --- | --- | --- | --- | --- |
| *Fall 2019* | | | | | | | | | | | | | |
| T1 |  |  |  |  |  |  |  |  |  |  |  |  |  |
| M1: configural invariance | 162.35 | 58 | 1.05 | 0.897 | 0.108 | 0.071 |  |  |  |  |  |  |  |
| M2: weak invariance | 163.67 | 65 | 1.06 | 0.903 | 0.100 | 0.073 | M2 vs. M1 | 1.89 | 7 | .966 | 0.006 | -0.008 | 0.002 |
| M3: strong invariance | 169.48 | 72 | 1.05 | 0.904 | 0.094 | 0.073 | M3 vs. M2 | 5.22 | 7 | .634 | 0.001 | -0.006 | 0.000 |
| M4: strict invariance | 180.99 | 82 | 1.06 | 0.902 | 0.089 | 0.083 | M4 vs. M3 | 12.14 | 10 | .276 | -0.002 | -0.005 | 0.010 |
| T2 |  |  |  |  |  |  |  |  |  |  |  |  |  |
| M1: configural invariance | 78.38 | 58 | 1.16 | 0.979 | 0.052 | 0.046 |  |  |  |  |  |  |  |
| M2: weak invariance | 86.07 | 65 | 1.15 | 0.979 | 0.050 | 0.055 | M2 vs. M1 | 7.56 | 7 | .373 | 0.000 | -0.002 | 0.009 |
| M3: strong invariance | 96.18 | 72 | 1.13 | 0.975 | 0.051 | 0.056 | M3 vs. M2 | 10.23 | 7 | .176 | -0.004 | 0.001 | 0.001 |
| M4: strict invariance | 106.05 | 82 | 1.14 | 0.976 | 0.047 | 0.065 | M4 vs. M3 | 9.99 | 10 | .442 | 0.001 | -0.004 | 0.009 |
| T3 |  |  |  |  |  |  |  |  |  |  |  |  |  |
| M1: configural invariance | 141.31 | 58 | 1.09 | 0.920 | 0.103 | 0.066 |  |  |  |  |  |  |  |
| M2: weak invariance | 163.31 | 65 | 1.07 | 0.905 | 0.105 | 0.077 | M2 vs. M1 | 23.14 | 7 | .002 | -0.015 | 0.002 | 0.011 |
| M3: strong invariance | 179.47 | 72 | 1.05 | 0.896 | 0.105 | 0.079 | M3 vs. M2 | 16.00 | 7 | .025 | -0.009 | 0.000 | 0.002 |
| M4: strict invariance | 187.66 | 82 | 1.08 | 0.898 | 0.097 | 0.076 | M4 vs. M3 | 10.46 | 10 | .401 | 0.002 | -0.008 | -0.003 |
| *Fall 2020* | | | | | | | | | | | | | |
| T1 |  |  |  |  |  |  |  |  |  |  |  |  |  |
| M1: configural invariance | 130.25 | 58 | 1.13 | 0.937 | 0.082 | 0.049 |  |  |  |  |  |  |  |
| M2: weak invariance | 131.83 | 65 | 1.15 | 0.942 | 0.074 | 0.054 | M2 vs. M1 | 2.79 | 7 | .904 | 0.005 | -0.008 | 0.005 |
| M3: strong invariance | 141.98 | 72 | 1.13 | 0.939 | 0.072 | 0.055 | M3 vs. M2 | 9.47 | 7 | .220 | -0.003 | -0.002 | 0.001 |
| M4: strict invariance | 162.18 | 82 | 1.13 | 0.930 | 0.072 | 0.073 | M4 vs. M3 | 20.21 | 10 | .027 | -0.009 | 0.000 | 0.018 |
| T2 |  |  |  |  |  |  |  |  |  |  |  |  |  |
| M1: configural invariance | 180.32 | 58 | 1.13 | 0.910 | 0.110 | 0.069 |  |  |  |  |  |  |  |
| M2: weak invariance | 186.70 | 65 | 1.15 | 0.910 | 0.104 | 0.074 | M2 vs. M1 | 8.25 | 7 | .311 | 0.000 | -0.006 | 0.005 |
| M3: strong invariance | 194.32 | 72 | 1.13 | 0.910 | 0.099 | 0.076 | M3 vs. M2 | 5.74 | 7 | .570 | 0.000 | -0.005 | 0.002 |
| M4: strict invariance | 200.00 | 82 | 1.14 | 0.913 | 0.091 | 0.079 | M4 vs. M3 | 7.17 | 10 | .709 | 0.003 | -0.008 | 0.003 |
| T3 |  |  |  |  |  |  |  |  |  |  |  |  |  |
| M1: configural invariance | 132.94 | 58 | 1.16 | 0.942 | 0.093 | 0.058 |  |  |  |  |  |  |  |
| M2: weak invariance | 144.90 | 65 | 1.14 | 0.938 | 0.091 | 0.067 | M2 vs. M1 | 11.29 | 7 | .127 | -0.004 | -0.002 | 0.009 |
| M3: strong invariance | 158.17 | 72 | 1.12 | 0.933 | 0.090 | 0.067 | M3 vs. M2 | 12.93 | 7 | .074 | -0.005 | -0.001 | 0.000 |
| M4: strict invariance | 170.18 | 82 | 1.13 | 0.932 | 0.085 | 0.073 | M4 vs. M3 | 12.56 | 10 | .249 | -0.001 | -0.005 | 0.006 |

**Table C8**

*Tests of Measurement Invariance for SEVT Scales in the Most Difficult Course Across Groups for Transfer and Non-transfer Students*

| Model | χ² | *df* | SCF | CFI | RMSEA | SRMR | Model  comparison | Δχ² | Δdf | *p* | ΔCFI | ΔRMSEA | ΔSRMR |
| --- | --- | --- | --- | --- | --- | --- | --- | --- | --- | --- | --- | --- | --- |
| *Fall 2019* | | | | | | | | | | | | | |
| T1 |  |  |  |  |  |  |  |  |  |  |  |  |  |
| M1: configural invariance | 171.24 | 58 | 1.00 | 0.885 | 0.112 | 0.067 |  |  |  |  |  |  |  |
| M2: weak invariance | 173.36 | 65 | 1.05 | 0.890 | 0.104 | 0.073 | M2 vs. M1 | 6.81 | 7 | .449 | 0.005 | -0.008 | 0.006 |
| M3: strong invariance | 180.17 | 72 | 1.05 | 0.890 | 0.099 | 0.075 | M3 vs. M2 | 6.84 | 7 | .446 | 0.000 | -0.005 | 0.002 |
| M4: strict invariance | 188.63 | 82 | 1.07 | 0.891 | 0.092 | 0.093 | M4 vs. M3 | 10.30 | 10 | .414 | 0.001 | -0.007 | 0.018 |
| T2 |  |  |  |  |  |  |  |  |  |  |  |  |  |
| M1: configural invariance | 101.39 | 58 | 1.09 | 0.960 | 0.075 | 0.049 |  |  |  |  |  |  |  |
| M2: weak invariance | 109.71 | 65 | 1.09 | 0.959 | 0.072 | 0.061 | M2 vs. M1 | 8.25 | 7 | .311 | -0.001 | -0.003 | 0.012 |
| M3: strong invariance | 121.67 | 72 | 1.08 | 0.955 | 0.072 | 0.065 | M3 vs. M2 | 11.97 | 7 | .101 | -0.004 | 0.000 | 0.004 |
| M4: strict invariance | 149.28 | 82 | 1.10 | 0.939 | 0.079 | 0.073 | M4 vs. M3 | 26.06 | 10 | .004 | -0.016 | 0.007 | 0.008 |
| T3 |  |  |  |  |  |  |  |  |  |  |  |  |  |
| M1: configural invariance^a^ | 137.57 | 58 | 1.08 | 0.924 | 0.100 | 0.070 |  |  |  |  |  |  |  |
| M2: weak invariance | 141.98 | 65 | 1.07 | 0.926 | 0.093 | 0.073 | M2 vs. M1 | 3.87 | 7 | .795 | 0.002 | -0.007 | 0.003 |
| M3: strong invariance | 156.96 | 72 | 1.06 | 0.919 | 0.092 | 0.077 | M3 vs. M2 | 14.93 | 7 | .037 | -0.007 | -0.001 | 0.004 |
| M4: strict invariance | 177.73 | 82 | 1.05 | 0.908 | 0.092 | 0.084 | M4 vs. M3 | 20.72 | 10 | .023 | -0.011 | 0.000 | 0.007 |
| *Fall 2020* | | | | | | | | | | | | | |
| T1 |  |  |  |  |  |  |  |  |  |  |  |  |  |
| M1: configural invariance | 157.09 | 58 | 1.12 | 0.920 | 0.094 | 0.052 |  |  |  |  |  |  |  |
| M2: weak invariance | 167.37 | 65 | 1.11 | 0.917 | 0.090 | 0.061 | M2 vs. M1 | 9.57 | 7 | .214 | -0.003 | -0.004 | 0.009 |
| M3: strong invariance | 185.20 | 72 | 1.11 | 0.908 | 0.090 | 0.066 | M3 vs. M2 | 17.82 | 7 | .013 | -0.009 | 0.000 | 0.005 |
| M4: strict invariance | 209.14 | 82 | 1.04 | 0.897 | 0.089 | 0.076 | M4 vs. M3 | 22.35 | 10 | .013 | -0.011 | -0.001 | 0.010 |
| T2 |  |  |  |  |  |  |  |  |  |  |  |  |  |
| M1: configural invariance | 162.95 | 58 | 1.13 | 0.927 | 0.100 | 0.064 |  |  |  |  |  |  |  |
| M2: weak invariance | 177.86 | 65 | 1.12 | 0.922 | 0.098 | 0.074 | M2 vs. M1 | 14.82 | 7 | .038 | -0.005 | -0.002 | 0.010 |
| M3: strong invariance | 189.11 | 72 | 1.12 | 0.919 | 0.094 | 0.077 | M3 vs. M2 | 10.79 | 7 | .148 | -0.003 | -0.004 | 0.003 |
| M4: strict invariance | 202.22 | 82 | 1.13 | 0.917 | 0.090 | 0.073 | M4 vs. M3 | 13.85 | 10 | .180 | -0.002 | -0.004 | -0.004 |
| T3 |  |  |  |  |  |  |  |  |  |  |  |  |  |
| M1: configural invariance | 139.81 | 58 | 1.13 | 0.939 | 0.095 | 0.062 |  |  |  |  |  |  |  |
| M2: weak invariance | 149.95 | 65 | 1.13 | 0.937 | 0.091 | 0.069 | M2 vs. M1 | 9.99 | 7 | .189 | -0.002 | -0.004 | 0.007 |
| M3: strong invariance | 162.80 | 72 | 1.12 | 0.932 | 0.090 | 0.076 | M3 vs. M2 | 12.71 | 7 | .080 | -0.005 | -0.001 | 0.007 |
| M4: strict invariance | 169.05 | 82 | 1.15 | 0.935 | 0.082 | 0.075 | M4 vs. M3 | 8.52 | 10 | .579 | 0.003 | -0.008 | -0.001 |

*Note.* ^a^This model only converged if we fixed one residual variance for transfer students to a positive value. This issue of non-convergence was most likely due to the small number of transfer students in the sample in comparison to the number of parameters in this model. The more parsimonious models did not lead to convergence problems.

**Table C9**

*Confirmatory Factor Analyses for SEVT Scales in the Most Important Course at Each Time Point*

| Model | χ² | df | CFI | RMSEA | SRMR |
| --- | --- | --- | --- | --- | --- |
| *Fall 2019* | | | | | |
| T1 | 95.68 | 29 | .926 | .086 | .057 |
| T2 | 27.41 | 29 | 1.000 | .000 | .034 |
|  | 69.94 | 29 | .955 | .072 | .054 |
| *Fall 2020* | | | | | |
| T1 | 96.50 | 29 | .945 | .077 | .049 |
| T2 | 91.60 | 29 | .960 | .077 | .045 |
|  | 112.85 | 29 | .936 | .078 | .045 |

*Note.* Separate factors were modeled for expectancy, STV, and cost. Residual correlations were allowed between STV items indicating the same component (i.e., intrinsic, attainment, and utility value).

**Table C10**

*Tests of Measurement Invariance for SEVT Scales in the Most Important Course Across Time*

| Model | χ² | df | SCF | CFI | RMSEA | SRMR | Model  comparison | Δχ² | Δdf | *p* | ΔCFI | ΔRMSEA | ΔSRMR |
| --- | --- | --- | --- | --- | --- | --- | --- | --- | --- | --- | --- | --- | --- |
| *Fall 2019:* | | | | | | | | | | | | | |
| M1: configural invariance | 514.54 | 333 | 1.11 | 0.952 | 0.041 | 0.052 |  |  |  |  |  |  |  |
| M2: weak invariance | 523.45 | 347 | 1.11 | 0.953 | 0.040 | 0.054 | M2 vs. M1 | 7.75 | 14 | .902 | 0.001 | -0.001 | 0.002 |
| M3: strong invariance | 648.52 | 361 | 1.10 | 0.924 | 0.050 | 0.060 | M3 vs. M2 | 145.54 | 14 | <.001 | -0.029 | 0.010 | 0.006 |
| M4: strict invariance | 690.75 | 381 | 1.11 | 0.918 | 0.050 | 0.073 | M4 vs. M3 | 41.08 | 20 | .004 | -0.006 | 0.000 | 0.013 |
| *Fall 2020:* | | | | | | | | | | | | | |
| M1: configural invariance | 634.18 | 333 | 1.09 | 0.951 | 0.047 | 0.050 |  |  |  |  |  |  |  |
| M2: weak invariance | 643.22 | 347 | 1.09 | 0.951 | 0.045 | 0.051 | M2 vs. M1 | 10.19 | 14 | .748 | 0.000 | -0.002 | 0.001 |
| M3: strong invariance | 749.22 | 361 | 1.09 | 0.936 | 0.051 | 0.053 | M3 vs. M2 | 120.43 | 14 | <.001 | -0.015 | 0.006 | 0.002 |
| M4: strict invariance | 773.12 | 381 | 1.12 | 0.936 | 0.050 | 0.059 | M4 vs. M3 | 29.43 | 20 | .080 | 0.000 | -0.001 | 0.006 |

*Note*. Residual correlations were allowed for items indicating the same value component (i.e., intrinsic value, attainment value, and utility value) and for the same items across time.

**Table C11**

*Tests of Measurement Invariance for SEVT Scales in the Most Important Course Across Groups for FG and CG Students*

| Model | χ² | *df* | SCF | CFI | RMSEA | SRMR | Model  comparison | Δχ² | Δdf | *p* | ΔCFI | ΔRMSEA | ΔSRMR |
| --- | --- | --- | --- | --- | --- | --- | --- | --- | --- | --- | --- | --- | --- |
| *Fall 2019* | | | | | | | | | | | | | |
| T1 |  |  |  |  |  |  |  |  |  |  |  |  |  |
| M1: configural invariance | 127.15 | 58 | 1.08 | 0.926 | 0.088 | 0.065 |  |  |  |  |  |  |  |
| M2: weak invariance | 134.29 | 65 | 1.09 | 0.926 | 0.084 | 0.069 | M2 vs. M1 | 7.57 | 7 | .372 | 0.000 | -0.004 | 0.004 |
| M3: strong invariance | 153.38 | 72 | 1.08 | 0.913 | 0.086 | 0.075 | M3 vs. M2 | 19.63 | 7 | .006 | -0.013 | 0.002 | 0.006 |
| M4: strict invariance | 176.85 | 82 | 1.10 | 0.899 | 0.087 | 0.107 | M4 vs. M3 | 23.25 | 10 | .010 | -0.014 | 0.001 | 0.032 |
| T2 |  |  |  |  |  |  |  |  |  |  |  |  |  |
| M1: configural invariance | 75.53 | 58 | 1.13 | 0.980 | 0.048 | 0.046 |  |  |  |  |  |  |  |
| M2: weak invariance | 88.88 | 65 | 1.14 | 0.973 | 0.053 | 0.069 | M2 vs. M1 | 13.17 | 7 | .068 | -0.007 | 0.005 | 0.023 |
| M3: strong invariance | 105.07 | 72 | 1.12 | 0.962 | 0.060 | 0.077 | M3 vs. M2 | 17.27 | 7 | .016 | -0.011 | 0.007 | 0.008 |
| M4: strict invariance | 110.84 | 82 | 1.16 | 0.967 | 0.052 | 0.086 | M4 vs. M3 | 7.71 | 10 | .657 | 0.005 | -0.008 | 0.009 |
| T3 |  |  |  |  |  |  |  |  |  |  |  |  |  |
| M1: configural invariance | 109.67 | 58 | 1.12 | 0.947 | 0.082 | 0.069 |  |  |  |  |  |  |  |
| M2: weak invariance | 121.06 | 65 | 1.10 | 0.942 | 0.080 | 0.077 | M2 vs. M1 | 11.19 | 7 | .131 | -0.005 | -0.002 | 0.008 |
| M3: strong invariance | 135.89 | 72 | 1.09 | 0.934 | 0.082 | 0.078 | M3 vs. M2 | 15.14 | 7 | .034 | -0.008 | 0.002 | 0.001 |
| M4: strict invariance | 137.59 | 82 | 1.14 | 0.942 | 0.071 | 0.082 | M4 vs. M3 | 5.73 | 10 | .837 | 0.008 | -0.011 | 0.004 |
| *Fall 2020* | | | | | | | | | | | | | |
| T1 |  |  |  |  |  |  |  |  |  |  |  |  |  |
| M1: configural invariance | 121.73 | 58 | 1.26 | 0.945 | 0.077 | 0.054 |  |  |  |  |  |  |  |
| M2: weak invariance | 127.05 | 65 | 1.25 | 0.947 | 0.072 | 0.061 | M2 vs. M1 | 4.88 | 7 | .674 | 0.002 | -0.005 | 0.007 |
| M3: strong invariance | 133.51 | 72 | 1.23 | 0.947 | 0.068 | 0.063 | M3 vs. M2 | 4.84 | 7 | .680 | 0.000 | -0.004 | 0.002 |
| M4: strict invariance | 135.56 | 82 | 1.27 | 0.954 | 0.059 | 0.079 | M4 vs. M3 | 5.47 | 10 | .857 | 0.007 | -0.009 | 0.016 |
| T2 |  |  |  |  |  |  |  |  |  |  |  |  |  |
| M1: configural invariance | 180.32 | 58 | 1.13 | 0.910 | 0.110 | 0.069 |  |  |  |  |  |  |  |
| M2: weak invariance | 186.70 | 65 | 1.15 | 0.910 | 0.104 | 0.074 | M2 vs. M1 | 8.25 | 7 | .311 | 0.000 | -0.006 | 0.005 |
| M3: strong invariance | 194.32 | 72 | 1.13 | 0.910 | 0.099 | 0.076 | M3 vs. M2 | 5.74 | 7 | .570 | 0.000 | -0.005 | 0.002 |
| M4: strict invariance | 200.00 | 82 | 1.14 | 0.913 | 0.091 | 0.079 | M4 vs. M3 | 7.17 | 10 | .709 | 0.003 | -0.008 | 0.003 |
| T3 |  |  |  |  |  |  |  |  |  |  |  |  |  |
| M1: configural invariance | 142.10 | 58 | 1.16 | 0.934 | 0.099 | 0.053 |  |  |  |  |  |  |  |
| M2: weak invariance | 156.79 | 65 | 1.14 | 0.928 | 0.098 | 0.066 | M2 vs. M1 | 14.44 | 7 | .044 | -0.006 | -0.001 | 0.013 |
| M3: strong invariance | 167.40 | 72 | 1.13 | 0.925 | 0.095 | 0.071 | M3 vs. M2 | 9.63 | 7 | .210 | -0.003 | -0.003 | 0.005 |
| M4: strict invariance | 168.85 | 82 | 1.22 | 0.932 | 0.085 | 0.077 | M4 vs. M3 | 9.40 | 10 | .494 | 0.007 | -0.010 | 0.006 |

**Table C12**

*Tests of Measurement Invariance for SEVT Scales in the Most Important Course Across Groups for Transfer and Non-transfer Students*

| Model | χ² | *df* | SCF | CFI | RMSEA | SRMR | Model  comparison | Δχ² | Δdf | *p* | ΔCFI | ΔRMSEA | ΔSRMR |
| --- | --- | --- | --- | --- | --- | --- | --- | --- | --- | --- | --- | --- | --- |
| *Fall 2019* | | | | | | | | | | | | | |
| T1 |  |  |  |  |  |  |  |  |  |  |  |  |  |
| M1: configural invariance^a^ | 136.13 | 58 | 1.08 | 0.920 | 0.094 | 0.064 |  |  |  |  |  |  |  |
| M2: weak invariance | 146.87 | 65 | 1.05 | 0.916 | 0.090 | 0.075 | M2 vs. M1 | 9.18 | 7 | .240 | -0.004 | -0.004 | 0.011 |
| M3: strong invariance | 156.78 | 72 | 1.05 | 0.913 | 0.087 | 0.079 | M3 vs. M2 | 9.59 | 7 | .213 | -0.003 | -0.003 | 0.004 |
| M4: strict invariance | 178.24 | 82 | 1.09 | 0.902 | 0.087 | 0.119 | M4 vs. M3 | 21.52 | 10 | .018 | -0.011 | 0.000 | 0.040 |
| T2 |  |  |  |  |  |  |  |  |  |  |  |  |  |
| M1: configural invariance | 58.42 | 58 | 1.09 | 1.000 | 0.007 | 0.042 |  |  |  |  |  |  |  |
| M2: weak invariance | 71.60 | 65 | 1.08 | 0.993 | 0.028 | 0.067 | M2 vs. M1 | 13.67 | 7 | .057 | -0.007 | 0.021 | 0.025 |
| M3: strong invariance | 80.96 | 72 | 1.08 | 0.990 | 0.031 | 0.073 | M3 vs. M2 | 9.40 | 7 | .225 | -0.003 | 0.003 | 0.006 |
| M4: strict invariance | 108.38 | 82 | 1.13 | 0.971 | 0.050 | 0.093 | M4 vs. M3 | 23.27 | 10 | .010 | -0.019 | 0.019 | 0.020 |
| T3 |  |  |  |  |  |  |  |  |  |  |  |  |  |
| M1: configural invariance | 109.55 | 58 | 0.97 | 0.950 | 0.081 | 0.071 |  |  |  |  |  |  |  |
| M2: weak invariance | 110.35 | 65 | 1.02 | 0.956 | 0.072 | 0.069 | M2 vs. M1 | 4.36 | 7 | .738 | 0.006 | -0.009 | -0.002 |
| M3: strong invariance | 115.70 | 72 | 1.01 | 0.958 | 0.067 | 0.072 | M3 vs. M2 | 4.77 | 7 | .687 | 0.002 | -0.005 | 0.003 |
| M4: strict invariance | 155.32 | 82 | 1.07 | 0.929 | 0.081 | 0.093 | M4 vs. M3 | 33.10 | 10 | .000 | -0.029 | 0.014 | 0.021 |
| *Fall 2020* | | | | | | | | | | | | | |
| T1 |  |  |  |  |  |  |  |  |  |  |  |  |  |
| M1: configural invariance | 129.80 | 58 | 1.17 | 0.945 | 0.080 | 0.054 |  |  |  |  |  |  |  |
| M2: weak invariance | 130.08 | 65 | 1.19 | 0.950 | 0.072 | 0.056 | M2 vs. M1 | 1.62 | 7 | .978 | 0.005 | -0.008 | 0.002 |
| M3: strong invariance | 136.42 | 72 | 1.17 | 0.950 | 0.068 | 0.058 | M3 vs. M2 | 5.29 | 7 | .625 | 0.000 | -0.004 | 0.002 |
| M4: strict invariance | 139.09 | 82 | 1.23 | 0.956 | 0.060 | 0.070 | M4 vs. M3 | 7.01 | 10 | .724 | 0.006 | -0.008 | 0.012 |
| T2 |  |  |  |  |  |  |  |  |  |  |  |  |  |
| M1: configural invariance | 162.95 | 58 | 1.13 | 0.927 | 0.100 | 0.064 |  |  |  |  |  |  |  |
| M2: weak invariance | 177.86 | 65 | 1.12 | 0.922 | 0.098 | 0.074 | M2 vs. M1 | 14.82 | 7 | .038 | -0.005 | -0.002 | 0.010 |
| M3: strong invariance | 189.11 | 72 | 1.12 | 0.919 | 0.094 | 0.077 | M3 vs. M2 | 10.79 | 7 | .148 | -0.003 | -0.004 | 0.003 |
| M4: strict invariance | 202.22 | 82 | 1.13 | 0.917 | 0.090 | 0.073 | M4 vs. M3 | 13.85 | 10 | .180 | -0.002 | -0.004 | -0.004 |
| T3 |  |  |  |  |  |  |  |  |  |  |  |  |  |
| M1: configural invariance | 171.16 | 58 | 1.06 | 0.917 | 0.112 | 0.056 |  |  |  |  |  |  |  |
| M2: weak invariance | 178.39 | 65 | 1.06 | 0.917 | 0.106 | 0.061 | M2 vs. M1 | 7.13 | 7 | .415 | 0.000 | -0.006 | 0.005 |
| M3: strong invariance | 183.23 | 72 | 1.06 | 0.919 | 0.100 | 0.064 | M3 vs. M2 | 4.98 | 7 | .662 | 0.002 | -0.006 | 0.003 |
| M4: strict invariance | 167.74 | 82 | 1.20 | 0.937 | 0.082 | 0.064 | M4 vs. M3 | 2.79 | 10 | .986 | 0.018 | -0.018 | 0.000 |

*Note.* ^a^This model only converged if we fixed one residual variance for transfer students to a positive value. This issue of non-convergence was most likely due to the small number of transfer students in the sample in comparison to the number of parameters in this model. The more parsimonious models did not lead to convergence problems.

**Appendix D**

**Descriptive Statistics**

**Table D1**

*Descriptive Statistics for the Whole Sample in Fall 2019 and 2020*

|  | Fall 2019  (*n* = 320) | | | Fall 2020  *(n =* 417*)* | | |
| --- | --- | --- | --- | --- | --- | --- |
| Variable | *n* | *M* | *SD* | *n* | *M* | *SD* |
| High School GPA | 319 | 3.96 | 0.28 | 417 | 4.02 | 0.20 |
| Social support |  |  |  |  |  |  |
| Peer support T1 | 305 | 61.35 | 28.48 | 367 | 43.56 | 28.29 |
| Faculty support T1 | 304 | 57.49 | 21.46 | 371 | 52.11 | 23.65 |
| Peer support T2 | 293 | 64.34 | 28.54 | 355 | 40.82 | 30.22 |
| Faculty support T2 | 292 | 54.87 | 23.41 | 358 | 48.00 | 25.14 |
| Motivation most difficult course |  |  |  |  |  |  |
| Expectancy T1 | 308 | 4.33 | 1.23 | 391 | 4.42 | 1.11 |
| Positive STV T1 | 308 | 4.44 | 1.36 | 391 | 4.76 | 1.24 |
| Cost T1 | 309 | 5.08 | 1.36 | 391 | 5.21 | 1.20 |
| Expectancy T2 | 264 | 4.52 | 1.41 | 365 | 4.28 | 1.49 |
| Positive STV T2 | 260 | 4.44 | 1.54 | 363 | 4.08 | 1.44 |
| Cost T2 | 261 | 5.01 | 1.44 | 363 | 4.69 | 1.40 |
| Expectancy T3 | 274 | 4.22 | 1.30 | 313 | 4.44 | 1.40 |
| Positive STV T3 | 276 | 4.22 | 1.51 | 313 | 4.40 | 1.48 |
| Cost T3 | 276 | 4.81 | 1.44 | 311 | 4.86 | 1.40 |
| Motivation most important course |  |  |  |  |  |  |
| Expectancy T1 | 308 | 5.19 | 1.12 | 391 | 5.66 | 0.95 |
| Positive STV T1 | 308 | 5.15 | 1.22 | 391 | 5.45 | 1.14 |
| Cost T1 | 306 | 4.47 | 1.55 | 390 | 4.38 | 1.46 |
| Expectancy T2 | 259 | 4.98 | 1.38 | 364 | 4.89 | 1.42 |
| Positive STV T2 | 258 | 4.93 | 1.41 | 363 | 5.05 | 1.41 |
| Cost T2 | 260 | 4.43 | 1.71 | 362 | 4.00 | 1.61 |
| Expectancy T3 | 269 | 5.20 | 1.29 | 312 | 5.27 | 1.34 |
| Positive STV T3 | 270 | 5.27 | 1.31 | 312 | 5.12 | 1.42 |
| Cost T3 | 266 | 4.13 | 1.68 | 311 | 4.10 | 1.64 |

*Note.* GPA = grade point average; STV = subjective task value.

**Table D2**

Correlations Among All Study Variables (Below the Diagonal for Fall 2019 Data/Above the Diagonal for Fall 2020 Data)

| Variable | | 1 | 2 | 3 | 4 | 5 | 6 | 7 | 8 | 9 | 10 | 11 | 12 | 13 | 14 | 15 | 16 | 17 | 18 | 19 | 20 | 21 | 22 | 23 | 24 | 25 | 26 | 27 | 28 | 29 |
| --- | --- | --- | --- | --- | --- | --- | --- | --- | --- | --- | --- | --- | --- | --- | --- | --- | --- | --- | --- | --- | --- | --- | --- | --- | --- | --- | --- | --- | --- | --- |
| 1 | High School GPA |  | -.08 | -.02 | .00 | -.05 | -.02 | -.12 | -.08 | .00 | -.15 | -.05 | .01 | -.04 | .16 | .07 | -.04 | .05 | -.02 | -.05 | .01 | -.01 | .00 | .09 | -.08 | -.01 | .06 | -.10 | -.01 | .10 |
| 2 | Gender (1 = male) | .05 |  | -.06 | -.06 | .05 | -.02 | .00 | .05 | .11 | .04 | .12 | .00 | -.02 | -.19 | .00 | .05 | -.17 | .00 | .00 | -.07 | .04 | -.07 | -.18 | .08 | .00 | -.01 | -.01 | -.05 | -.09 |
| 3 | Latinx American | -.13 | -.11 |  | -.15 | -.28 | .30 | .04 | -.13 | -.06 | -.02 | -.07 | .00 | .04 | .07 | -.07 | .01 | .06 | -.03 | .02 | .02 | .05 | .17 | .07 | .04 | .10 | -.01 | .02 | .03 | -.07 |
| 4 | European American | -.14 | -.04 | -.13 |  | -.09 | -.10 | -.01 | .03 | -.05 | .06 | -.06 | .01 | -.01 | .06 | .05 | -.07 | -.04 | .06 | -.07 | -.04 | .05 | .00 | .01 | -.06 | -.07 | .01 | -.14 | -.12 | .05 |
| 5 | Other race/ethnicity | -.07 | -.07 | -.29 | -.07 |  | -.13 | .11 | .02 | .12 | .04 | .13 | .04 | -.07 | -.02 | .03 | -.06 | .01 | .00 | -.02 | .05 | .11 | .07 | .01 | .11 | .00 | -.04 | .06 | .06 | -.01 |
| 6 | FG student | -.03 | -.07 | .33 | .05 | -.24 |  | -.11 | .00 | -.03 | -.01 | -.08 | .02 | .03 | .02 | -.02 | .00 | .03 | .02 | .01 | .03 | -.07 | -.01 | .04 | -.07 | -.06 | .00 | -.02 | -.03 | -.07 |
| 7 | Transfer student | -.64 | -.07 | .03 | .09 | .16 | -.05 |  | -.07 | -.04 | -.01 | -.01 | .04 | .03 | -.01 | -.01 | .04 | -.02 | .10 | .09 | .07 | .03 | .05 | .05 | -.02 | .04 | -.02 | .00 | -.07 | .01 |
| 8 | Peer support T1 | .02 | .02 | .05 | -.02 | -.02 | .04 | .00 |  | .38 | .68 | .34 | .19 | .10 | -.06 | .09 | .03 | -.01 | .11 | .07 | -.09 | .10 | -.02 | .03 | .05 | .03 | .00 | .06 | .09 | .08 |
| 9 | Faculty support T1 | .01 | .11 | .04 | -.05 | .03 | .00 | -.02 | .43 |  | .33 | .66 | .32 | .18 | -.16 | .24 | .15 | -.07 | .27 | .19 | -.09 | .20 | .22 | -.04 | .23 | .19 | -.05 | .12 | .17 | -.01 |
| 10 | Peer support T2 | -.01 | .02 | .03 | -.09 | .05 | .08 | .03 | .65 | .37 |  | .38 | .14 | .00 | -.07 | .08 | -.01 | -.03 | .07 | -.03 | -.02 | .04 | .01 | .04 | .08 | .06 | .05 | .08 | .07 | .07 |
| 11 | Faculty support T2 | -.02 | .04 | -.03 | -.03 | .09 | -.04 | .04 | .38 | .65 | .42 |  | .25 | .16 | -.13 | .17 | .14 | -.10 | .23 | .15 | -.12 | .17 | .14 | -.04 | .27 | .16 | -.09 | .12 | .11 | -.08 |
| 12 | Expectancy DC T1 | -.16 | .05 | .10 | -.10 | .08 | -.07 | .20 | .10 | .22 | .09 | .24 |  | .40 | -.18 | .29 | .21 | -.15 | .47 | .32 | -.23 | .27 | .13 | .06 | .14 | .06 | -.09 | .19 | .11 | -.01 |
| 13 | Positive STV DC T1 | -.10 | .00 | .08 | .00 | -.09 | .07 | .16 | .11 | .23 | .15 | .17 | .43 |  | .02 | .22 | .62 | .07 | .32 | .64 | -.03 | .19 | .37 | .16 | .05 | .21 | .10 | .06 | .17 | .16 |
| 14 | Cost DC T1 | .14 | -.04 | .00 | .01 | -.07 | .07 | -.10 | -.02 | -.01 | .10 | -.05 | -.21 | .18 |  | -.13 | -.01 | .38 | -.14 | -.09 | .45 | .03 | .12 | .49 | -.05 | .05 | .14 | -.02 | .08 | .27 |
| 15 | Expectancy DC T2 | .05 | .13 | -.07 | .11 | .11 | -.05 | .06 | .05 | .18 | .04 | .20 | .28 | .20 | -.11 |  | .34 | -.25 | .53 | .37 | -.29 | .13 | .05 | -.03 | .14 | .12 | .07 | .11 | .14 | .08 |
| 16 | Positive STV DC T2 | -.03 | .03 | .06 | -.03 | .02 | .11 | .13 | .10 | .21 | .19 | .15 | .28 | .58 | .09 | .26 |  | .11 | .33 | .67 | -.05 | .10 | .26 | .10 | .12 | .38 | .17 | .04 | .22 | .17 |
| 17 | Cost DC T2 | .01 | -.06 | -.12 | -.02 | .07 | .04 | -.01 | -.07 | -.06 | .18 | .03 | -.16 | .08 | .42 | -.28 | .12 |  | -.19 | -.01 | .52 | .02 | .13 | .23 | .01 | .16 | .29 | .03 | .12 | .23 |
| 18 | Expectancy DC T3 | -.04 | .07 | .05 | -.04 | .07 | -.10 | .12 | .07 | .14 | .06 | .16 | .39 | .19 | -.12 | .34 | .22 | -.15 |  | .54 | -.33 | .15 | .11 | .01 | .11 | .07 | -.05 | .16 | .11 | .01 |
| 19 | Positive STV DC T3 | .01 | -.05 | .05 | .00 | .02 | .06 | .07 | .11 | .22 | .17 | .17 | .30 | .49 | .12 | .18 | .55 | .06 | .45 |  | -.10 | .06 | .27 | .18 | .03 | .24 | .14 | .07 | .25 | .20 |
| 20 | Cost DC T3 | -.08 | -.01 | -.09 | .02 | .05 | .06 | .04 | .10 | .08 | .22 | .09 | -.09 | .14 | .43 | -.19 | .08 | .54 | -.19 | .08 |  | -.04 | .05 | .26 | .00 | .11 | .09 | .02 | .09 | .30 |
| 21 | Expectancy IC T1 | -.05 | .06 | .04 | -.02 | .11 | -.10 | .11 | .14 | .17 | .11 | .20 | .40 | .10 | -.10 | .25 | .04 | -.08 | .29 | .01 | -.01 |  | .43 | -.26 | .42 | .28 | -.13 | .37 | .26 | -.06 |
| 22 | Positive STV IC T1 | -.09 | -.01 | .16 | -.04 | -.06 | .02 | .11 | .15 | .19 | .24 | .16 | .18 | .55 | .24 | .04 | .39 | .21 | .14 | .31 | .21 | .37 |  | .15 | .19 | .60 | .05 | .26 | .59 | .04 |
| 23 | Cost IC T1 | .01 | -.06 | .06 | -.07 | -.06 | .12 | -.02 | .01 | .00 | .12 | -.07 | -.01 | .31 | .58 | -.02 | .25 | .36 | -.06 | .27 | .36 | -.29 | .22 |  | -.14 | .02 | .31 | -.11 | .08 | .40 |
| 24 | Expectancy IC T2 | .00 | .03 | .09 | .01 | .05 | -.04 | .12 | .05 | .18 | .08 | .23 | .18 | .02 | -.07 | .49 | .04 | -.20 | .31 | .10 | -.06 | .30 | .09 | -.15 |  | .33 | -.21 | .61 | .30 | -.23 |
| 25 | Positive STV IC T2 | -.02 | -.05 | .11 | .03 | -.04 | .04 | .05 | .17 | .21 | .19 | .19 | .05 | .34 | .16 | .10 | .52 | .10 | .19 | .37 | .19 | .21 | .54 | .06 | .31 |  | .15 | .39 | .74 | .01 |
| 26 | Cost IC T2 | .01 | .04 | -.08 | -.03 | .04 | .10 | -.01 | -.02 | .01 | .15 | .00 | -.16 | .21 | .47 | -.12 | .33 | .57 | -.12 | .21 | .45 | -.20 | .14 | .52 | -.26 | .21 |  | -.18 | .06 | .62 |
| 27 | Expectancy IC T3 | -.04 | .12 | .10 | .00 | .08 | .04 | .09 | .11 | .23 | .11 | .13 | .18 | .11 | .02 | .22 | .06 | -.09 | .37 | .16 | .05 | .38 | .22 | -.04 | .34 | .17 | -.20 |  | .52 | -.30 |
| 28 | Positive STV IC T3 | -.06 | .04 | .13 | -.03 | .06 | -.04 | .12 | .11 | .19 | .23 | .15 | .19 | .46 | .22 | .01 | .46 | .13 | .17 | .40 | .21 | .25 | .64 | .16 | .14 | .60 | .14 | .42 |  | .03 |
| 29 | Cost IC T3 | .04 | .00 | -.01 | -.01 | -.03 | .06 | .00 | .04 | .05 | .16 | .06 | .02 | .28 | .43 | -.01 | .28 | .39 | .02 | .27 | .49 | -.06 | .19 | .49 | -.13 | .23 | .51 | -.16 | .15 |  |

*Note.* GPA = grade point average; FG = first-generation; DC = most difficult course; STV = subjective task value; IC = most important course.

**Table D3**

*Descriptive Statistics for Student Subgroups in the Fall 2019 Data*

|  | FG students  (*n* = 180) | | | CG students  (*n* = 136) | | | Transfer students  (*n* = 65) | | | Non-transfer students  (*n* = 255) | | |
| --- | --- | --- | --- | --- | --- | --- | --- | --- | --- | --- | --- | --- |
| Variable | *n* | *M* | *SD* | *n* | *M* | *SD* | *n* | *M* | *SD* | *n* | *M* | *SD* |
| High School GPA | 180 | 3.95 | 0.29 | 136 | 3.97 | 0.27 | 64 | 3.60 | 0.28 | 255 | 4.05 | 0.20 |
| Social support |  |  |  |  |  |  |  |  |  |  |  |  |
| Peer support T1 | 171 | 62.18 | 28.30 | 130 | 59.61 | 28.89 | 62 | 60.97 | 29.77 | 243 | 61.45 | 28.20 |
| Faculty support T1 | 170 | 57.44 | 22.64 | 130 | 56.94 | 19.78 | 62 | 56.76 | 23.14 | 242 | 57.68 | 21.06 |
| Peer support T2 | 164 | 66.39 | 28.11 | 125 | 61.04 | 29.17 | 60 | 64.40 | 27.25 | 233 | 64.32 | 28.92 |
| Faculty support T2 | 163 | 54.36 | 23.97 | 125 | 55.19 | 22.85 | 60 | 56.26 | 23.41 | 232 | 54.51 | 23.44 |
| Motivation most difficult course |  |  |  |  |  |  |  |  |  |  |  |  |
| Expectancy T1 | 177 | 4.27 | 1.17 | 128 | 4.41 | 1.31 | 64 | 4.81 | 1.22 | 244 | 4.21 | 1.20 |
| Positive STV T1 | 177 | 4.52 | 1.37 | 128 | 4.30 | 1.35 | 64 | 4.87 | 1.39 | 244 | 4.33 | 1.33 |
| Cost T1 | 177 | 5.15 | 1.38 | 129 | 4.97 | 1.33 | 64 | 4.84 | 1.32 | 245 | 5.15 | 1.37 |
| Expectancy T2 | 145 | 4.43 | 1.50 | 115 | 4.62 | 1.30 | 57 | 4.73 | 1.49 | 207 | 4.46 | 1.39 |
| Positive STV T2 | 144 | 4.55 | 1.51 | 112 | 4.25 | 1.55 | 57 | 4.80 | 1.63 | 203 | 4.34 | 1.50 |
| Cost T2 | 143 | 5.03 | 1.42 | 114 | 4.95 | 1.49 | 56 | 4.91 | 1.43 | 205 | 5.04 | 1.45 |
| Expectancy T3 | 154 | 4.10 | 1.20 | 117 | 4.33 | 1.40 | 55 | 4.57 | 1.48 | 219 | 4.13 | 1.23 |
| Positive STV T3 | 156 | 4.28 | 1.52 | 117 | 4.11 | 1.51 | 55 | 4.49 | 1.72 | 221 | 4.16 | 1.45 |
| Cost T3 | 156 | 4.91 | 1.48 | 117 | 4.65 | 1.38 | 55 | 4.92 | 1.50 | 221 | 4.79 | 1.42 |
| Motivation most important course |  |  |  |  |  |  |  |  |  |  |  |  |
| Expectancy T1 | 176 | 5.09 | 1.09 | 129 | 5.32 | 1.16 | 63 | 5.44 | 1.09 | 245 | 5.12 | 1.12 |
| Positive STV T1 | 176 | 5.17 | 1.23 | 129 | 5.11 | 1.21 | 63 | 5.41 | 1.24 | 245 | 5.08 | 1.20 |
| Cost T1 | 175 | 4.63 | 1.54 | 128 | 4.26 | 1.55 | 62 | 4.43 | 1.47 | 244 | 4.48 | 1.57 |
| Expectancy T2 | 141 | 4.93 | 1.44 | 114 | 5.03 | 1.31 | 57 | 5.32 | 1.25 | 202 | 4.88 | 1.40 |
| Positive STV T2 | 141 | 4.98 | 1.35 | 113 | 4.81 | 1.48 | 57 | 4.99 | 1.45 | 201 | 4.91 | 1.40 |
| Cost T2 | 142 | 4.55 | 1.77 | 114 | 4.25 | 1.64 | 57 | 4.26 | 1.71 | 203 | 4.47 | 1.71 |
| Expectancy T3 | 151 | 5.26 | 1.18 | 115 | 5.11 | 1.42 | 55 | 5.44 | 1.23 | 214 | 5.14 | 1.30 |
| Positive STV T3 | 152 | 5.23 | 1.40 | 115 | 5.30 | 1.19 | 55 | 5.60 | 1.36 | 215 | 5.19 | 1.28 |
| Cost T3 | 150 | 4.22 | 1.82 | 113 | 4.00 | 1.49 | 55 | 4.17 | 1.63 | 211 | 4.11 | 1.70 |

*Note.* FG = first-generation; CG = continuing-generation; GPA = grade point average; STV = subjective task value.

**Table D4**

*Descriptive Statistics for Student Subgroups in the Fall 2020 Data*

|  | FG students  (*n* = 224) | | | CG students  (*n* = 172) | | | Transfer students  (*n* = 102) | | | Non-transfer students  (*n* = 314) | | |
| --- | --- | --- | --- | --- | --- | --- | --- | --- | --- | --- | --- | --- |
| Variable | *n* | *M* | *SD* | *n* | *M* | *SD* | *n* | *M* | *SD* | *n* | *M* | *SD* |
| High School GPA | 224 | 4.01 | 0.19 | 172 | 4.02 | 0.22 | 102 | 3.97 | 0.14 | 314 | 4.03 | 0.22 |
| Social support |  |  |  |  |  |  |  |  |  |  |  |  |
| Peer support T1 | 198 | 43.49 | 28.81 | 154 | 43.43 | 26.94 | 92 | 40.15 | 31.91 | 275 | 44.70 | 26.94 |
| Faculty support T1 | 201 | 51.62 | 23.99 | 155 | 53.26 | 23.27 | 93 | 50.72 | 26.63 | 278 | 52.58 | 22.59 |
| Peer support T2 | 189 | 40.37 | 30.54 | 149 | 41.47 | 30.09 | 87 | 41.20 | 31.49 | 268 | 40.70 | 29.85 |
| Faculty support T2 | 192 | 45.69 | 24.51 | 149 | 50.40 | 25.46 | 88 | 47.94 | 27.24 | 270 | 48.01 | 24.47 |
| Motivation most difficult course |  |  |  |  |  |  |  |  |  |  |  |  |
| Expectancy T1 | 212 | 4.42 | 1.12 | 161 | 4.37 | 1.11 | 97 | 4.48 | 1.22 | 293 | 4.40 | 1.08 |
| Positive STV T1 | 212 | 4.80 | 1.20 | 161 | 4.74 | 1.31 | 97 | 4.84 | 1.36 | 293 | 4.73 | 1.20 |
| Cost T1 | 212 | 5.23 | 1.22 | 161 | 5.19 | 1.20 | 97 | 5.21 | 1.20 | 293 | 5.21 | 1.21 |
| Expectancy T2 | 194 | 4.24 | 1.45 | 154 | 4.33 | 1.57 | 86 | 4.27 | 1.56 | 279 | 4.29 | 1.47 |
| Positive STV T2 | 194 | 4.07 | 1.41 | 152 | 4.08 | 1.52 | 85 | 4.25 | 1.50 | 278 | 4.03 | 1.42 |
| Cost T2 | 194 | 4.72 | 1.35 | 152 | 4.65 | 1.46 | 85 | 4.69 | 1.41 | 278 | 4.69 | 1.40 |
| Expectancy T3 | 173 | 4.49 | 1.37 | 124 | 4.32 | 1.45 | 77 | 4.71 | 1.29 | 236 | 4.35 | 1.43 |
| Positive STV T3 | 173 | 4.41 | 1.47 | 124 | 4.31 | 1.54 | 77 | 4.71 | 1.48 | 236 | 4.29 | 1.47 |
| Cost T3 | 173 | 4.90 | 1.26 | 122 | 4.78 | 1.59 | 76 | 5.07 | 1.44 | 235 | 4.80 | 1.39 |
| Motivation most important course |  |  |  |  |  |  |  |  |  |  |  |  |
| Expectancy T1 | 212 | 5.59 | 0.99 | 161 | 5.74 | 0.92 | 97 | 5.71 | 0.97 | 293 | 5.65 | 0.95 |
| Positive STV T1 | 212 | 5.44 | 1.13 | 161 | 5.48 | 1.16 | 97 | 5.55 | 1.14 | 293 | 5.42 | 1.14 |
| Cost T1 | 212 | 4.41 | 1.45 | 160 | 4.33 | 1.46 | 97 | 4.52 | 1.40 | 292 | 4.34 | 1.48 |
| Expectancy T2 | 194 | 4.79 | 1.51 | 153 | 5.00 | 1.25 | 86 | 4.87 | 1.52 | 278 | 4.90 | 1.39 |
| Positive STV T2 | 193 | 4.96 | 1.41 | 153 | 5.16 | 1.36 | 86 | 5.21 | 1.52 | 277 | 5.00 | 1.37 |
| Cost T2 | 193 | 4.00 | 1.50 | 152 | 4.06 | 1.70 | 86 | 3.94 | 1.74 | 276 | 4.02 | 1.57 |
| Expectancy T3 | 172 | 5.25 | 1.35 | 124 | 5.28 | 1.37 | 78 | 5.23 | 1.47 | 234 | 5.28 | 1.30 |
| Positive STV T3 | 172 | 5.08 | 1.46 | 124 | 5.19 | 1.35 | 78 | 5.02 | 1.65 | 234 | 5.16 | 1.33 |
| Cost T3 | 172 | 3.95 | 1.58 | 123 | 4.23 | 1.70 | 78 | 4.19 | 1.61 | 233 | 4.07 | 1.65 |

*Note.* FG = first-generation; CG = continuing-generation; GPA = grade point average; STV = subjective task value.

**Appendix E**

**Analyses For Those Students Whose Most Difficult/Most Important Course Remained the Same Across Time in the Fall 2019 Data**

|  | Most Difficult Course (*n* = 111) | | | | | | | | Most Important Course (*n* = 154) | | | | | | | |
| --- | --- | --- | --- | --- | --- | --- | --- | --- | --- | --- | --- | --- | --- | --- | --- | --- |
|  | Peer support | | | | Faculty support | | | | Peer support | | | | Faculty support | | | |
|  | β | 95% CI | | *p* | β | 95% CI | | *p* | β | 95% CI | | *p* | β | 95% CI | | *p* |
| Expectancy |  |  |  |  |  |  |  |  |  |  |  |  |  |  |  |  |
| Stability coefficients |  |  |  |  |  |  |  |  |  |  |  |  |  |  |  |  |
| Support T1 🡪 Support T2 (a_12_) | 0.63 | [0.47, | 0.79] | <.001 | 0.67 | [0.52, | 0.82] | <.001 | 0.58 | [0.45, | 0.72] | <.001 | 0.72 | [0.61, | 0.82] | <.001 |
| Expectancy T1 🡪 Expectancy T2 (b_12_) | 0.24 | [0.03, | 0.45] | .027 | 0.19 | [-0.04, | 0.42] | .113 | 0.29 | [0.14, | 0.45] | <.001 | 0.27 | [0.11, | 0.42] | .001 |
| Expectancy T1 🡪 Expectancy T3 (b_13_) | 0.28 | [0.02, | 0.53] | .034 | 0.31 | [0.04, | 0.58] | .025 | 0.44 | [0.31, | 0.58] | <.001 | 0.43 | [0.29, | 0.58] | <.001 |
| Expectancy T2 🡪 Expectancy T3 (b_23_) | 0.19 | [-0.07, | 0.44] | .151 | 0.17 | [-0.10, | 0.45] | .208 | 0.30 | [0.10, | 0.50] | .003 | 0.28 | [0.08, | 0.48] | .005 |
| Cross-lagged coefficients |  |  |  |  |  |  |  |  |  |  |  |  |  |  |  |  |
| Support T1 🡪 Expectancy T2 (c_12_) | -0.06 | [-0.25, | 0.13] | .513 | 0.11 | [-0.10, | 0.32] | .309 | -0.11 | [-0.26, | 0.04] | .164 | 0.15 | [-0.04, | 0.34] | .111 |
| Support T1 🡪 Expectancy T3 (c_13_) | 0.16 | [-0.10, | 0.42] | .220 | -0.09 | [-0.30, | 0.11] | .370 | -0.02 | [-0.19, | 0.15] | .832 | 0.07 | [-0.10, | 0.24] | .407 |
| Support T2 🡪 Expectancy T3 (c_23_) | -0.12 | [-0.38, | 0.13] | .341 | 0.03 | [-0.21, | 0.27] | .842 | 0.09 | [-0.10, | 0.28] | .359 | -0.02 | [-0.21, | 0.17] | .835 |
| Expectancy T1 🡪 Support T2 (d_12_) | 0.01 | [-0.17, | 0.18] | .955 | 0.19 | [0.06, | 0.33] | .006 | 0.02 | [-0.12, | 0.15] | .826 | 0.03 | [-0.10, | 0.17] | .652 |
| Positive STV |  |  |  |  |  |  |  |  |  |  |  |  |  |  |  |  |
| Stability coefficients |  |  |  |  |  |  |  |  |  |  |  |  |  |  |  |  |
| Support T1 🡪 Support T2 (a_12_) | 0.61 | [0.46, | 0.77] | <.001 | 0.70 | [0.56, | 0.83] | <.001 | 0.57 | [0.44, | 0.70] | <.001 | 0.71 | [0.61, | 0.82] | <.001 |
| Positive STV T1 🡪 Positive STV T2 (b_12_) | 0.57 | [0.40, | 0.73] | <.001 | 0.56 | [0.38, | 0.73] | .292 | 0.47 | [0.33, | 0.61] | <.001 | 0.45 | [0.31, | 0.59] | <.001 |
| Positive STV T1 🡪 Positive STV T3 (b_13_) | 0.31 | [0.08, | 0.54] | .008 | 0.30 | [0.05, | 0.54] | .019 | 0.48 | [0.35, | 0.60] | <.001 | 0.49 | [0.37, | 0.62] | <.001 |
| Positive STV T2 🡪 Positive STV T3 (b_23_) | 0.45 | [0.23, | 0.68] | <.001 | 0.44 | [0.21, | 0.66] | <.001 | 0.40 | [0.28, | 0.52] | <.001 | 0.38 | [0.25, | 0.51] | <.001 |
| Cross-lagged coefficients |  |  |  |  |  |  |  |  |  |  |  |  |  |  |  |  |
| Support T1 🡪 Positive STV T2 (c_12_) | 0.08 | [-0.10, | 0.25] | .375 | 0.10 | [-0.12, | 0.32] | <.001 | -0.02 | [-0.19, | 0.16] | .852 | 0.12 | [-0.05, | 0.29] | .153 |
| Support T1 🡪 Positive STV T3 (c_13_) | -0.02 | [-0.22, | 0.18] | .841 | -0.01 | [-0.22, | 0.21] | .949 | -0.10 | [-0.22, | 0.03] | .121 | -0.02 | [-0.17, | 0.13] | .772 |
| Support T2 🡪 Positive STV T3 (c_23_) | -0.01 | [-0.21, | 0.19] | .936 | 0.10 | [-0.08, | 0.28] | .265 | 0.10 | [-0.06, | 0.26] | .206 | 0.06 | [-0.11, | 0.23] | .471 |
| Positive STV T1 🡪 Support T2 (d_12_) | 0.10 | [-0.05, | 0.25] | .175 | 0.09 | [-0.08, | 0.26] | .292 | 0.17 | [0.04, | 0.30] | .009 | 0.02 | [-0.09, | 0.14] | .711 |
| Cost |  |  |  |  |  |  |  |  |  |  |  |  |  |  |  |  |
| Stability coefficients |  |  |  |  |  |  |  |  |  |  |  |  |  |  |  |  |
| Support T1 🡪 Support T2 (a_12_) | 0.64 | [0.52, | 0.77] | <.001 | 0.71 | [0.57, | 0.85] | <.001 | 0.58 | [0.45, | 0.72] | <.001 | 0.72 | [0.61, | 0.83] | <.001 |
| Cost T1 🡪 Cost T2(b_12_) | 0.45 | [0.27, | 0.63] | <.001 | 0.46 | [0.28, | 0.61] | <.001 | 0.57 | [0.43, | 0.71] | <.001 | 0.56 | [0.41, | 0.70] | <.001 |
| Cost T1 🡪 Cost T3 (b_13_) | 0.13 | [-0.07, | 0.33] | .192 | 0.12 | [-0.09, | 0.33] | .254 | 0.26 | [0.12, | 0.41] | .003 | 0.27 | [0.10, | 0.43] | .002 |
| Cost T2 🡪 Cost T3 (b_23_) | 0.58 | [0.40, | 0.77] | <.001 | 0.56 | [0.36, | 0.76] | <.001 | 0.43 | [0.28, | 0.57] | <.001 | 0.43 | [0.27, | 0.60] | <.001 |
| Cross-lagged coefficients |  |  |  |  |  |  |  |  |  |  |  |  |  |  |  |  |
| Support T1 🡪 Cost T2 (c_12_) | -0.10 | [-0.26, | 0.07] | .241 | 0.00 | [-0.18, | 0.18] | .996 | -0.10 | [-0.24, | 0.05] | .192 | -0.08 | [-0.21, | 0.06] | .264 |
| Support T1 🡪 Cost T3 (c_13_) | 0.17 | [-0.01, | 0.35] | .060 | 0.06 | [-0.15, | 0.28] | .566 | 0.01 | [-0.17, | 0.19] | .948 | -0.10 | [-0.29, | 0.09] | .300 |
| Support T2 🡪 Cost T3 (c_23_) | 0.00 | [-0.19, | 0.19] | .996 | 0.05 | [-0.15, | 0.25] | .619 | 0.03 | [-0.15, | 0.21] | .722 | 0.13 | [-0.07, | 0.33] | .202 |
| Cost T1 🡪 Support T2 (d_12_) | 0.14 | [0.01, | 0.27] | .034 | 0.03 | [-0.15, | 0.20] | .765 | 0.06 | [-0.06, | 0.19] | .301 | -0.01 | [-0.10, | 0.09] | .935 |

*Note.* STV = subjective task value

**Appendix F**

**Missing Data Analyses**

**Table F1**

*Comparison of Students with Missing Data at One or Two Time Points and Students with Data Available at Three Time Points: Descriptive Statistics*

|  | Fall 2019 | | Fall 2020 | |
| --- | --- | --- | --- | --- |
| Variable | Data at 1-2 time points | Data at 3 time points | Data at 1-2 time points | Data at 3 time points |
| Gender |  |  |  |  |
| Female | 37 | 182 | 89 | 210 |
| Male | 10 | 90 | 35 | 80 |
| Race/ethnicity |  |  |  |  |
| Asian/Asian American | 13 | 138 | 59 | 142 |
| Hispanic | 24 | 87 | 40 | 91 |
| White non-Hispanic | 7 | 33 | 17 | 42 |
| Other | 2 | 8 | 6 | 13 |
| College generation |  |  |  |  |
| CG student | 20 | 116 | 53 | 119 |
| FG student | 26 | 154 | 64 | 160 |
| Transfer student status |  |  |  |  |
| Non-transfer student | 37 | 218 | 95 | 219 |
| Transfer student | 10 | 55 | 28 | 74 |
| High school GPA^a^ | 3.87 | 3.97 | 4.00 | 4.03 |

*Note.*CG = continuing-generation, FG = first-generation, GPA = grade point average.

^a^ Mean value is reported.

**Table F2**

*Comparison of Students with Missing Data at One or Two Time Points and Students with Data Available at Three Time Points: Test Statistics*

|  | Fall 2019 | | | Fall 2020 | | |
| --- | --- | --- | --- | --- | --- | --- |
| Variable | Statistic | *df* | *p* | Statistic | *df* | *p* |
| Gender | 2.60 | 1 | .107 | 0.02 | 1 | .894 |
| Race/ethnicity | 9.16 | 3 | .027 | 0.11 | 3 | .991 |
| College generation | 0.00 | 1 | .948 | 0.24 | 1 | .628 |
| Transfer student status | 0.03 | 1 | .859 | 0.29 | 1 | .590 |
| High school GPA | 2.44 | 317 | .015 | 1.22 | 415 | .222 |

*Note.* GPA = grade point average. Chi-square statistics are reported for categorical variables, t statistics are reported for continuous variables.

**Table F3**

*Analyses With the Subsample of Students With Data Available for at Least Two Time Points: Fall 2019 Data (n = 307)*

|  | Most Difficult Course | | | | | | | | Most Important Course | | | | | | | |
| --- | --- | --- | --- | --- | --- | --- | --- | --- | --- | --- | --- | --- | --- | --- | --- | --- |
|  | Peer support | | | | Faculty support | | | | Peer support | | | | Faculty support | | | |
|  | β | 95% CI | | *p* | β | 95% CI | | *p* | β | 95% CI | | *p* | β | 95% CI | | *p* |
| Expectancy |  |  |  |  |  |  |  |  |  |  |  |  |  |  |  |  |
| Stability coefficients |  |  |  |  |  |  |  |  |  |  |  |  |  |  |  |  |
| Support T1 🡪 Support T2 (a_12_) | 0.71 | [0.62, | 0.79] | <.001 | 0.62 | [0.53, | 0.72] | <.001 | 0.70 | [0.62, | 0.78] | <.001 | 0.64 | [0.55, | 0.73] | <.001 |
| Expectancy T1 🡪 Expectancy T2 (b_12_) | 0.28 | [0.15, | 0.42] | <.001 | 0.26 | [0.11, | 0.40] | <.001 | 0.28 | [0.17, | 0.39] | <.001 | 0.26 | [0.15, | 0.38] | <.001 |
| Expectancy T1 🡪 Expectancy T3 (b_13_) | 0.25 | [0.11, | 0.39] | <.001 | 0.25 | [0.11, | 0.39] | .001 | 0.27 | [0.11, | 0.43] | .001 | 0.26 | [0.11, | 0.41] | .001 |
| Expectancy T2 🡪 Expectancy T3 (b_23_) | 0.29 | [0.16, | 0.43] | <.001 | 0.28 | [0.14, | 0.43] | <.001 | 0.27 | [0.16, | 0.38] | <.001 | 0.27 | [0.16, | 0.38] | <.001 |
| Cross-lagged coefficients |  |  |  |  |  |  |  |  |  |  |  |  |  |  |  |  |
| Support T1 🡪 Expectancy T2 (c_12_) | 0.01 | [-0.12, | 0.13] | .922 | 0.10 | [-0.03, | 0.23] | .122 | -0.01 | [-0.13, | 0.10] | .853 | 0.12 | [0.00, | 0.25] | .053 |
| Support T1 🡪 Expectancy T3 (c_13_) | 0.10 | [-0.07, | 0.27] | .243 | 0.03 | [-0.13, | 0.19] | .703 | 0.00 | [-0.16, | 0.17] | .982 | -0.13 | [-0.27, | 0.02] | .098 |
| Support T2 🡪 Expectancy T3 (c_23_) | -0.11 | [-0.27, | 0.05] | .188 | 0.01 | [-0.13, | 0.14] | .939 | 0.06 | [-0.10, | 0.22] | .469 | 0.20 | [0.06, | 0.34] | .005 |
| Expectancy T1 🡪 Support T2 (d_12_) | 0.02 | [-0.07, | 0.12] | .654 | 0.12 | [0.02, | 0.22] | .024 | 0.02 | [-0.06, | 0.11] | .587 | 0.09 | [-0.01, | 0.18] | .083 |
| Positive STV |  |  |  |  |  |  |  |  |  |  |  |  |  |  |  |  |
| Stability coefficients |  |  |  |  |  |  |  |  |  |  |  |  |  |  |  |  |
| Support T1 🡪 Support T2 (a_12_) | 0.70 | [0.62, | 0.79] | <.001 | 0.64 | [0.54, | 0.73] | <.001 | 0.69 | [0.60, | 0.77] | <.001 | 0.64 | [0.55, | 0.74] | <.001 |
| Positive STV T1 🡪 Positive STV T2 (b_12_) | 0.56 | [0.45, | 0.67] | <.001 | 0.55 | [0.43, | 0.66] | <.001 | 0.52 | [0.42, | 0.62] | <.001 | 0.51 | [0.41, | 0.61] | <.001 |
| Positive STV T1 🡪 Positive STV T3 (b_13_) | 0.37 | [0.22, | 0.53] | <.001 | 0.38 | [0.22, | 0.54] | <.001 | 0.39 | [0.27, | 0.50] | <.001 | 0.38 | [0.27, | 0.50] | <.001 |
| Positive STV T2 🡪 Positive STV T3 (b_23_) | 0.27 | [0.13, | 0.41] | <.001 | 0.24 | [0.09, | 0.40] | .002 | 0.41 | [0.30, | 0.52] | <.001 | 0.41 | [0.30, | 0.53] | <.001 |
| Cross-lagged coefficients |  |  |  |  |  |  |  |  |  |  |  |  |  |  |  |  |
| Support T1 🡪 Positive STV T2 (c_12_) | 0.04 | [-0.07, | 0.16] | .442 | 0.07 | [-0.05, | 0.20] | .251 | 0.10 | [-0.02, | 0.21] | .106 | 0.11 | [0.00, | 0.22] | .058 |
| Support T1 🡪 Positive STV T3 (c_13_) | 0.10 | [-0.05, | 0.25] | .198 | 0.02 | [-0.12, | 0.15] | .812 | 0.10 | [-0.04, | 0.23] | .155 | -0.03 | [-0.14, | 0.08] | .585 |
| Support T2 🡪 Positive STV T3 (c_23_) | -0.07 | [-0.23, | 0.09] | .372 | 0.08 | [-0.05, | 0.22] | .223 | -0.06 | [-0.18, | 0.06] | .323 | 0.05 | [-0.06, | 0.16] | .355 |
| Positive STV T1 🡪 Support T2 (d_12_) | 0.06 | [-0.03, | 0.14] | .184 | 0.04 | [-0.06, | 0.13] | .459 | 0.11 | [0.03, | 0.20] | .011 | 0.04 | [-0.06, | 0.14] | .449 |
| Cost |  |  |  |  |  |  |  |  |  |  |  |  |  |  |  |  |
| Stability coefficients |  |  |  |  |  |  |  |  |  |  |  |  |  |  |  |  |
| Support T1 🡪 Support T2 (a_12_) | 0.72 | [0.63, | 0.80] | <.001 | 0.64 | [0.55, | 0.74] | <.001 | 0.71 | [0.63, | 0.79] | <.001 | 0.65 | [0.56, | 0.74] | <.001 |
| Cost T1 🡪 Cost T2(b_12_) | 0.42 | [0.29, | 0.54] | <.001 | 0.41 | [0.29, | 0.54] | <.001 | 0.53 | [0.41, | 0.65] | <.001 | 0.53 | [0.41, | 0.65] | <.001 |
| Cost T1 🡪 Cost T3 (b_13_) | 0.42 | [0.29, | 0.56] | <.001 | 0.41 | [0.27, | 0.55] | <.001 | 0.37 | [0.24, | 0.50] | <.001 | 0.39 | [0.26, | 0.51] | <.001 |
| Cost T2 🡪 Cost T3 (b_23_) | 0.26 | [0.13, | 0.39] | <.001 | 0.26 | [0.13, | 0.39] | <.001 | 0.31 | [0.19, | 0.42] | <.001 | 0.31 | [0.19, | 0.42] | <.001 |
| Cross-lagged coefficients |  |  |  |  |  |  |  |  |  |  |  |  |  |  |  |  |
| Support T1 🡪 Cost T2 (c_12_) | -0.02 | [-0.13, | 0.09] | .717 | -0.04 | [-0.15, | 0.08] | .512 | 0.01 | [-0.09, | 0.11] | .834 | 0.01 | [-0.09, | 0.11] | .831 |
| Support T1 🡪 Cost T3 (c_13_) | -0.04 | [-0.18, | 0.11] | .637 | 0.02 | [-0.13, | 0.16] | .806 | 0.08 | [-0.08, | 0.24] | .307 | 0.11 | [-0.04, | 0.26] | .161 |
| Support T2 🡪 Cost T3 (c_23_) | 0.18 | [0.03, | 0.33] | .019 | 0.09 | [-0.03, | 0.22] | .153 | -0.01 | [-0.16, | 0.14] | .891 | -0.04 | [-0.20, | 0.12] | .657 |
| Cost T1 🡪 Support T2 (d_12_) | 0.09 | [0.00, | 0.17] | .041 | -0.04 | [-0.15, | 0.06] | .412 | 0.06 | [-0.02, | 0.15] | .136 | -0.06 | [-0.15, | 0.03] | .163 |

*Note.* STV = subjective task value

**Table F4**

*Analyses With the Subsample of Students With Data Available for at Least Two Time Points: Fall 2020 Data (n = 388):*

|  | Most Difficult Course | | | | | | | | Most Important Course | | | | | | | |
| --- | --- | --- | --- | --- | --- | --- | --- | --- | --- | --- | --- | --- | --- | --- | --- | --- |
|  | Peer support | | | | Faculty support | | | | Peer support | | | | Faculty support | | | |
|  | β | 95% CI | | *p* | β | 95% CI | | *p* | β | 95% CI | | *p* | β | 95% CI | | *p* |
| Expectancy |  |  |  |  |  |  |  |  |  |  |  |  |  |  |  |  |
| Stability coefficients |  |  |  |  |  |  |  |  |  |  |  |  |  |  |  |  |
| Support T1 🡪 Support T2 (a_12_) | 0.70 | [0.63, | 0.77] | <.001 | 0.62 | [0.53, | 0.72] | <.001 | 0.72 | [0.65, | 0.79] | <.001 | 0.64 | [0.56, | 0.73] | <.001 |
| Expectancy T1 🡪 Expectancy T2 (b_12_) | 0.27 | [0.15, | 0.39] | <.001 | 0.23 | [0.10, | 0.36] | <.001 | 0.41 | [0.32, | 0.50] | <.001 | 0.39 | [0.30, | 0.48] | <.001 |
| Expectancy T1 🡪 Expectancy T3 (b_13_) | 0.42 | [0.32, | 0.53] | <.001 | 0.41 | [0.31, | 0.52] | <.001 | 0.53 | [0.42, | 0.64] | <.001 | 0.55 | [0.44, | 0.66] | <.001 |
| Expectancy T2 🡪 Expectancy T3 (b_23_) | 0.34 | [0.24, | 0.44] | <.001 | 0.32 | [0.21, | 0.42] | <.001 | 0.18 | [0.07, | 0.29] | .001 | 0.18 | [0.07, | 0.29] | .002 |
| Cross-lagged coefficients |  |  |  |  |  |  |  |  |  |  |  |  |  |  |  |  |
| Support T1 🡪 Expectancy T2 (c_12_) | 0.07 | [-0.04, | 0.19] | .224 | 0.16 | [0.04, | 0.29] | .010 | 0.02 | [-0.09, | 0.12] | .753 | 0.14 | [0.04, | 0.24] | .006 |
| Support T1 🡪 Expectancy T3 (c_13_) | -0.06 | [-0.20, | 0.09] | .437 | 0.06 | [-0.06, | 0.18] | .299 | 0.14 | [-0.01, | 0.29] | .058 | -0.05 | [-0.19, | 0.09] | .462 |
| Support T2 🡪 Expectancy T3 (c_23_) | 0.08 | [-0.07, | 0.23] | .312 | 0.04 | [-0.08, | 0.16] | .483 | -0.12 | [-0.26, | 0.03] | .121 | 0.00 | [-0.13, | 0.13] | .976 |
| Expectancy T1 🡪 Support T2 (d_12_) | 0.02 | [-0.05, | 0.09] | .593 | 0.05 | [-0.06, | 0.15] | .386 | -0.01 | [-0.09, | 0.07] | .752 | 0.03 | [-0.05, | 0.12] | .446 |
| Positive STV |  |  |  |  |  |  |  |  |  |  |  |  |  |  |  |  |
| Stability coefficients |  |  |  |  |  |  |  |  |  |  |  |  |  |  |  |  |
| Support T1 🡪 Support T2 (a_12_) | 0.71 | [0.65, | 0.78] | <.001 | 0.63 | [0.54, | 0.72] | <.001 | 0.72 | [0.65, | 0.79] | <.001 | 0.65 | [0.56, | 0.74] | <.001 |
| Positive STV T1 🡪 Positive STV T2 (b_12_) | 0.60 | [0.52, | 0.68] | <.001 | 0.59 | [0.51, | 0.68] | <.001 | 0.61 | [0.53, | 0.69] | <.001 | 0.59 | [0.51, | 0.68] | <.001 |
| Positive STV T1 🡪 Positive STV T3 (b_13_) | 0.42 | [0.30, | 0.54] | <.001 | 0.41 | [0.29, | 0.53] | <.001 | 0.60 | [0.49, | 0.71] | <.001 | 0.60 | [0.49, | 0.71] | <.001 |
| Positive STV T2 🡪 Positive STV T3 (b_23_) | 0.38 | [0.27, | 0.49] | <.001 | 0.37 | [0.26, | 0.48] | <.001 | 0.24 | [0.14, | 0.34] | <.001 | 0.24 | [0.14, | 0.35] | <.001 |
| Cross-lagged coefficients |  |  |  |  |  |  |  |  |  |  |  |  |  |  |  |  |
| Support T1 🡪 Positive STV T2 (c_12_) | -0.01 | [-0.10, | 0.08] | .768 | 0.04 | [-0.05, | 0.12] | .421 | 0.05 | [-0.04, | 0.14] | .271 | 0.08 | [-0.01, | 0.17] | .065 |
| Support T1 🡪 Positive STV T3 (c_13_) | -0.09 | [-0.20, | 0.03] | .162 | 0.01 | [-0.10, | 0.13] | .825 | 0.00 | [-0.14, | 0.14] | 1.000 | -0.01 | [-0.13, | 0.10] | .804 |
| Support T2 🡪 Positive STV T3 (c_23_) | 0.07 | [-0.05, | 0.19] | .267 | 0.06 | [-0.06, | 0.17] | .351 | 0.03 | [-0.11, | 0.16] | .721 | -0.01 | [-0.12, | 0.09] | .813 |
| Positive STV T1 🡪 Support T2 (d_12_) | -0.04 | [-0.12, | 0.03] | .253 | 0.04 | [-0.05, | 0.12] | .384 | 0.00 | [-0.08, | 0.09] | .917 | -0.01 | [-0.09, | 0.08] | .896 |
| Cost |  |  |  |  |  |  |  |  |  |  |  |  |  |  |  |  |
| Stability coefficients |  |  |  |  |  |  |  |  |  |  |  |  |  |  |  |  |
| Support T1 🡪 Support T2 (a_12_) | 0.71 | [0.64, | 0.78] | <.001 | 0.64 | [0.56, | 0.72] | <.001 | 0.72 | [0.64, | 0.79] | <.001 | 0.65 | [0.57, | 0.73] | <.001 |
| Cost T1 🡪 Cost T2(b_12_) | 0.37 | [0.27, | 0.47] | <.001 | 0.37 | [0.27, | 0.48] | <.001 | 0.31 | [0.20, | 0.42] | <.001 | 0.31 | [0.21, | 0.42] | <.001 |
| Cost T1 🡪 Cost T3 (b_13_) | 0.41 | [0.31, | 0.52] | <.001 | 0.41 | [0.30, | 0.52] | <.001 | 0.56 | [0.46, | 0.66] | <.001 | 0.56 | [0.46, | 0.66] | <.001 |
| Cost T2 🡪 Cost T3 (b_23_) | 0.31 | [0.20, | 0.42] | <.001 | 0.31 | [0.20, | 0.43] | <.001 | 0.24 | [0.14, | 0.35] | <.001 | 0.25 | [0.14, | 0.35] | <.001 |
| Cross-lagged coefficients |  |  |  |  |  |  |  |  |  |  |  |  |  |  |  |  |
| Support T1 🡪 Cost T2 (c_12_) | -0.01 | [-0.11, | 0.10] | .895 | 0.01 | [-0.10, | 0.12] | .878 | 0.01 | [-0.10, | 0.11] | .919 | 0.00 | [-0.11, | 0.10] | .947 |
| Support T1 🡪 Cost T3 (c_13_) | 0.07 | [-0.07, | 0.21] | .315 | -0.05 | [-0.18, | 0.07] | .409 | -0.07 | [-0.20, | 0.06] | .312 | -0.06 | [-0.18, | 0.07] | .371 |
| Support T2 🡪 Cost T3 (c_23_) | -0.13 | [-0.27, | 0.02] | .081 | 0.00 | [-0.13, | 0.14] | .950 | 0.13 | [0.01, | 0.24] | .036 | 0.04 | [-0.08, | 0.16] | .532 |
| Cost T1 🡪 Support T2 (d_12_) | -0.01 | [-0.09, | 0.07] | .803 | -0.01 | [-0.09, | 0.08] | .869 | 0.05 | [-0.02, | 0.13] | .164 | 0.01 | [-0.08, | 0.09] | .878 |

*Note.* STV = subjective task value

**Appendix G**

**Tables with Covariates (Full Sample)**

**Table G1**

*Coefficients from Path Models for Expectancy in the Fall 2019 Data*

|  | Most difficult course | | | | | | | | Most important course | | | | | | | |
| --- | --- | --- | --- | --- | --- | --- | --- | --- | --- | --- | --- | --- | --- | --- | --- | --- |
|  | Peer support | | | | Faculty support | | | | Peer support | | | | Faculty support | | | |
|  | β | 95% CI | | *p* | β | 95% CI | | *p* | β | 95% CI | | *p* | β | 95% CI | | *p* |
| Expectancy T2 on |  |  |  |  |  |  |  |  |  |  |  |  |  |  |  |  |
| Expectancy T1 | 0.28 | [0.14, | 0.42] | <.001 | 0.25 | [0.11, | 0.39] | <.001 | 0.28 | [0.17, | 0.40] | <.001 | 0.27 | [0.16, | 0.38] | <.001 |
| Support T1 | 0.01 | [-0.12, | 0.13] | .924 | 0.10 | [-0.03, | 0.23] | .124 | -0.01 | [-0.12, | 0.11] | .895 | 0.13 | [0.00, | 0.25] | .045 |
| GPA | 0.18 | [0.04, | 0.32] | .015 | 0.18 | [0.04, | 0.32] | .012 | 0.14 | [-0.01, | 0.28] | .060 | 0.14 | [-0.01, | 0.28] | .059 |
| Gender (1 = male) | 0.32 | [0.09, | 0.56] | .007 | 0.30 | [0.06, | 0.53] | .014 | 0.13 | [-0.10, | 0.36] | .274 | 0.09 | [-0.14, | 0.32] | .441 |
| Ethnicity: Hispanic | 0.03 | [-0.24, | 0.29] | .842 | 0.03 | [-0.24, | 0.29] | .851 | 0.20 | [-0.07, | 0.46] | .146 | 0.18 | [-0.09, | 0.44] | .196 |
| Ethnicity: White | 0.28 | [-0.05, | 0.62] | .096 | 0.27 | [-0.06, | 0.61] | .111 | 0.05 | [-0.36, | 0.45] | .818 | 0.04 | [-0.36, | 0.44] | .841 |
| Ethnicity: Other | 0.85 | [0.44, | 1.25] | <.001 | 0.87 | [0.49, | 1.24] | <.001 | 0.04 | [-0.85, | 0.94] | .923 | 0.10 | [-0.79, | 0.98] | .831 |
| FG student | -0.06 | [-0.28, | 0.17] | .618 | -0.06 | [-0.28, | 0.17] | .630 | -0.06 | [-0.31, | 0.18] | .615 | -0.07 | [-0.31, | 0.17] | .577 |
| Transfer student | 0.27 | [-0.08, | 0.62] | .129 | 0.29 | [-0.05, | 0.63] | .099 | 0.41 | [0.06, | 0.76] | .021 | 0.42 | [0.08, | 0.75] | .015 |
| Week 7 | 0.22 | [-0.07, | 0.51] | .129 | 0.23 | [-0.05, | 0.52] | .111 | 0.03 | [-0.31, | 0.36] | .871 | 0.02 | [-0.30, | 0.35] | .884 |
| Week 8 | 0.19 | [-0.14, | 0.52] | .252 | 0.20 | [-0.14, | 0.53] | .248 | -0.27 | [-0.62, | 0.09] | .137 | -0.26 | [-0.61, | 0.09] | .146 |
| Week 9 | 0.04 | [-0.30, | 0.37] | .830 | 0.04 | [-0.28, | 0.36] | .787 | 0.05 | [-0.22, | 0.33] | .705 | 0.07 | [-0.21, | 0.34] | .644 |
| Support T2 on |  |  |  |  |  |  |  |  |  |  |  |  |  |  |  |  |
| Support T1 | 0.71 | [0.63, | 0.80] | <.001 | 0.63 | [0.53, | 0.72] | <.001 | 0.71 | [0.62, | 0.79] | <.001 | 0.64 | [0.55, | 0.74] | <.001 |
| Expectancy T1 | 0.02 | [-0.07, | 0.12] | .655 | 0.11 | [0.02, | 0.21] | .024 | 0.02 | [-0.06, | 0.11] | .579 | 0.09 | [-0.01, | 0.18] | .079 |
| GPA | -0.04 | [-0.14, | 0.07] | .498 | -0.01 | [-0.11, | 0.10] | .924 | -0.04 | [-0.14, | 0.06] | .455 | -0.01 | [-0.12, | 0.10] | .841 |
| Gender (1 = male) | 0.03 | [-0.15, | 0.22] | .738 | -0.08 | [-0.26, | 0.11] | .435 | 0.02 | [-0.16, | 0.20] | .834 | -0.08 | [-0.27, | 0.11] | .405 |
| Ethnicity: Hispanic | -0.14 | [-0.33, | 0.06] | .169 | -0.12 | [-0.32, | 0.08] | .229 | -0.10 | [-0.29, | 0.10] | .323 | -0.08 | [-0.29, | 0.12] | .429 |
| Ethnicity: White | 0.05 | [-0.22, | 0.31] | .736 | 0.13 | [-0.16, | 0.41] | .389 | 0.08 | [-0.19, | 0.35] | .566 | 0.14 | [-0.17, | 0.44] | .381 |
| Ethnicity: Other | -0.48 | [-0.78, | -0.18] | .002 | -0.04 | [-0.45, | 0.37] | .857 | -0.48 | [-0.80, | -0.16] | .003 | -0.05 | [-0.44, | 0.34] | .801 |
| FG student | 0.13 | [-0.04, | 0.31] | .135 | 0.01 | [-0.19, | 0.20] | .958 | 0.14 | [-0.04, | 0.31] | .124 | 0.00 | [-0.19, | 0.19] | .989 |
| Transfer student | -0.08 | [-0.33, | 0.18] | .562 | 0.07 | [-0.21, | 0.34] | .639 | -0.07 | [-0.31, | 0.17] | .555 | 0.06 | [-0.22, | 0.34] | .691 |
| Week 7 | -0.01 | [-0.24, | 0.21] | .912 | -0.05 | [-0.30, | 0.20] | .688 | -0.05 | [-0.28, | 0.19] | .696 | 0.07 | [-0.19, | 0.34] | .581 |
| Week 8 | 0.03 | [-0.24, | 0.29] | .854 | 0.15 | [-0.14, | 0.43] | .311 | 0.13 | [-0.12, | 0.38] | .308 | 0.26 | [0.00, | 0.53] | .054 |
| Week 9 | 0.03 | [-0.25, | 0.30] | .841 | 0.13 | [-0.14, | 0.40] | .334 | -0.12 | [-0.36, | 0.13] | .357 | 0.09 | [-0.18, | 0.35] | .531 |
| Support T3 on |  |  |  |  |  |  |  |  |  |  |  |  |  |  |  |  |
| Support T2 | 0.25 | [0.11, | 0.38] | <.001 | 0.25 | [0.10, | 0.39] | .001 | 0.27 | [0.11, | 0.43] | .001 | 0.26 | [0.11, | 0.41] | .001 |
| Support T1 | 0.29 | [0.15, | 0.42] | <.001 | 0.28 | [0.14, | 0.42] | <.001 | 0.27 | [0.16, | 0.38] | <.001 | 0.27 | [0.16, | 0.38] | <.001 |
| Expectancy T2 | 0.10 | [-0.07, | 0.27] | .244 | 0.03 | [-0.13, | 0.19] | .703 | 0.00 | [-0.16, | 0.17] | .983 | -0.13 | [-0.28, | 0.02] | .097 |
| Expectancy T1 | -0.11 | [-0.27, | 0.05] | .189 | 0.01 | [-0.13, | 0.14] | .939 | 0.06 | [-0.10, | 0.22] | .470 | 0.20 | [0.06, | 0.34] | .005 |
| GPA | 0.06 | [-0.10, | 0.21] | .468 | 0.05 | [-0.10, | 0.21] | .498 | 0.01 | [-0.11, | 0.14] | .848 | 0.01 | [-0.11, | 0.14] | .823 |
| Gender (1 = male) | 0.06 | [-0.18, | 0.29] | .642 | 0.06 | [-0.18, | 0.29] | .640 | 0.24 | [0.03, | 0.45] | .023 | 0.22 | [0.01, | 0.43] | .042 |
| Ethnicity: Hispanic | 0.18 | [-0.06, | 0.43] | .134 | 0.16 | [-0.09, | 0.41] | .207 | 0.14 | [-0.10, | 0.38] | .254 | 0.12 | [-0.12, | 0.37] | .313 |
| Ethnicity: White | 0.08 | [-0.29, | 0.45] | .678 | 0.08 | [-0.29, | 0.44] | .681 | 0.26 | [-0.06, | 0.58] | .106 | 0.26 | [-0.05, | 0.56] | .098 |
| Ethnicity: Other | 0.09 | [-0.37, | 0.54] | .714 | 0.02 | [-0.42, | 0.47] | .917 | 0.15 | [-0.70, | 1.00] | .732 | 0.10 | [-0.74, | 0.94] | .812 |
| FG student | -0.17 | [-0.40, | 0.06] | .139 | -0.16 | [-0.39, | 0.06] | .160 | 0.16 | [-0.06, | 0.38] | .159 | 0.16 | [-0.07, | 0.39] | .161 |
| Transfer student | 0.10 | [-0.25, | 0.44] | .592 | 0.10 | [-0.26, | 0.45] | .589 | 0.07 | [-0.25, | 0.40] | .656 | 0.09 | [-0.23, | 0.42] | .572 |
| Week 7 | 0.34 | [0.06, | 0.63] | .018 | 0.34 | [0.05, | 0.63] | .020 | 0.13 | [-0.21, | 0.46] | .455 | 0.14 | [-0.19, | 0.46] | .415 |
| Week 8 | -0.04 | [-0.37, | 0.29] | .802 | -0.05 | [-0.39, | 0.29] | .779 | 0.16 | [-0.17, | 0.49] | .332 | 0.20 | [-0.12, | 0.52] | .219 |
| Week 9 | 0.06 | [-0.30, | 0.41] | .761 | 0.05 | [-0.30, | 0.40] | .785 | 0.14 | [-0.17, | 0.44] | .369 | 0.17 | [-0.13, | 0.47] | .260 |

**Table G2**

*Coefficients from Path Models for Positive STV in the Fall 2019 Data*

|  | Most difficult course | | | | | | | | Most important course | | | | | | | |
| --- | --- | --- | --- | --- | --- | --- | --- | --- | --- | --- | --- | --- | --- | --- | --- | --- |
|  | Peer support | | | | Faculty support | | | | Peer support | | | | Faculty support | | | |
|  | β | 95% CI | | *p* | β | 95% CI | | *p* | β | 95% CI | | *p* | β | 95% CI | | *p* |
| Positive STV T2 on |  |  |  |  |  |  |  |  |  |  |  |  |  |  |  |  |
| Positive STV T1 | 0.56 | [0.45, | 0.67] | <.001 | 0.55 | [0.44, | 0.67] | <.001 | 0.52 | [0.42, | 0.62] | <.001 | 0.51 | [0.41, | 0.61] | <.001 |
| Support T1 | 0.04 | [-0.07, | 0.16] | .444 | 0.07 | [-0.05, | 0.20] | .256 | 0.10 | [-0.02, | 0.22] | .106 | 0.11 | [0.00, | 0.22] | .059 |
| GPA | 0.08 | [-0.06, | 0.21] | .253 | 0.08 | [-0.06, | 0.21] | .257 | 0.08 | [-0.05, | 0.22] | .227 | 0.08 | [-0.05, | 0.22] | .227 |
| Gender (1 = male) | 0.10 | [-0.11, | 0.31] | .340 | 0.10 | [-0.12, | 0.31] | .385 | -0.11 | [-0.34, | 0.11] | .330 | -0.14 | [-0.37, | 0.09] | .241 |
| Ethnicity: Hispanic | 0.06 | [-0.20, | 0.32] | .640 | 0.07 | [-0.19, | 0.33] | .598 | 0.05 | [-0.23, | 0.33] | .716 | 0.05 | [-0.22, | 0.33] | .704 |
| Ethnicity: White | 0.28 | [-0.05, | 0.61] | .098 | 0.25 | [-0.07, | 0.58] | .129 | 0.05 | [-0.32, | 0.42] | .796 | 0.03 | [-0.33, | 0.39] | .864 |
| Ethnicity: Other | -0.13 | [-0.75, | 0.49] | .672 | -0.12 | [-0.72, | 0.48] | .692 | 0.30 | [-0.15, | 0.75] | .190 | 0.29 | [-0.13, | 0.71] | .179 |
| FG student | 0.20 | [-0.03, | 0.42] | .091 | 0.19 | [-0.03, | 0.41] | .093 | 0.03 | [-0.21, | 0.27] | .826 | 0.04 | [-0.19, | 0.28] | .726 |
| Transfer student | 0.26 | [-0.08, | 0.59] | .132 | 0.26 | [-0.07, | 0.58] | .125 | 0.07 | [-0.30, | 0.43] | .724 | 0.06 | [-0.31, | 0.42] | .764 |
| Week 7 | -0.04 | [-0.27, | 0.19] | .752 | -0.02 | [-0.25, | 0.21] | .853 | 0.22 | [-0.06, | 0.49] | .121 | 0.20 | [-0.07, | 0.47] | .143 |
| Week 8 | 0.21 | [-0.08, | 0.50] | .152 | 0.22 | [-0.06, | 0.51] | .127 | 0.11 | [-0.22, | 0.44] | .529 | 0.12 | [-0.21, | 0.45] | .477 |
| Week 9 | -0.22 | [-0.52, | 0.08] | .145 | -0.20 | [-0.50, | 0.10] | .194 | 0.14 | [-0.15, | 0.43] | .352 | 0.14 | [-0.15, | 0.43] | .348 |
| Support T2 on |  |  |  |  |  |  |  |  |  |  |  |  |  |  |  |  |
| Support T1 | 0.71 | [0.62, | 0.80] | <.001 | 0.64 | [0.55, | 0.74] | <.001 | 0.69 | [0.61, | 0.78] | <.001 | 0.65 | [0.55, | 0.74] | <.001 |
| Positive STV T1 | 0.06 | [-0.03, | 0.14] | .188 | 0.04 | [-0.06, | 0.13] | .469 | 0.11 | [0.03, | 0.20] | .011 | 0.04 | [-0.06, | 0.14] | .457 |
| GPA | -0.04 | [-0.14, | 0.07] | .476 | -0.01 | [-0.11, | 0.10] | .897 | -0.04 | [-0.14, | 0.07] | .494 | -0.01 | [-0.12, | 0.10] | .906 |
| Gender (1 = male) | 0.03 | [-0.15, | 0.22] | .721 | -0.06 | [-0.25, | 0.13] | .531 | 0.02 | [-0.16, | 0.19] | .864 | -0.07 | [-0.26, | 0.12] | .473 |
| Ethnicity: Hispanic | -0.14 | [-0.33, | 0.05] | .142 | -0.09 | [-0.29, | 0.11] | .357 | -0.14 | [-0.32, | 0.05] | .160 | -0.08 | [-0.28, | 0.13] | .463 |
| Ethnicity: White | 0.06 | [-0.20, | 0.33] | .634 | 0.15 | [-0.14, | 0.43] | .308 | 0.10 | [-0.17, | 0.37] | .452 | 0.16 | [-0.14, | 0.46] | .286 |
| Ethnicity: Other | -0.49 | [-0.79, | -0.19] | .002 | -0.08 | [-0.48, | 0.32] | .689 | -0.46 | [-0.81, | -0.12] | .009 | -0.04 | [-0.44, | 0.35] | .836 |
| FG student | 0.13 | [-0.05, | 0.30] | .153 | -0.01 | [-0.21, | 0.19] | .922 | 0.14 | [-0.03, | 0.32] | .107 | -0.01 | [-0.20, | 0.18] | .927 |
| Transfer student | -0.09 | [-0.34, | 0.16] | .481 | 0.11 | [-0.17, | 0.38] | .457 | -0.08 | [-0.32, | 0.15] | .492 | 0.08 | [-0.20, | 0.36] | .585 |
| Week 7 | -0.03 | [-0.25, | 0.20] | .825 | -0.04 | [-0.29, | 0.21] | .744 | -0.06 | [-0.30, | 0.17] | .610 | 0.09 | [-0.17, | 0.35] | .497 |
| Week 8 | 0.02 | [-0.24, | 0.27] | .910 | 0.14 | [-0.14, | 0.42] | .313 | 0.09 | [-0.15, | 0.34] | .453 | 0.25 | [-0.02, | 0.53] | .073 |
| Week 9 | 0.02 | [-0.26, | 0.29] | .895 | 0.11 | [-0.16, | 0.38] | .425 | -0.14 | [-0.39, | 0.11] | .261 | 0.07 | [-0.20, | 0.34] | .594 |
| Support T3 on |  |  |  |  |  |  |  |  |  |  |  |  |  |  |  |  |
| Support T2 | 0.37 | [0.22, | 0.53] | <.001 | 0.38 | [0.22, | 0.54] | <.001 | 0.39 | [0.27, | 0.50] | <.001 | 0.38 | [0.27, | 0.50] | <.001 |
| Support T1 | 0.27 | [0.13, | 0.41] | <.001 | 0.25 | [0.09, | 0.40] | .002 | 0.41 | [0.30, | 0.52] | <.001 | 0.41 | [0.30, | 0.53] | <.001 |
| Positive STV T2 | 0.10 | [-0.05, | 0.25] | .200 | 0.02 | [-0.12, | 0.15] | .811 | 0.10 | [-0.04, | 0.23] | .156 | -0.03 | [-0.14, | 0.08] | .581 |
| Positive STV T1 | -0.07 | [-0.23, | 0.09] | .373 | 0.08 | [-0.05, | 0.22] | .224 | -0.06 | [-0.18, | 0.06] | .324 | 0.05 | [-0.06, | 0.16] | .353 |
| GPA | 0.04 | [-0.08, | 0.16] | .476 | 0.04 | [-0.08, | 0.16] | .516 | 0.03 | [-0.09, | 0.14] | .675 | 0.03 | [-0.09, | 0.15] | .658 |
| Gender (1 = male) | -0.12 | [-0.32, | 0.08] | .236 | -0.15 | [-0.35, | 0.06] | .164 | 0.16 | [-0.01, | 0.34] | .068 | 0.16 | [-0.02, | 0.33] | .080 |
| Ethnicity: Hispanic | 0.09 | [-0.15, | 0.33] | .468 | 0.06 | [-0.19, | 0.30] | .649 | 0.14 | [-0.08, | 0.36] | .199 | 0.12 | [-0.09, | 0.34] | .261 |
| Ethnicity: White | 0.14 | [-0.18, | 0.46] | .387 | 0.12 | [-0.21, | 0.44] | .477 | 0.24 | [-0.02, | 0.49] | .068 | 0.24 | [-0.01, | 0.50] | .062 |
| Ethnicity: Other | 0.20 | [-0.21, | 0.61] | .336 | 0.13 | [-0.26, | 0.53] | .501 | 0.00 | [-0.47, | 0.46] | .989 | -0.06 | [-0.53, | 0.41] | .798 |
| FG student | -0.03 | [-0.25, | 0.18] | .773 | -0.02 | [-0.24, | 0.19] | .832 | -0.12 | [-0.30, | 0.06] | .183 | -0.11 | [-0.29, | 0.07] | .225 |
| Transfer student | -0.02 | [-0.34, | 0.29] | .895 | -0.01 | [-0.33, | 0.31] | .940 | 0.16 | [-0.12, | 0.45] | .250 | 0.17 | [-0.11, | 0.45] | .233 |
| Week 7 | 0.15 | [-0.12, | 0.42] | .285 | 0.16 | [-0.11, | 0.43] | .247 | 0.00 | [-0.26, | 0.26] | .991 | -0.01 | [-0.27, | 0.25] | .948 |
| Week 8 | 0.15 | [-0.13, | 0.43] | .290 | 0.16 | [-0.12, | 0.43] | .259 | 0.07 | [-0.20, | 0.34] | .616 | 0.08 | [-0.20, | 0.36] | .570 |
| Week 9 | 0.12 | [-0.22, | 0.45] | .490 | 0.14 | [-0.18, | 0.46] | .391 | 0.16 | [-0.09, | 0.41] | .215 | 0.15 | [-0.10, | 0.40] | .247 |

*Note.* GPA = grade point average; FG = first-generation; STV = subjective task value.

**Table G3**

*Coefficients from Path Models for Cost in the Fall 2019 Data*

|  | Most difficult course | | | | | | | | Most important course | | | | | | | |
| --- | --- | --- | --- | --- | --- | --- | --- | --- | --- | --- | --- | --- | --- | --- | --- | --- |
|  | Peer support | | | | Faculty support | | | | Peer support | | | | Faculty support | | | |
|  | β | 95% CI | | *p* | β | 95% CI | | *p* | β | 95% CI | | *p* | β | 95% CI | | *p* |
| Cost T2 on |  |  |  |  |  |  |  |  |  |  |  |  |  |  |  |  |
| Cost T1 | 0.42 | [0.29, | 0.54] | <.001 | 0.41 | [0.29, | 0.54] | <.001 | 0.53 | [0.41, | 0.65] | <.001 | 0.53 | [0.41, | 0.65] | <.001 |
| Support T1 | -0.02 | [-0.13, | 0.09] | .702 | -0.04 | [-0.15, | 0.07] | .496 | 0.01 | [-0.09, | 0.11] | .877 | 0.01 | [-0.10, | 0.11] | .884 |
| GPA | -0.10 | [-0.25, | 0.04] | .159 | -0.11 | [-0.25, | 0.04] | .153 | -0.03 | [-0.17, | 0.12] | .692 | -0.03 | [-0.18, | 0.11] | .648 |
| Gender (1 = male) | -0.13 | [-0.37, | 0.11] | .288 | -0.10 | [-0.34, | 0.14] | .421 | 0.12 | [-0.11, | 0.35] | .297 | 0.13 | [-0.10, | 0.36] | .279 |
| Ethnicity: Hispanic | -0.29 | [-0.56, | -0.02] | .034 | -0.29 | [-0.56, | -0.02] | .038 | -0.26 | [-0.51, | -0.01] | .041 | -0.25 | [-0.50, | -0.01] | .046 |
| Ethnicity: White | 0.17 | [-0.15, | 0.49] | .297 | 0.15 | [-0.16, | 0.47] | .346 | 0.28 | [-0.02, | 0.57] | .065 | 0.29 | [-0.01, | 0.58] | .058 |
| Ethnicity: Other | -0.40 | [-1.01, | 0.22] | .209 | -0.43 | [-1.07, | 0.20] | .183 | -0.11 | [-0.72, | 0.51] | .732 | -0.11 | [-0.73, | 0.50] | .713 |
| FG student | 0.12 | [-0.10, | 0.34] | .284 | 0.11 | [-0.11, | 0.32] | .349 | 0.20 | [-0.01, | 0.42] | .065 | 0.20 | [-0.02, | 0.41] | .072 |
| Transfer student | -0.03 | [-0.37, | 0.32] | .883 | -0.03 | [-0.37, | 0.32] | .878 | -0.08 | [-0.41, | 0.25] | .629 | -0.08 | [-0.41, | 0.25] | .640 |
| Week 7 | -0.15 | [-0.45, | 0.15] | .335 | -0.16 | [-0.46, | 0.14] | .291 | 0.17 | [-0.10, | 0.45] | .223 | 0.17 | [-0.11, | 0.45] | .232 |
| Week 8 | 0.34 | [0.05, | 0.63] | .021 | 0.33 | [0.04, | 0.62] | .025 | 0.29 | [-0.01, | 0.59] | .062 | 0.28 | [-0.02, | 0.58] | .067 |
| Week 9 | 0.12 | [-0.20, | 0.45] | .459 | 0.11 | [-0.21, | 0.43] | .503 | 0.16 | [-0.12, | 0.44] | .258 | 0.16 | [-0.12, | 0.44] | .271 |
| Support T2 on |  |  |  |  |  |  |  |  |  |  |  |  |  |  |  |  |
| Support T1 | 0.72 | [0.64, | 0.81] | <.001 | 0.65 | [0.56, | 0.74] | <.001 | 0.71 | [0.63, | 0.80] | <.001 | 0.65 | [0.56, | 0.74] | <.001 |
| Cost T1 | 0.09 | [0.00, | 0.17] | .041 | -0.04 | [-0.15, | 0.06] | .406 | 0.06 | [-0.02, | 0.14] | .143 | -0.07 | [-0.16, | 0.02] | .149 |
| GPA | -0.04 | [-0.14, | 0.06] | .425 | 0.00 | [-0.11, | 0.11] | .962 | -0.03 | [-0.14, | 0.07] | .521 | -0.01 | [-0.12, | 0.10] | .816 |
| Gender (1 = male) | 0.04 | [-0.14, | 0.23] | .640 | -0.07 | [-0.26, | 0.12] | .493 | 0.02 | [-0.16, | 0.20] | .819 | -0.08 | [-0.27, | 0.12] | .442 |
| Ethnicity: Hispanic | -0.13 | [-0.32, | 0.06] | .180 | -0.10 | [-0.30, | 0.10] | .345 | -0.10 | [-0.29, | 0.09] | .312 | -0.06 | [-0.26, | 0.14] | .546 |
| Ethnicity: White | 0.08 | [-0.18, | 0.34] | .546 | 0.14 | [-0.15, | 0.42] | .353 | 0.10 | [-0.16, | 0.36] | .464 | 0.16 | [-0.14, | 0.45] | .306 |
| Ethnicity: Other | -0.48 | [-0.78, | -0.18] | .002 | -0.08 | [-0.46, | 0.30] | .677 | -0.45 | [-0.77, | -0.14] | .005 | -0.08 | [-0.46, | 0.30] | .680 |
| FG student | 0.12 | [-0.06, | 0.29] | .190 | -0.01 | [-0.20, | 0.19] | .962 | 0.12 | [-0.06, | 0.30] | .184 | 0.00 | [-0.19, | 0.19] | .985 |
| Transfer student | -0.05 | [-0.30, | 0.21] | .725 | 0.12 | [-0.16, | 0.39] | .406 | -0.06 | [-0.30, | 0.19] | .654 | 0.07 | [-0.21, | 0.36] | .610 |
| Week 7 | 0.01 | [-0.22, | 0.24] | .913 | -0.05 | [-0.30, | 0.21] | .725 | -0.05 | [-0.29, | 0.19] | .675 | 0.10 | [-0.16, | 0.36] | .441 |
| Week 8 | 0.03 | [-0.22, | 0.28] | .820 | 0.15 | [-0.14, | 0.43] | .312 | 0.13 | [-0.11, | 0.38] | .286 | 0.29 | [0.02, | 0.56] | .037 |
| Week 9 | 0.01 | [-0.27, | 0.28] | .970 | 0.11 | [-0.15, | 0.38] | .404 | -0.10 | [-0.35, | 0.14] | .406 | 0.08 | [-0.19, | 0.35] | .568 |
| Support T3 on |  |  |  |  |  |  |  |  |  |  |  |  |  |  |  |  |
| Support T2 | 0.42 | [0.29, | 0.56] | <.001 | 0.41 | [0.27, | 0.55] | <.001 | 0.37 | [0.24, | 0.50] | <.001 | 0.39 | [0.26, | 0.51] | <.001 |
| Support T1 | 0.26 | [0.13, | 0.39] | <.001 | 0.26 | [0.13, | 0.39] | <.001 | 0.31 | [0.20, | 0.42] | <.001 | 0.31 | [0.20, | 0.42] | <.001 |
| Cost T2 | -0.03 | [-0.18, | 0.11] | .640 | 0.02 | [-0.13, | 0.16] | .804 | 0.08 | [-0.08, | 0.24] | .307 | 0.11 | [-0.04, | 0.26] | .160 |
| Cost T1 | 0.18 | [0.03, | 0.33] | .019 | 0.09 | [-0.04, | 0.22] | .155 | -0.01 | [-0.16, | 0.14] | .892 | -0.04 | [-0.20, | 0.12] | .654 |
| GPA | -0.14 | [-0.28, | 0.00] | .052 | -0.14 | [-0.28, | 0.00] | .056 | 0.07 | [-0.06, | 0.21] | .284 | 0.08 | [-0.06, | 0.21] | .252 |
| Gender (1 = male) | 0.03 | [-0.17, | 0.23] | .795 | 0.00 | [-0.20, | 0.21] | .967 | 0.03 | [-0.18, | 0.24] | .778 | 0.02 | [-0.19, | 0.24] | .834 |
| Ethnicity: Hispanic | -0.22 | [-0.43, | 0.00] | .046 | -0.21 | [-0.43, | 0.01] | .067 | 0.01 | [-0.23, | 0.25] | .939 | 0.01 | [-0.23, | 0.25] | .929 |
| Ethnicity: White | 0.06 | [-0.31, | 0.43] | .738 | 0.04 | [-0.33, | 0.41] | .831 | -0.10 | [-0.38, | 0.18] | .492 | -0.12 | [-0.40, | 0.16] | .408 |
| Ethnicity: Other | -0.07 | [-0.57, | 0.44] | .795 | -0.06 | [-0.57, | 0.45] | .824 | 0.24 | [-0.11, | 0.60] | .180 | 0.20 | [-0.17, | 0.58] | .290 |
| FG student | 0.15 | [-0.05, | 0.36] | .147 | 0.17 | [-0.04, | 0.37] | .115 | -0.04 | [-0.25, | 0.17] | .700 | -0.03 | [-0.23, | 0.19] | .817 |
| Transfer student | -0.03 | [-0.31, | 0.26] | .842 | -0.04 | [-0.34, | 0.25] | .765 | 0.14 | [-0.13, | 0.42] | .312 | 0.13 | [-0.15, | 0.40] | .362 |
| Week 7 | -0.23 | [-0.50, | 0.03] | .087 | -0.20 | [-0.47, | 0.07] | .141 | 0.10 | [-0.20, | 0.40] | .498 | 0.07 | [-0.23, | 0.37] | .646 |
| Week 8 | -0.14 | [-0.42, | 0.14] | .321 | -0.12 | [-0.40, | 0.16] | .397 | -0.15 | [-0.46, | 0.17] | .358 | -0.17 | [-0.49, | 0.14] | .277 |
| Week 9 | 0.24 | [-0.07, | 0.55] | .129 | 0.29 | [-0.02, | 0.60] | .068 | 0.00 | [-0.31, | 0.30] | .979 | -0.04 | [-0.35, | 0.27] | .800 |

*Note.* GPA = grade point average; FG = first-generation.

**Table G4**

*Coefficients from Path Models for Expectancy in the Fall 2020 Data*

|  | Most difficult course | | | | | | | | Most important course | | | | | | | |
| --- | --- | --- | --- | --- | --- | --- | --- | --- | --- | --- | --- | --- | --- | --- | --- | --- |
|  | Peer support | | | | Faculty support | | | | Peer support | | | | Faculty support | | | |
|  | β | 95% CI | | *p* | β | 95% CI | | *p* | β | 95% CI | | *p* | β | 95% CI | | *p* |
| Expectancy T2 on |  |  |  |  |  |  |  |  |  |  |  |  |  |  |  |  |
| Expectancy T1 | 0.27 | [0.15, | 0.38] | <.001 | 0.23 | [0.10, | 0.35] | <.001 | 0.41 | [0.32, | 0.50] | <.001 | 0.39 | [0.30, | 0.48] | <.001 |
| Support T1 | 0.08 | [-0.04, | 0.20] | .185 | 0.17 | [0.04, | 0.30] | .008 | 0.02 | [-0.09, | 0.13] | .693 | 0.15 | [0.05, | 0.25] | .005 |
| GPA | 0.08 | [-0.02, | 0.18] | .096 | 0.07 | [-0.02, | 0.17] | .127 | -0.07 | [-0.15, | 0.01] | .081 | -0.08 | [-0.16, | 0.00] | .056 |
| Gender (1 = male) | -0.01 | [-0.23, | 0.21] | .936 | -0.04 | [-0.26, | 0.18] | .738 | 0.12 | [-0.08, | 0.32] | .252 | 0.08 | [-0.12, | 0.28] | .417 |
| Ethnicity: Hispanic | -0.10 | [-0.33, | 0.13] | .385 | -0.10 | [-0.33, | 0.12] | .369 | 0.10 | [-0.13, | 0.33] | .390 | 0.11 | [-0.11, | 0.33] | .337 |
| Ethnicity: White | 0.03 | [-0.28, | 0.33] | .870 | -0.02 | [-0.32, | 0.28] | .891 | 0.16 | [-0.11, | 0.43] | .252 | 0.13 | [-0.14, | 0.40] | .338 |
| Ethnicity: Other | 0.15 | [-0.38, | 0.67] | .583 | 0.19 | [-0.34, | 0.73] | .475 | -0.29 | [-0.64, | 0.05] | .091 | -0.28 | [-0.63, | 0.08] | .124 |
| FG student | -0.01 | [-0.22, | 0.20] | .938 | -0.01 | [-0.21, | 0.20] | .953 | -0.11 | [-0.31, | 0.09] | .299 | -0.11 | [-0.30, | 0.09] | .281 |
| Transfer student | 0.01 | [-0.23, | 0.26] | .920 | 0.02 | [-0.22, | 0.26] | .856 | -0.13 | [-0.36, | 0.10] | .268 | -0.12 | [-0.34, | 0.11] | .314 |
| Week 6 | 0.18 | [-0.68, | 1.04] | .679 | 0.11 | [-0.71, | 0.93] | .792 | -0.28 | [-1.16, | 0.61] | .539 | -0.36 | [-1.24, | 0.53] | .431 |
| Week 7 | 0.12 | [-0.14, | 0.38] | .373 | 0.11 | [-0.16, | 0.37] | .430 | 0.06 | [-0.19, | 0.30] | .650 | 0.04 | [-0.20, | 0.28] | .750 |
| Week 8 | -0.22 | [-0.53, | 0.09] | .167 | -0.27 | [-0.59, | 0.05] | .093 | 0.24 | [-0.14, | 0.62] | .218 | 0.25 | [-0.12, | 0.61] | .185 |
| Support T2 on |  |  |  |  |  |  |  |  |  |  |  |  |  |  |  |  |
| Support T1 | 0.71 | [0.64, | 0.78] | <.001 | 0.63 | [0.53, | 0.73] | <.001 | 0.72 | [0.65, | 0.79] | <.001 | 0.65 | [0.56, | 0.74] | <.001 |
| Expectancy T1 | 0.02 | [-0.05, | 0.09] | .565 | 0.05 | [-0.06, | 0.14] | .379 | -0.01 | [-0.09, | 0.07] | .767 | 0.04 | [-0.05, | 0.12] | .428 |
| GPA | -0.09 | [-0.16, | -0.01] | .026 | -0.05 | [-0.13, | 0.03] | .209 | -0.07 | [-0.15, | 0.01] | .068 | -0.04 | [-0.12, | 0.04] | .353 |
| Gender (1 = male) | 0.01 | [-0.15, | 0.18] | .886 | 0.09 | [-0.09, | 0.26] | .333 | 0.00 | [-0.17, | 0.16] | .986 | 0.11 | [-0.06, | 0.29] | .212 |
| Ethnicity: Hispanic | 0.09 | [-0.09, | 0.28] | .314 | -0.03 | [-0.23, | 0.18] | .807 | 0.09 | [-0.10, | 0.27] | .344 | -0.02 | [-0.23, | 0.19] | .846 |
| Ethnicity: White | 0.12 | [-0.10, | 0.35] | .277 | 0.08 | [-0.16, | 0.32] | .509 | 0.12 | [-0.10, | 0.34] | .295 | 0.06 | [-0.18, | 0.30] | .611 |
| Ethnicity: Other | 0.25 | [-0.21, | 0.70] | .287 | -0.15 | [-0.48, | 0.18] | .371 | 0.27 | [-0.20, | 0.73] | .258 | -0.14 | [-0.47, | 0.19] | .402 |
| FG student | 0.01 | [-0.16, | 0.17] | .908 | -0.12 | [-0.30, | 0.06] | .176 | 0.02 | [-0.14, | 0.18] | .795 | -0.12 | [-0.30, | 0.05] | .163 |
| Transfer student | 0.02 | [-0.15, | 0.19] | .805 | 0.00 | [-0.21, | 0.21] | .990 | 0.05 | [-0.13, | 0.22] | .621 | 0.04 | [-0.18, | 0.26] | .727 |
| Week 6 | -0.12 | [-0.78, | 0.54] | .724 | -0.15 | [-0.79, | 0.49] | .650 | -0.26 | [-0.66, | 0.14] | .206 | -0.72 | [-1.24, | -0.20] | .007 |
| Week 7 | -0.02 | [-0.21, | 0.16] | .800 | 0.02 | [-0.19, | 0.24] | .852 | 0.08 | [-0.15, | 0.31] | .480 | 0.09 | [-0.14, | 0.31] | .449 |
| Week 8 | 0.17 | [-0.06, | 0.40] | .153 | 0.03 | [-0.29, | 0.34] | .875 | 0.36 | [-0.01, | 0.72] | .055 | 0.00 | [-0.31, | 0.32] | .997 |
| Support T3 on |  |  |  |  |  |  |  |  |  |  |  |  |  |  |  |  |
| Support T2 | 0.43 | [0.32, | 0.53] | <.001 | 0.42 | [0.31, | 0.52] | <.001 | 0.53 | [0.42, | 0.64] | <.001 | 0.55 | [0.44, | 0.66] | <.001 |
| Support T1 | 0.34 | [0.24, | 0.44] | <.001 | 0.31 | [0.21, | 0.42] | <.001 | 0.18 | [0.07, | 0.29] | .001 | 0.18 | [0.07, | 0.29] | .002 |
| Expectancy T2 | -0.06 | [-0.20, | 0.09] | .441 | 0.06 | [-0.06, | 0.18] | .302 | 0.14 | [0.00, | 0.29] | .057 | -0.05 | [-0.19, | 0.08] | .457 |
| Expectancy T1 | 0.08 | [-0.07, | 0.23] | .316 | 0.04 | [-0.08, | 0.16] | .493 | -0.12 | [-0.26, | 0.03] | .121 | 0.00 | [-0.13, | 0.13] | .982 |
| GPA | -0.04 | [-0.13, | 0.05] | .391 | -0.03 | [-0.12, | 0.06] | .474 | -0.04 | [-0.13, | 0.05] | .356 | -0.06 | [-0.15, | 0.03] | .209 |
| Gender (1 = male) | 0.00 | [-0.20, | 0.20] | .989 | -0.02 | [-0.22, | 0.19] | .875 | -0.15 | [-0.35, | 0.05] | .139 | -0.14 | [-0.35, | 0.06] | .162 |
| Ethnicity: Hispanic | -0.06 | [-0.28, | 0.16] | .582 | -0.09 | [-0.30, | 0.13] | .433 | -0.08 | [-0.28, | 0.11] | .409 | -0.07 | [-0.26, | 0.12] | .460 |
| Ethnicity: White | -0.12 | [-0.42, | 0.17] | .411 | -0.17 | [-0.46, | 0.13] | .274 | -0.09 | [-0.40, | 0.23] | .590 | -0.07 | [-0.38, | 0.24] | .661 |
| Ethnicity: Other | 0.14 | [-0.32, | 0.59] | .562 | 0.16 | [-0.31, | 0.62] | .506 | -0.74 | [-1.47, | -0.01] | .048 | -0.69 | [-1.45, | 0.07] | .074 |
| FG student | 0.10 | [-0.11, | 0.30] | .352 | 0.10 | [-0.10, | 0.30] | .337 | 0.03 | [-0.16, | 0.22] | .739 | 0.04 | [-0.15, | 0.22] | .713 |
| Transfer student | 0.24 | [0.04, | 0.45] | .020 | 0.25 | [0.05, | 0.46] | .013 | 0.04 | [-0.19, | 0.26] | .750 | 0.05 | [-0.18, | 0.27] | .682 |
| Week 6 | 0.37 | [-0.21, | 0.94] | .210 | 0.27 | [-0.23, | 0.78] | .288 | -0.35 | [-1.02, | 0.32] | .301 | -0.37 | [-1.08, | 0.34] | .307 |
| Week 7 | 0.05 | [-0.17, | 0.27] | .649 | 0.06 | [-0.16, | 0.27] | .620 | -0.04 | [-0.29, | 0.21] | .759 | -0.03 | [-0.28, | 0.22] | .817 |
| Week 8 | 0.03 | [-0.23, | 0.29] | .834 | -0.02 | [-0.27, | 0.24] | .892 | -0.19 | [-0.53, | 0.16] | .291 | -0.14 | [-0.47, | 0.19] | .415 |

*Note.* GPA = grade point average; FG = first-generation.

**Table G5**

*Coefficients from Path Models for Positive STV in the Fall 2020 Data*

|  | Most difficult course | | | | | | | | Most important course | | | | | | | |
| --- | --- | --- | --- | --- | --- | --- | --- | --- | --- | --- | --- | --- | --- | --- | --- | --- |
|  | Peer support | | | | Faculty support | | | | Peer support | | | | Faculty support | | | |
|  | β | 95% CI | | *p* | β | 95% CI | | *p* | β | 95% CI | | *p* | β | 95% CI | | *p* |
| Positive STV T2 on |  |  |  |  |  |  |  |  |  |  |  |  |  |  |  |  |
| Positive STV T1 | 0.61 | [0.53, | 0.69] | <.001 | 0.60 | [0.52, | 0.68] | <.001 | 0.61 | [0.53, | 0.69] | <.001 | 0.59 | [0.51, | 0.68] | <.001 |
| Support T1 | -0.01 | [-0.10, | 0.08] | .883 | 0.04 | [-0.05, | 0.13] | .376 | 0.05 | [-0.03, | 0.14] | .230 | 0.08 | [0.00, | 0.17] | .055 |
| GPA | 0.00 | [-0.09, | 0.08] | .937 | 0.00 | [-0.09, | 0.08] | .935 | 0.01 | [-0.08, | 0.10] | .842 | 0.00 | [-0.08, | 0.09] | .934 |
| Gender (1 = male) | 0.12 | [-0.06, | 0.29] | .190 | 0.11 | [-0.07, | 0.28] | .232 | 0.12 | [-0.06, | 0.30] | .197 | 0.10 | [-0.08, | 0.28] | .272 |
| Ethnicity: Hispanic | -0.02 | [-0.20, | 0.16] | .827 | -0.01 | [-0.19, | 0.17] | .891 | -0.01 | [-0.21, | 0.18] | .901 | -0.02 | [-0.21, | 0.18] | .871 |
| Ethnicity: White | -0.08 | [-0.34, | 0.20] | .588 | -0.09 | [-0.36, | 0.18] | .530 | -0.18 | [-0.42, | 0.06] | .149 | -0.20 | [-0.44, | 0.04] | .106 |
| Ethnicity: Other | -0.31 | [-0.63, | 0.01] | .057 | -0.30 | [-0.62, | 0.02] | .068 | -0.38 | [-0.82, | 0.06] | .090 | -0.36 | [-0.81, | 0.09] | .114 |
| FG student | -0.06 | [-0.23, | 0.11] | .474 | -0.06 | [-0.22, | 0.11] | .495 | -0.16 | [-0.33, | 0.02] | .086 | -0.16 | [-0.34, | 0.02] | .078 |
| Transfer student | 0.07 | [-0.13, | 0.26] | .514 | 0.08 | [-0.12, | 0.27] | .436 | 0.08 | [-0.14, | 0.29] | .492 | 0.08 | [-0.14, | 0.29] | .488 |
| Week 6 | 0.03 | [-0.50, | 0.56] | .909 | 0.01 | [-0.52, | 0.54] | .977 | -0.87 | [-1.59, | -0.16] | .016 | -0.92 | [-1.61, | -0.22] | .010 |
| Week 7 | -0.26 | [-0.48, | -0.03] | .028 | -0.26 | [-0.48, | -0.03] | .027 | 0.08 | [-0.15, | 0.30] | .494 | 0.07 | [-0.15, | 0.30] | .528 |
| Week 8 | -0.33 | [-0.59, | -0.06] | .016 | -0.33 | [-0.60, | -0.07] | .015 | -0.03 | [-0.33, | 0.27] | .849 | -0.03 | [-0.33, | 0.26] | .830 |
| Support T2 on |  |  |  |  |  |  |  |  |  |  |  |  |  |  |  |  |
| Support T1 | 0.72 | [0.65, | 0.79] | <.001 | 0.64 | [0.55, | 0.73] | <.001 | 0.72 | [0.65, | 0.79] | <.001 | 0.66 | [0.57, | 0.75] | <.001 |
| Positive STV T1 | -0.04 | [-0.11, | 0.04] | .326 | 0.04 | [-0.04, | 0.13] | .344 | 0.01 | [-0.07, | 0.09] | .860 | 0.00 | [-0.09, | 0.08] | .951 |
| GPA | -0.09 | [-0.16, | -0.01] | .032 | -0.05 | [-0.12, | 0.03] | .236 | -0.08 | [-0.15, | 0.00] | .054 | -0.04 | [-0.12, | 0.04] | .323 |
| Gender (1 = male) | 0.02 | [-0.15, | 0.18] | .858 | 0.09 | [-0.09, | 0.26] | .325 | 0.00 | [-0.16, | 0.16] | .993 | 0.11 | [-0.07, | 0.28] | .229 |
| Ethnicity: Hispanic | 0.10 | [-0.08, | 0.28] | .279 | -0.03 | [-0.23, | 0.17] | .775 | 0.08 | [-0.10, | 0.27] | .378 | -0.02 | [-0.23, | 0.20] | .862 |
| Ethnicity: White | 0.12 | [-0.11, | 0.35] | .310 | 0.09 | [-0.15, | 0.33] | .458 | 0.11 | [-0.11, | 0.33] | .342 | 0.07 | [-0.17, | 0.31] | .557 |
| Ethnicity: Other | 0.25 | [-0.21, | 0.71] | .283 | -0.12 | [-0.45, | 0.20] | .459 | 0.26 | [-0.21, | 0.72] | .276 | -0.13 | [-0.47, | 0.21] | .466 |
| FG student | 0.01 | [-0.15, | 0.18] | .869 | -0.12 | [-0.30, | 0.05] | .167 | 0.03 | [-0.14, | 0.19] | .737 | -0.13 | [-0.30, | 0.04] | .132 |
| Transfer student | 0.04 | [-0.14, | 0.21] | .681 | 0.00 | [-0.21, | 0.21] | .989 | 0.04 | [-0.14, | 0.22] | .676 | 0.05 | [-0.17, | 0.27] | .656 |
| Week 6 | -0.17 | [-0.80, | 0.47] | .604 | -0.19 | [-0.84, | 0.46] | .562 | -0.25 | [-0.65, | 0.14] | .211 | -0.74 | [-1.26, | -0.22] | .005 |
| Week 7 | -0.02 | [-0.21, | 0.17] | .807 | 0.02 | [-0.20, | 0.23] | .886 | 0.09 | [-0.15, | 0.32] | .467 | 0.10 | [-0.12, | 0.32] | .383 |
| Week 8 | 0.15 | [-0.08, | 0.37] | .212 | 0.03 | [-0.29, | 0.34] | .872 | 0.36 | [0.00, | 0.72] | .051 | 0.00 | [-0.32, | 0.31] | .979 |
| Support T3 on |  |  |  |  |  |  |  |  |  |  |  |  |  |  |  |  |
| Support T2 | 0.42 | [0.30, | 0.54] | <.001 | 0.41 | [0.29, | 0.53] | <.001 | 0.60 | [0.49, | 0.71] | <.001 | 0.61 | [0.49, | 0.72] | <.001 |
| Support T1 | 0.38 | [0.27, | 0.49] | <.001 | 0.37 | [0.26, | 0.49] | <.001 | 0.24 | [0.14, | 0.34] | <.001 | 0.25 | [0.14, | 0.35] | <.001 |
| Positive STV T2 | -0.09 | [-0.20, | 0.04] | .164 | 0.01 | [-0.11, | 0.13] | .836 | 0.00 | [-0.14, | 0.15] | .991 | -0.02 | [-0.13, | 0.10] | .792 |
| Positive STV T1 | 0.07 | [-0.05, | 0.19] | .266 | 0.06 | [-0.06, | 0.17] | .343 | 0.02 | [-0.11, | 0.16] | .739 | -0.01 | [-0.12, | 0.09] | .814 |
| GPA | -0.03 | [-0.10, | 0.05] | .482 | -0.02 | [-0.09, | 0.05] | .605 | -0.02 | [-0.09, | 0.04] | .513 | -0.02 | [-0.09, | 0.04] | .479 |
| Gender (1 = male) | -0.04 | [-0.22, | 0.13] | .639 | -0.05 | [-0.24, | 0.13] | .572 | -0.15 | [-0.30, | 0.01] | .060 | -0.14 | [-0.29, | 0.02] | .085 |
| Ethnicity: Hispanic | 0.01 | [-0.19, | 0.20] | .957 | -0.01 | [-0.20, | 0.19] | .939 | -0.14 | [-0.32, | 0.03] | .101 | -0.16 | [-0.33, | 0.02] | .075 |
| Ethnicity: White | 0.10 | [-0.12, | 0.32] | .392 | 0.06 | [-0.16, | 0.28] | .586 | 0.13 | [-0.10, | 0.35] | .264 | 0.13 | [-0.10, | 0.35] | .267 |
| Ethnicity: Other | -0.04 | [-0.35, | 0.27] | .802 | -0.05 | [-0.36, | 0.27] | .761 | -0.39 | [-0.74, | -0.04] | .029 | -0.41 | [-0.75, | -0.06] | .020 |
| FG student | 0.01 | [-0.16, | 0.17] | .932 | 0.01 | [-0.16, | 0.17] | .932 | 0.03 | [-0.12, | 0.18] | .686 | 0.03 | [-0.12, | 0.18] | .702 |
| Transfer student | 0.14 | [-0.04, | 0.32] | .122 | 0.15 | [-0.03, | 0.33] | .097 | -0.26 | [-0.47, | -0.06] | .010 | -0.27 | [-0.48, | -0.07] | .010 |
| Week 6 | 0.18 | [-0.30, | 0.66] | .467 | 0.14 | [-0.38, | 0.66] | .599 | 0.51 | [0.09, | 0.93] | .017 | 0.56 | [0.13, | 0.98] | .011 |
| Week 7 | -0.12 | [-0.37, | 0.12] | .320 | -0.12 | [-0.37, | 0.12] | .331 | -0.14 | [-0.44, | 0.16] | .359 | -0.13 | [-0.43, | 0.17] | .387 |
| Week 8 | -0.01 | [-0.25, | 0.23] | .913 | -0.05 | [-0.29, | 0.18] | .654 | 0.22 | [-0.06, | 0.50] | .129 | 0.21 | [-0.05, | 0.46] | .112 |

*Note.* GPA = grade point average; FG = first-generation; STV = subjective task value.

**Table G6**

*Coefficients from Path Models for Cost in the Fall 2020 Data*

|  | Most difficult course | | | | | | | | Most important course | | | | | | | |
| --- | --- | --- | --- | --- | --- | --- | --- | --- | --- | --- | --- | --- | --- | --- | --- | --- |
|  | Peer support | | | | Faculty support | | | | Peer support | | | | Faculty support | | | |
|  | β | 95% CI | | *p* | β | 95% CI | | *p* | β | 95% CI | | *p* | β | 95% CI | | *p* |
| Cost T2 on |  |  |  |  |  |  |  |  |  |  |  |  |  |  |  |  |
| Cost T1 | 0.37 | [0.27, | 0.47] | <.001 | 0.37 | [0.27, | 0.47] | <.001 | 0.31 | [0.21, | 0.42] | <.001 | 0.31 | [0.21, | 0.42] | <.001 |
| Support T1 | -0.01 | [-0.11, | 0.09] | .869 | 0.01 | [-0.10, | 0.12] | .896 | 0.01 | [-0.09, | 0.12] | .797 | 0.01 | [-0.10, | 0.11] | .926 |
| GPA | -0.01 | [-0.09, | 0.08] | .860 | -0.01 | [-0.09, | 0.08] | .863 | 0.04 | [-0.06, | 0.13] | .455 | 0.03 | [-0.06, | 0.13] | .462 |
| Gender (1 = male) | -0.24 | [-0.45, | -0.04] | .021 | -0.25 | [-0.45, | -0.04] | .020 | 0.11 | [-0.11, | 0.34] | .325 | 0.12 | [-0.11, | 0.34] | .314 |
| Ethnicity: Hispanic | 0.02 | [-0.21, | 0.24] | .886 | 0.02 | [-0.20, | 0.24] | .864 | -0.11 | [-0.34, | 0.11] | .331 | -0.11 | [-0.34, | 0.11] | .311 |
| Ethnicity: White | 0.07 | [-0.22, | 0.35] | .644 | 0.07 | [-0.21, | 0.36] | .624 | -0.18 | [-0.48, | 0.13] | .250 | -0.18 | [-0.49, | 0.12] | .233 |
| Ethnicity: Other | -0.27 | [-0.72, | 0.18] | .238 | -0.29 | [-0.74, | 0.16] | .211 | 0.01 | [-0.49, | 0.51] | .966 | 0.02 | [-0.48, | 0.51] | .950 |
| FG student | 0.02 | [-0.18, | 0.23] | .827 | 0.02 | [-0.18, | 0.23] | .831 | 0.00 | [-0.22, | 0.21] | .970 | 0.00 | [-0.22, | 0.21] | .986 |
| Transfer student | -0.03 | [-0.25, | 0.20] | .830 | -0.03 | [-0.25, | 0.20] | .818 | -0.05 | [-0.31, | 0.20] | .678 | -0.06 | [-0.31, | 0.20] | .668 |
| Week 6 | 0.19 | [-0.32, | 0.71] | .459 | 0.19 | [-0.33, | 0.72] | .476 | -0.58 | [-1.31, | 0.14] | .116 | -0.57 | [-1.31, | 0.16] | .128 |
| Week 7 | 0.01 | [-0.24, | 0.25] | .967 | 0.01 | [-0.24, | 0.25] | .967 | -0.04 | [-0.29, | 0.21] | .751 | -0.04 | [-0.29, | 0.21] | .753 |
| Week 8 | -0.04 | [-0.37, | 0.29] | .819 | -0.04 | [-0.36, | 0.29] | .818 | 0.60 | [0.26, | 0.93] | <.001 | 0.60 | [0.26, | 0.93] | <.001 |
| Support T2 on |  |  |  |  |  |  |  |  |  |  |  |  |  |  |  |  |
| Support T1 | 0.71 | [0.64, | 0.78] | <.001 | 0.64 | [0.56, | 0.73] | <.001 | 0.72 | [0.65, | 0.79] | <.001 | 0.66 | [0.57, | 0.74] | <.001 |
| Cost T1 | -0.01 | [-0.09, | 0.07] | .784 | -0.01 | [-0.09, | 0.08] | .861 | 0.06 | [-0.02, | 0.13] | .148 | 0.01 | [-0.07, | 0.09] | .836 |
| GPA | -0.08 | [-0.16, | -0.01] | .038 | -0.04 | [-0.12, | 0.04] | .293 | -0.08 | [-0.16, | 0.00] | .043 | -0.04 | [-0.12, | 0.04] | .355 |
| Gender (1 = male) | 0.01 | [-0.16, | 0.18] | .908 | 0.09 | [-0.09, | 0.26] | .339 | 0.02 | [-0.15, | 0.19] | .816 | 0.11 | [-0.06, | 0.29] | .207 |
| Ethnicity: Hispanic | 0.10 | [-0.09, | 0.28] | .295 | -0.02 | [-0.23, | 0.18] | .821 | 0.09 | [-0.09, | 0.27] | .341 | -0.02 | [-0.22, | 0.19] | .875 |
| Ethnicity: White | 0.13 | [-0.10, | 0.35] | .271 | 0.08 | [-0.16, | 0.32] | .525 | 0.11 | [-0.11, | 0.32] | .325 | 0.06 | [-0.18, | 0.31] | .603 |
| Ethnicity: Other | 0.25 | [-0.21, | 0.71] | .287 | -0.13 | [-0.47, | 0.20] | .437 | 0.26 | [-0.19, | 0.71] | .253 | -0.12 | [-0.44, | 0.21] | .485 |
| FG student | 0.01 | [-0.15, | 0.18] | .898 | -0.12 | [-0.29, | 0.06] | .179 | 0.02 | [-0.15, | 0.18] | .842 | -0.14 | [-0.31, | 0.04] | .125 |
| Transfer student | 0.03 | [-0.15, | 0.20] | .777 | 0.01 | [-0.20, | 0.23] | .906 | 0.03 | [-0.15, | 0.21] | .741 | 0.05 | [-0.17, | 0.27] | .675 |
| Week 6 | -0.16 | [-0.82, | 0.50] | .637 | -0.20 | [-0.87, | 0.47] | .560 | -0.25 | [-0.64, | 0.14] | .205 | -0.73 | [-1.25, | -0.20] | .007 |
| Week 7 | -0.03 | [-0.22, | 0.16] | .788 | 0.01 | [-0.20, | 0.23] | .897 | 0.10 | [-0.12, | 0.33] | .367 | 0.10 | [-0.12, | 0.32] | .380 |
| Week 8 | 0.16 | [-0.07, | 0.38] | .174 | 0.00 | [-0.32, | 0.31] | .992 | 0.34 | [-0.01, | 0.69] | .059 | -0.01 | [-0.34, | 0.31] | .939 |
| Support T3 on |  |  |  |  |  |  |  |  |  |  |  |  |  |  |  |  |
| Support T2 | 0.41 | [0.31, | 0.52] | <.001 | 0.41 | [0.30, | 0.52] | <.001 | 0.56 | [0.46, | 0.67] | <.001 | 0.56 | [0.46, | 0.66] | <.001 |
| Support T1 | 0.31 | [0.20, | 0.42] | <.001 | 0.31 | [0.20, | 0.43] | <.001 | 0.24 | [0.14, | 0.35] | <.001 | 0.24 | [0.14, | 0.35] | <.001 |
| Cost T2 | 0.07 | [-0.07, | 0.22] | .313 | -0.05 | [-0.18, | 0.07] | .404 | -0.07 | [-0.20, | 0.06] | .305 | -0.06 | [-0.18, | 0.07] | .363 |
| Cost T1 | -0.13 | [-0.27, | 0.02] | .082 | 0.01 | [-0.13, | 0.14] | .945 | 0.13 | [0.01, | 0.25] | .036 | 0.04 | [-0.09, | 0.16] | .531 |
| GPA | -0.05 | [-0.14, | 0.05] | .353 | -0.05 | [-0.15, | 0.05] | .302 | 0.03 | [-0.05, | 0.12] | .461 | 0.03 | [-0.05, | 0.11] | .441 |
| Gender (1 = male) | 0.11 | [-0.09, | 0.32] | .277 | 0.11 | [-0.09, | 0.32] | .278 | -0.10 | [-0.31, | 0.12] | .376 | -0.08 | [-0.29, | 0.13] | .470 |
| Ethnicity: Hispanic | -0.07 | [-0.30, | 0.16] | .537 | -0.04 | [-0.27, | 0.19] | .758 | -0.10 | [-0.29, | 0.09] | .304 | -0.14 | [-0.33, | 0.05] | .143 |
| Ethnicity: White | 0.06 | [-0.22, | 0.33] | .689 | 0.08 | [-0.19, | 0.36] | .545 | -0.03 | [-0.30, | 0.24] | .834 | -0.05 | [-0.32, | 0.21] | .697 |
| Ethnicity: Other | -0.20 | [-0.56, | 0.16] | .268 | -0.19 | [-0.55, | 0.18] | .323 | 0.14 | [-0.47, | 0.75] | .656 | 0.08 | [-0.52, | 0.69] | .788 |
| FG student | 0.05 | [-0.16, | 0.26] | .649 | 0.05 | [-0.16, | 0.27] | .627 | -0.11 | [-0.29, | 0.07] | .219 | -0.13 | [-0.30, | 0.05] | .169 |
| Transfer student | 0.13 | [-0.10, | 0.35] | .272 | 0.13 | [-0.09, | 0.36] | .238 | 0.03 | [-0.19, | 0.25] | .803 | 0.01 | [-0.21, | 0.23] | .921 |
| Week 6 | -0.09 | [-0.48, | 0.30] | .637 | -0.07 | [-0.42, | 0.27] | .675 | 0.68 | [-0.20, | 1.55] | .130 | 0.73 | [-0.17, | 1.64] | .111 |
| Week 7 | 0.23 | [-0.01, | 0.48] | .059 | 0.23 | [-0.01, | 0.47] | .058 | 0.01 | [-0.24, | 0.25] | .940 | 0.02 | [-0.22, | 0.27] | .855 |
| Week 8 | 0.09 | [-0.25, | 0.43] | .609 | 0.12 | [-0.23, | 0.46] | .517 | -0.14 | [-0.47, | 0.19] | .394 | -0.18 | [-0.52, | 0.16] | .297 |

*Note.* GPA = grade point average; FG = first-generation.

**Appendix H**

**Differences in Stability and Cross-lagged Coefficients Between the Fall 2019 and Fall 2020 Data**

|  | Most Difficult Course | | | | | | | | Most Important Course | | | | | | | |
| --- | --- | --- | --- | --- | --- | --- | --- | --- | --- | --- | --- | --- | --- | --- | --- | --- |
|  | Peer support | | | | Faculty support | | | | Peer support | | | | Faculty support | | | |
|  | β | 95% CI | | *p* | β | 95% CI | | *p* | β | 95% CI | | *p* | β | 95% CI | | *p* |
| Expectancy |  |  |  |  |  |  |  |  |  |  |  |  |  |  |  |  |
| Stability coefficients |  |  |  |  |  |  |  |  |  |  |  |  |  |  |  |  |
| Support T1 🡪 Support T2 | 0.00 | [-0.11, | 0.12] | .951 | 0.00 | [-0.14, | 0.13] | .961 | -0.02 | [-0.13, | 0.09] | .762 | -0.01 | [-0.14, | 0.12] | .885 |
| Expectancy T1 🡪 Expectancy T2 | 0.01 | [-0.17, | 0.19] | .898 | 0.03 | [-0.16, | 0.21] | .782 | -0.13 | [-0.27, | 0.02] | .081 | -0.12 | [-0.27, | 0.02] | .099 |
| Expectancy T1 🡪 Expectancy T3 | -0.18 | [-0.35, | -0.01] | .043 | -0.17 | [-0.35, | 0.00] | .055 | -0.26 | [-0.46, | -0.06] | .009 | -0.29 | [-0.47, | -0.10] | .003 |
| Expectancy T2 🡪 Expectancy T3 | -0.05 | [-0.22, | 0.12] | .565 | -0.03 | [-0.21, | 0.14] | .711 | 0.09 | [-0.07, | 0.25] | .265 | 0.09 | [-0.07, | 0.25] | .260 |
| Cross-lagged coefficients |  |  |  |  |  |  |  |  |  |  |  |  |  |  |  |  |
| Support T1 🡪 Expectancy T2 | -0.07 | [-0.24, | 0.10] | .399 | -0.07 | [-0.25, | 0.12] | .468 | -0.03 | [-0.19, | 0.13] | .715 | -0.02 | [-0.18, | 0.14] | .823 |
| Support T1 🡪 Expectancy T3 | 0.16 | [-0.06, | 0.38] | .165 | -0.03 | [-0.23, | 0.16] | .744 | -0.14 | [-0.36, | 0.08] | .209 | -0.07 | [-0.28, | 0.13] | .471 |
| Support T2 🡪 Expectancy T3 | -0.18 | [-0.40, | 0.04] | .100 | -0.04 | [-0.22, | 0.15] | .698 | 0.18 | [-0.04, | 0.40] | .115 | 0.20 | [0.01, | 0.39] | .040 |
| Expectancy T1 🡪 Support T2 | 0.00 | [-0.12, | 0.12] | .995 | 0.07 | [-0.07, | 0.21] | .332 | 0.04 | [-0.08, | 0.15] | .543 | 0.05 | [-0.08, | 0.18] | .432 |
| Positive STV |  |  |  |  |  |  |  |  |  |  |  |  |  |  |  |  |
| Stability coefficients |  |  |  |  |  |  |  |  |  |  |  |  |  |  |  |  |
| Support T1 🡪 Support T2 | -0.01 | [-0.12, | 0.10] | .855 | 0.01 | [-0.12, | 0.14] | .929 | -0.03 | [-0.14, | 0.08] | .635 | -0.02 | [-0.15, | 0.12] | .825 |
| Positive STV T1 🡪 Positive STV T2 | -0.04 | [-0.18, | 0.09] | .533 | -0.04 | [-0.19, | 0.10] | .548 | -0.09 | [-0.22, | 0.04] | .164 | -0.08 | [-0.21, | 0.05] | .225 |
| Positive STV T1 🡪 Positive STV T3 | -0.05 | [-0.25, | 0.15] | .625 | -0.03 | [-0.23, | 0.17] | .753 | -0.22 | [-0.38, | -0.05] | .009 | -0.22 | [-0.39, | -0.06] | .008 |
| Positive STV T2 🡪 Positive STV T3 | -0.11 | [-0.29, | 0.07] | .223 | -0.13 | [-0.32, | 0.07] | .193 | 0.16 | [0.01, | 0.31] | .032 | 0.17 | [0.02, | 0.32] | .031 |
| Cross-lagged coefficients |  |  |  |  |  |  |  |  |  |  |  |  |  |  |  |  |
| Support T1 🡪 Positive STV T2 | 0.05 | [-0.09, | 0.20] | .490 | 0.04 | [-0.12, | 0.19] | .658 | 0.04 | [-0.11, | 0.19] | .570 | 0.03 | [-0.12, | 0.17] | .727 |
| Support T1 🡪 Positive STV T3 | 0.18 | [-0.01, | 0.37] | .061 | 0.00 | [-0.18, | 0.19] | .963 | 0.10 | [-0.10, | 0.29] | .340 | -0.02 | [-0.18, | 0.14] | .844 |
| Support T2 🡪 Positive STV T3 | -0.14 | [-0.34, | 0.06] | .167 | 0.03 | [-0.15, | 0.20] | .772 | -0.09 | [-0.27, | 0.10] | .366 | 0.06 | [-0.09, | 0.22] | .409 |
| Positive STV T1 🡪 Support T2 | 0.09 | [-0.02, | 0.21] | .102 | -0.01 | [-0.13, | 0.12] | .925 | 0.10 | [-0.02, | 0.22] | .086 | 0.04 | [-0.09, | 0.17] | .543 |
| Cost |  |  |  |  |  |  |  |  |  |  |  |  |  |  |  |  |
| Stability coefficients |  |  |  |  |  |  |  |  |  |  |  |  |  |  |  |  |
| Support T1 🡪 Support T2 | 0.01 | [-0.10, | 0.12] | .890 | 0.00 | [-0.12, | 0.13] | .957 | -0.01 | [-0.12, | 0.10] | .924 | -0.01 | [-0.13, | 0.12] | .913 |
| Cost T1 🡪 Cost T2 | 0.05 | [-0.11, | 0.21] | .571 | 0.04 | [-0.12, | 0.20] | .614 | 0.22 | [0.06, | 0.38] | .006 | 0.22 | [0.06, | 0.38] | .007 |
| Cost T1 🡪 Cost T3 | 0.01 | [-0.16, | 0.19] | .898 | 0.00 | [-0.17, | 0.18] | .967 | -0.19 | [-0.36, | -0.03] | .024 | -0.17 | [-0.34, | -0.01] | .037 |
| Cost T2 🡪 Cost T3 | -0.05 | [-0.23, | 0.12] | .539 | -0.05 | [-0.23, | 0.12] | .561 | 0.06 | [-0.09, | 0.22] | .422 | 0.06 | [-0.09, | 0.22] | .430 |
| Cross-lagged coefficients |  |  |  |  |  |  |  |  |  |  |  |  |  |  |  |  |
| Support T1 🡪 Cost T2 | -0.01 | [-0.16, | 0.14] | .863 | -0.05 | [-0.20, | 0.11] | .562 | -0.01 | [-0.15, | 0.14] | .933 | 0.00 | [-0.14, | 0.15] | .971 |
| Support T1 🡪 Cost T3 | -0.11 | [-0.31, | 0.10] | .298 | 0.07 | [-0.12, | 0.26] | .460 | 0.15 | [-0.05, | 0.36] | .149 | 0.17 | [-0.03, | 0.37] | .096 |
| Support T2 🡪 Cost T3 | 0.31 | [0.10, | 0.52] | .004 | 0.09 | [-0.10, | 0.28] | .357 | -0.14 | [-0.33, | 0.05] | .159 | -0.08 | [-0.28, | 0.13] | .460 |
| Cost T1 🡪 Support T2 | 0.10 | [-0.02, | 0.22] | .092 | -0.04 | [-0.17, | 0.10] | .596 | 0.01 | [-0.10, | 0.12] | .899 | -0.08 | [-0.20, | 0.05] | .231 |

*Note.* STV = subjective task value

**Appendix I**

**Tables for Multi-Group Analyses**

**Table I1**

*Stability and Cross-Lagged Coefficients Path Models for FG and CG Students and Expectancy in the Fall 2019 Data*

|  | Most Difficult Course | | | | | | | | Most Important Course | | | | | | | |
| --- | --- | --- | --- | --- | --- | --- | --- | --- | --- | --- | --- | --- | --- | --- | --- | --- |
|  | Peer support | | | | Faculty support | | | | Peer support | | | | Faculty support | | | |
|  | β | 95% CI | | *p* | β | 95% CI | | *p* | β | 95% CI | | *p* | β | 95% CI | | *p* |
| CG student |  |  |  |  |  |  |  |  |  |  |  |  |  |  |  |  |
| Stability coefficients |  |  |  |  |  |  |  |  |  |  |  |  |  |  |  |  |
| Support T1 🡪 Support T2 | 0.72 | [0.60, | 0.85] | <.001 | 0.54 | [0.38, | 0.70] | <.001 | 0.72 | [0.60, | 0.84] | <.001 | 0.56 | [0.40, | 0.71] | <.001 |
| Expectancy T1 🡪 Expectancy T2 | 0.13 | [-0.08, | 0.35] | .227 | 0.12 | [-0.11, | 0.35] | .291 | 0.11 | [-0.06, | 0.28] | .199 | 0.09 | [-0.08, | 0.26] | .297 |
| Expectancy T2 🡪 Expectancy T3 | 0.16 | [-0.04, | 0.36] | .117 | 0.17 | [-0.04, | 0.38] | .106 | 0.36 | [0.17, | 0.55] | <.001 | 0.36 | [0.19, | 0.53] | <.001 |
| Expectancy T1 🡪 Expectancy T3 | 0.40 | [0.22, | 0.57] | <.001 | 0.41 | [0.23, | 0.59] | <.001 | 0.39 | [0.26, | 0.52] | <.001 | 0.39 | [0.26, | 0.52] | <.001 |
| Cross-lagged coefficients |  |  |  |  |  |  |  |  |  |  |  |  |  |  |  |  |
| Support T1 🡪 Expectancy T2 | 0.02 | [-0.18, | 0.22] | .877 | 0.05 | [-0.15, | 0.25] | .643 | -0.14 | [-0.31, | 0.04] | .136 | 0.08 | [-0.11, | 0.28] | .401 |
| Support T2 🡪 Expectancy T3 | -0.16 | [-0.46, | 0.13] | .281 | 0.00 | [-0.22, | 0.23] | .985 | -0.14 | [-0.38, | 0.09] | .235 | -0.13 | [-0.31, | 0.06] | .172 |
| Support T1 🡪 Expectancy T3 | 0.05 | [-0.19, | 0.29] | .696 | -0.06 | [-0.26, | 0.14] | .540 | 0.14 | [-0.09, | 0.37] | .246 | 0.10 | [-0.07, | 0.26] | .258 |
| Expectancy T1 🡪 Support T2 | 0.02 | [-0.13, | 0.16] | .830 | 0.10 | [-0.09, | 0.29] | .289 | 0.01 | [-0.13, | 0.14] | .937 | 0.05 | [-0.11, | 0.22] | .529 |
| FG students |  |  |  |  |  |  |  |  |  |  |  |  |  |  |  |  |
| Stability coefficients |  |  |  |  |  |  |  |  |  |  |  |  |  |  |  |  |
| Support T1 🡪 Support T2 | 0.72 | [0.60, | 0.84] | <.001 | 0.68 | [0.56, | 0.81] | <.001 | 0.72 | [0.60, | 0.83] | <.001 | 0.69 | [0.57, | 0.80] | <.001 |
| Expectancy T1 🡪 Expectancy T2 | 0.43 | [0.25, | 0.61] | <.001 | 0.40 | [0.22, | 0.58] | <.001 | 0.42 | [0.29, | 0.56] | <.001 | 0.41 | [0.28, | 0.54] | <.001 |
| Expectancy T2 🡪 Expectancy T3 | 0.36 | [0.20, | 0.53] | <.001 | 0.39 | [0.21, | 0.57] | <.001 | 0.19 | [-0.04, | 0.42] | .108 | 0.18 | [-0.04, | 0.39] | .108 |
| Expectancy T1 🡪 Expectancy T3 | 0.16 | [-0.03, | 0.35] | .089 | 0.14 | [-0.06, | 0.33] | .162 | 0.20 | [0.04, | 0.35] | .011 | 0.18 | [0.03, | 0.32] | .015 |
| Cross-lagged coefficients |  |  |  |  |  |  |  |  |  |  |  |  |  |  |  |  |
| Support T1 🡪 Expectancy T2 | -0.02 | [-0.17, | 0.12] | .771 | 0.10 | [-0.06, | 0.26] | .217 | 0.06 | [-0.09, | 0.20] | .436 | 0.12 | [-0.05, | 0.28] | .162 |
| Support T2 🡪 Expectancy T3 | 0.30 | [0.11, | 0.49] | .002 | 0.06 | [-0.15, | 0.28] | .560 | 0.12 | [-0.10, | 0.35] | .287 | -0.04 | [-0.26, | 0.18] | .720 |
| Support T1 🡪 Expectancy T3 | -0.20 | [-0.41, | 0.02] | .074 | 0.02 | [-0.16, | 0.20] | .792 | 0.03 | [-0.22, | 0.27] | .815 | 0.26 | [0.06, | 0.47] | .012 |
| Expectancy T1 🡪 Support T2 | 0.03 | [-0.11, | 0.16] | .694 | 0.15 | [0.05, | 0.25] | .002 | 0.03 | [-0.08, | 0.14] | .599 | 0.13 | [0.02, | 0.23] | .018 |
| Difference |  |  |  |  |  |  |  |  |  |  |  |  |  |  |  |  |
| Stability coefficients |  |  |  |  |  |  |  |  |  |  |  |  |  |  |  |  |
| Support T1 🡪 Support T2 | 0.01 | [-0.17, | 0.18] | .953 | -0.14 | [-0.34, | 0.06] | .162 | 0.00 | [-0.17, | 0.17] | .985 | -0.13 | [-0.32, | 0.07] | .192 |
| Expectancy T1 🡪 Expectancy T2 | -0.30 | [-0.58, | -0.02] | .037 | -0.28 | [-0.57, | 0.01] | .062 | -0.31 | [-0.53, | -0.10] | .004 | -0.32 | [-0.53, | -0.10] | .004 |
| Expectancy T2 🡪 Expectancy T3 | -0.20 | [-0.47, | 0.06] | .129 | -0.22 | [-0.50, | 0.06] | .117 | 0.17 | [-0.12, | 0.47] | .246 | 0.18 | [-0.09, | 0.45] | .195 |
| Expectancy T1 🡪 Expectancy T3 | 0.23 | [-0.02, | 0.49] | .075 | 0.27 | [0.01, | 0.53] | .046 | 0.20 | [-0.01, | 0.40] | .057 | 0.21 | [0.01, | 0.40] | .036 |
| Cross-lagged coefficients |  |  |  |  |  |  |  |  |  |  |  |  |  |  |  |  |
| Support T1 🡪 Expectancy T2 | 0.04 | [-0.21, | 0.28] | .768 | -0.05 | [-0.31, | 0.21] | .691 | -0.19 | [-0.42, | 0.04] | .099 | -0.04 | [-0.29, | 0.22] | .789 |
| Support T2 🡪 Expectancy T3 | -0.46 | [-0.82, | -0.11] | .010 | -0.06 | [-0.37, | 0.25] | .695 | -0.27 | [-0.59, | 0.06] | .111 | -0.09 | [-0.37, | 0.20] | .539 |
| Support T1 🡪 Expectancy T3 | 0.24 | [-0.08, | 0.57] | .137 | -0.09 | [-0.35, | 0.18] | .529 | 0.11 | [-0.23, | 0.44] | .533 | -0.17 | [-0.43, | 0.10] | .216 |
| Expectancy T1 🡪 Support T2 | -0.01 | [-0.21, | 0.19] | .917 | -0.05 | [-0.26, | 0.17] | .674 | -0.02 | [-0.20, | 0.15] | .781 | -0.07 | [-0.27, | 0.12] | .462 |

**Table I2**

*Stability and Cross-Lagged Coefficients Path Models for FG and CG Students and Positive STV in the Fall 2019 Data*

|  | Most Difficult Course | | | | | | | | Most Important Course | | | | | | | |
| --- | --- | --- | --- | --- | --- | --- | --- | --- | --- | --- | --- | --- | --- | --- | --- | --- |
|  | Peer support | | | | Faculty support | | | | Peer support | | | | Faculty support | | | |
|  | β | 95% CI | | *p* | β | 95% CI | | *p* | β | 95% CI | | *p* | β | 95% CI | | *p* |
| CG student |  |  |  |  |  |  |  |  |  |  |  |  |  |  |  |  |
| Stability coefficients |  |  |  |  |  |  |  |  |  |  |  |  |  |  |  |  |
| Support T1 🡪 Support T2 | 0.71 | [0.59, | 0.84] | <.001 | 0.56 | [0.39, | 0.72] | <.001 | 0.71 | [0.58, | 0.83] | <.001 | 0.54 | [0.37, | 0.71] | <.001 |
| Positive STV T1 🡪 Positive STV T2 | 0.54 | [0.40, | 0.69] | <.001 | 0.54 | [0.39, | 0.69] | <.001 | 0.57 | [0.43, | 0.71] | <.001 | 0.55 | [0.41, | 0.70] | <.001 |
| Positive STV T2 🡪 Positive STV T3 | 0.50 | [0.29, | 0.71] | <.001 | 0.49 | [0.27, | 0.70] | <.001 | 0.38 | [0.19, | 0.57] | <.001 | 0.38 | [0.17, | 0.58] | <.001 |
| Positive STV T1 🡪 Positive STV T3 | 0.20 | [0.01, | 0.40] | .044 | 0.21 | [-0.01, | 0.43] | .065 | 0.43 | [0.26, | 0.61] | <.001 | 0.42 | [0.25, | 0.60] | <.001 |
| Cross-lagged coefficients |  |  |  |  |  |  |  |  |  |  |  |  |  |  |  |  |
| Support T1 🡪 Positive STV T2 | -0.04 | [-0.19, | 0.11] | .577 | 0.00 | [-0.19, | 0.19] | .978 | -0.04 | [-0.19, | 0.10] | .571 | 0.04 | [-0.11, | 0.18] | .648 |
| Support T2 🡪 Positive STV T3 | 0.14 | [-0.10, | 0.39] | .239 | -0.09 | [-0.30, | 0.12] | .415 | 0.00 | [-0.20, | 0.20] | .977 | -0.04 | [-0.21, | 0.12] | .604 |
| Support T1 🡪 Positive STV T3 | -0.05 | [-0.27, | 0.16] | .635 | 0.10 | [-0.12, | 0.32] | .358 | -0.04 | [-0.23, | 0.14] | .667 | 0.07 | [-0.10, | 0.24] | .425 |
| Positive STV T1 🡪 Support T2 | 0.02 | [-0.11, | 0.15] | .758 | 0.02 | [-0.15, | 0.19] | .840 | 0.07 | [-0.06, | 0.19] | .315 | 0.09 | [-0.09, | 0.26] | .335 |
| FG students |  |  |  |  |  |  |  |  |  |  |  |  |  |  |  |  |
| Stability coefficients |  |  |  |  |  |  |  |  |  |  |  |  |  |  |  |  |
| Support T1 🡪 Support T2 | 0.71 | [0.59, | 0.83] | <.001 | 0.69 | [0.57, | 0.82] | <.001 | 0.70 | [0.58, | 0.81] | <.001 | 0.70 | [0.58, | 0.82] | <.001 |
| Positive STV T1 🡪 Positive STV T2 | 0.59 | [0.44, | 0.75] | <.001 | 0.59 | [0.43, | 0.75] | <.001 | 0.49 | [0.35, | 0.63] | <.001 | 0.49 | [0.35, | 0.64] | <.001 |
| Positive STV T2 🡪 Positive STV T3 | 0.29 | [0.07, | 0.50] | .009 | 0.31 | [0.09, | 0.52] | .006 | 0.36 | [0.22, | 0.50] | <.001 | 0.38 | [0.23, | 0.52] | <.001 |
| Positive STV T1 🡪 Positive STV T3 | 0.33 | [0.13, | 0.53] | .001 | 0.29 | [0.06, | 0.51] | .012 | 0.43 | [0.30, | 0.57] | <.001 | 0.44 | [0.30, | 0.59] | <.001 |
| Cross-lagged coefficients |  |  |  |  |  |  |  |  |  |  |  |  |  |  |  |  |
| Support T1 🡪 Positive STV T2 | 0.10 | [-0.06, | 0.26] | .235 | 0.12 | [-0.05, | 0.28] | .156 | 0.19 | [0.02, | 0.36] | .032 | 0.13 | [-0.03, | 0.29] | .119 |
| Support T2 🡪 Positive STV T3 | 0.13 | [-0.07, | 0.32] | .207 | 0.11 | [-0.08, | 0.30] | .262 | 0.13 | [-0.04, | 0.30] | .142 | 0.00 | [-0.16, | 0.17] | .970 |
| Support T1 🡪 Positive STV T3 | -0.11 | [-0.34, | 0.11] | .317 | 0.04 | [-0.13, | 0.22] | .630 | -0.06 | [-0.23, | 0.11] | .458 | 0.01 | [-0.14, | 0.16] | .940 |
| Positive STV T1 🡪 Support T2 | 0.08 | [-0.05, | 0.19] | .220 | 0.06 | [-0.06, | 0.17] | .315 | 0.15 | [0.03, | 0.27] | .014 | 0.02 | [-0.10, | 0.14] | .738 |
| Difference |  |  |  |  |  |  |  |  |  |  |  |  |  |  |  |  |
| Stability coefficients |  |  |  |  |  |  |  |  |  |  |  |  |  |  |  |  |
| Support T1 🡪 Support T2 | 0.00 | [-0.17, | 0.18] | .980 | -0.14 | [-0.34, | 0.07] | .184 | 0.01 | [-0.16, | 0.18] | .912 | -0.16 | [-0.37, | 0.05] | .126 |
| Positive STV T1 🡪 Positive STV T2 | -0.05 | [-0.26, | 0.16] | .634 | -0.05 | [-0.27, | 0.17] | .685 | 0.08 | [-0.12, | 0.28] | .430 | 0.06 | [-0.15, | 0.27] | .573 |
| Positive STV T2 🡪 Positive STV T3 | 0.21 | [-0.09, | 0.51] | .169 | 0.18 | [-0.12, | 0.49] | .239 | 0.02 | [-0.22, | 0.26] | .869 | 0.00 | [-0.25, | 0.25] | .998 |
| Positive STV T1 🡪 Positive STV T3 | -0.13 | [-0.40, | 0.15] | .379 | -0.08 | [-0.39, | 0.23] | .624 | 0.00 | [-0.22, | 0.23] | .980 | -0.02 | [-0.25, | 0.21] | .853 |
| Cross-lagged coefficients |  |  |  |  |  |  |  |  |  |  |  |  |  |  |  |  |
| Support T1 🡪 Positive STV T2 | -0.14 | [-0.36, | 0.08] | .210 | -0.12 | [-0.36, | 0.13] | .364 | -0.23 | [-0.46, | -0.01] | .045 | -0.09 | [-0.31, | 0.13] | .403 |
| Support T2 🡪 Positive STV T3 | 0.02 | [-0.29, | 0.33] | .903 | -0.20 | [-0.48, | 0.09] | .175 | -0.13 | [-0.39, | 0.14] | .345 | -0.05 | [-0.28, | 0.19] | .693 |
| Support T1 🡪 Positive STV T3 | 0.06 | [-0.25, | 0.37] | .695 | 0.06 | [-0.22, | 0.34] | .670 | 0.02 | [-0.23, | 0.27] | .854 | 0.06 | [-0.16, | 0.29] | .584 |
| Positive STV T1 🡪 Support T2 | -0.05 | [-0.23, | 0.12] | .552 | -0.04 | [-0.25, | 0.16] | .697 | -0.09 | [-0.26, | 0.09] | .341 | 0.07 | [-0.15, | 0.28] | .544 |

**Table I3**

*Stability and Cross-Lagged Coefficients Path Models for FG and CG Students and Cost in the Fall 2019 Data*

|  | Most Difficult Course | | | | | | | | Most Important Course | | | | | | | |
| --- | --- | --- | --- | --- | --- | --- | --- | --- | --- | --- | --- | --- | --- | --- | --- | --- |
|  | Peer support | | | | Faculty support | | | | Peer support | | | | Faculty support | | | |
|  | β | 95% CI | | *p* | β | 95% CI | | *p* | β | 95% CI | | *p* | β | 95% CI | | *p* |
| CG student |  |  |  |  |  |  |  |  |  |  |  |  |  |  |  |  |
| Stability coefficients |  |  |  |  |  |  |  |  |  |  |  |  |  |  |  |  |
| Support T1 🡪 Support T2 | 0.73 | [0.60, | 0.86] | <.001 | 0.54 | [0.38, | 0.70] | <.001 | 0.73 | [0.61, | 0.86] | <.001 | 0.55 | [0.39, | 0.70] | <.001 |
| Cost T1 🡪 Cost T2 | 0.46 | [0.27, | 0.65] | <.001 | 0.47 | [0.28, | 0.67] | <.001 | 0.53 | [0.35, | 0.70] | <.001 | 0.52 | [0.34, | 0.69] | <.001 |
| Cost T2 🡪 Cost T3 | 0.47 | [0.28, | 0.67] | <.001 | 0.45 | [0.25, | 0.66] | <.001 | 0.47 | [0.28, | 0.66] | <.001 | 0.49 | [0.31, | 0.68] | <.001 |
| Cost T1 🡪 Cost T3 | 0.23 | [0.03, | 0.43] | .027 | 0.22 | [0.01, | 0.43] | .043 | 0.18 | [-0.03, | 0.38] | .092 | 0.15 | [-0.06, | 0.37] | .156 |
| Cross-lagged coefficients |  |  |  |  |  |  |  |  |  |  |  |  |  |  |  |  |
| Support T1 🡪 Cost T2 | -0.10 | [-0.28, | 0.08] | .275 | -0.04 | [-0.22, | 0.14] | .635 | -0.01 | [-0.17, | 0.15] | .884 | -0.11 | [-0.28, | 0.05] | .182 |
| Support T2 🡪 Cost T3 | -0.10 | [-0.35, | 0.16] | .466 | -0.12 | [-0.32, | 0.08] | .250 | -0.03 | [-0.31, | 0.26] | .858 | -0.07 | [-0.30, | 0.15] | .516 |
| Support T1 🡪 Cost T3 | 0.23 | [-0.02, | 0.48] | .073 | 0.14 | [-0.05, | 0.32] | .155 | 0.03 | [-0.26, | 0.32] | .830 | 0.08 | [-0.12, | 0.27] | .455 |
| Cost T1 🡪 Support T2 | 0.08 | [-0.06, | 0.21] | .265 | -0.11 | [-0.28, | 0.07] | .238 | 0.06 | [-0.07, | 0.19] | .398 | -0.18 | [-0.32, | -0.03] | .016 |
| FG students |  |  |  |  |  |  |  |  |  |  |  |  |  |  |  |  |
| Stability coefficients |  |  |  |  |  |  |  |  |  |  |  |  |  |  |  |  |
| Support T1 🡪 Support T2 | 0.72 | [0.61, | 0.83] | <.001 | 0.70 | [0.58, | 0.82] | <.001 | 0.71 | [0.59, | 0.83] | <.001 | 0.71 | [0.59, | 0.83] | <.001 |
| Cost T1 🡪 Cost T2 | 0.41 | [0.24, | 0.57] | <.001 | 0.41 | [0.24, | 0.57] | <.001 | 0.55 | [0.40, | 0.70] | <.001 | 0.54 | [0.39, | 0.70] | <.001 |
| Cost T2 🡪 Cost T3 | 0.42 | [0.24, | 0.61] | <.001 | 0.43 | [0.24, | 0.61] | <.001 | 0.32 | [0.15, | 0.50] | <.001 | 0.36 | [0.20, | 0.52] | <.001 |
| Cost T1 🡪 Cost T3 | 0.30 | [0.12, | 0.48] | .001 | 0.29 | [0.10, | 0.48] | .002 | 0.38 | [0.24, | 0.52] | <.001 | 0.37 | [0.24, | 0.50] | <.001 |
| Cross-lagged coefficients |  |  |  |  |  |  |  |  |  |  |  |  |  |  |  |  |
| Support T1 🡪 Cost T2 | 0.04 | [-0.11, | 0.18] | .619 | -0.02 | [-0.18, | 0.14] | .784 | 0.01 | [-0.13, | 0.14] | .946 | 0.09 | [-0.06, | 0.23] | .255 |
| Support T2 🡪 Cost T3 | 0.00 | [-0.19, | 0.18] | .983 | 0.13 | [-0.08, | 0.33] | .229 | 0.11 | [-0.07, | 0.30] | .234 | 0.25 | [0.03, | 0.47] | .027 |
| Support T1 🡪 Cost T3 | 0.18 | [-0.03, | 0.38] | .092 | 0.03 | [-0.15, | 0.20] | .776 | -0.04 | [-0.21, | 0.13] | .639 | -0.17 | [-0.41, | 0.08] | .192 |
| Cost T1 🡪 Support T2 | 0.10 | [-0.02, | 0.21] | .088 | -0.01 | [-0.13, | 0.11] | .862 | 0.08 | [-0.04, | 0.19] | .191 | -0.01 | [-0.13, | 0.11] | .895 |
| Difference |  |  |  |  |  |  |  |  |  |  |  |  |  |  |  |  |
| Stability coefficients |  |  |  |  |  |  |  |  |  |  |  |  |  |  |  |  |
| Support T1 🡪 Support T2 | 0.01 | [-0.16, | 0.18] | .943 | -0.16 | [-0.36, | 0.04] | .115 | 0.03 | [-0.15, | 0.20] | .779 | -0.16 | [-0.35, | 0.04] | .113 |
| Cost T1 🡪 Cost T2 | 0.06 | [-0.19, | 0.31] | .652 | 0.06 | [-0.19, | 0.32] | .620 | -0.02 | [-0.26, | 0.21] | .837 | -0.03 | [-0.27, | 0.21] | .817 |
| Cost T2 🡪 Cost T3 | 0.05 | [-0.22, | 0.32] | .723 | 0.03 | [-0.25, | 0.31] | .849 | 0.15 | [-0.11, | 0.41] | .255 | 0.13 | [-0.11, | 0.37] | .299 |
| Cost T1 🡪 Cost T3 | -0.07 | [-0.34, | 0.20] | .615 | -0.08 | [-0.36, | 0.21] | .604 | -0.20 | [-0.45, | 0.05] | .116 | -0.22 | [-0.47, | 0.03] | .089 |
| Cross-lagged coefficients |  |  |  |  |  |  |  |  |  |  |  |  |  |  |  |  |
| Support T1 🡪 Cost T2 | -0.14 | [-0.36, | 0.09] | .246 | -0.02 | [-0.26, | 0.22] | .860 | -0.02 | [-0.22, | 0.19] | .876 | -0.20 | [-0.42, | 0.02] | .080 |
| Support T2 🡪 Cost T3 | -0.09 | [-0.41, | 0.22] | .564 | -0.25 | [-0.54, | 0.04] | .096 | -0.14 | [-0.48, | 0.20] | .421 | -0.32 | [-0.64, | -0.01] | .044 |
| Support T1 🡪 Cost T3 | 0.05 | [-0.27, | 0.38] | .744 | 0.11 | [-0.15, | 0.37] | .400 | 0.07 | [-0.26, | 0.41] | .673 | 0.24 | [-0.08, | 0.56] | .137 |
| Cost T1 🡪 Support T2 | -0.03 | [-0.20, | 0.15] | .783 | -0.10 | [-0.31, | 0.12] | .384 | -0.02 | [-0.19, | 0.15] | .814 | -0.17 | [-0.35, | 0.02] | .077 |

**Table I4**

*Stability and Cross-Lagged Coefficients Path Models for Transfer and Non-Transfer Students and Expectancy in the Fall 2019 Data*

|  | Most Difficult Course | | | | | | | | Most Important Course | | | | | | | |
| --- | --- | --- | --- | --- | --- | --- | --- | --- | --- | --- | --- | --- | --- | --- | --- | --- |
|  | Peer support | | | | Faculty support | | | | Peer support | | | | Faculty support | | | |
|  | β | 95% CI | | *p* | β | 95% CI | | *p* | β | 95% CI | | *p* | β | 95% CI | | *p* |
| Non-transfer student |  |  |  |  |  |  |  |  |  |  |  |  |  |  |  |  |
| Stability coefficients |  |  |  |  |  |  |  |  |  |  |  |  |  |  |  |  |
| Support T1 🡪 Support T2 | 0.72 | [0.62, | 0.81] | <.001 | 0.63 | [0.52, | 0.74] | <.001 | 0.71 | [0.62, | 0.81] | <.001 | 0.63 | [0.52, | 0.73] | <.001 |
| Expectancy T1 🡪 Expectancy T2 | 0.29 | [0.15, | 0.44] | <.001 | 0.27 | [0.12, | 0.42] | <.001 | 0.33 | [0.21, | 0.44] | <.001 | 0.30 | [0.18, | 0.42] | <.001 |
| Expectancy T2 🡪 Expectancy T3 | 0.27 | [0.12, | 0.43] | <.001 | 0.25 | [0.09, | 0.42] | .002 | 0.29 | [0.11, | 0.47] | .002 | 0.26 | [0.09, | 0.43] | .002 |
| Expectancy T1 🡪 Expectancy T3 | 0.26 | [0.11, | 0.41] | .001 | 0.25 | [0.09, | 0.40] | .002 | 0.20 | [0.08, | 0.33] | .002 | 0.18 | [0.06, | 0.31] | .004 |
| Cross-lagged coefficients |  |  |  |  |  |  |  |  |  |  |  |  |  |  |  |  |
| Support T1 🡪 Expectancy T2 | -0.07 | [-0.19, | 0.06] | .294 | 0.09 | [-0.06, | 0.23] | .250 | -0.05 | [-0.17, | 0.08] | .495 | 0.08 | [-0.06, | 0.22] | .265 |
| Support T2 🡪 Expectancy T3 | 0.08 | [-0.12, | 0.28] | .427 | 0.07 | [-0.12, | 0.26] | .466 | -0.01 | [-0.20, | 0.18] | .944 | -0.05 | [-0.22, | 0.13] | .583 |
| Support T1 🡪 Expectancy T3 | -0.04 | [-0.24, | 0.16] | .683 | 0.02 | [-0.13, | 0.18] | .781 | 0.11 | [-0.08, | 0.31] | .262 | 0.22 | [0.06, | 0.38] | .007 |
| Expectancy T1 🡪 Support T2 | 0.01 | [-0.09, | 0.12] | .842 | 0.07 | [-0.03, | 0.18] | .163 | 0.02 | [-0.08, | 0.11] | .755 | 0.09 | [-0.01, | 0.20] | .080 |
| Transfer students |  |  |  |  |  |  |  |  |  |  |  |  |  |  |  |  |
| Stability coefficients |  |  |  |  |  |  |  |  |  |  |  |  |  |  |  |  |
| Support T1 🡪 Support T2 | 0.74 | [0.54, | 0.94] | <.001 | 0.65 | [0.42, | 0.87] | <.001 | 0.73 | [0.53, | 0.94] | <.001 | 0.70 | [0.47, | 0.93] | <.001 |
| Expectancy T1 🡪 Expectancy T2 | 0.22 | [-0.09, | 0.54] | .167 | 0.23 | [-0.13, | 0.59] | .207 | 0.16 | [-0.13, | 0.44] | .282 | 0.21 | [-0.08, | 0.50] | .155 |
| Expectancy T2 🡪 Expectancy T3 | 0.29 | [0.03, | 0.56] | .031 | 0.27 | [0.02, | 0.53] | .038 | 0.34 | [0.07, | 0.60] | .013 | 0.35 | [0.09, | 0.62] | .010 |
| Expectancy T1 🡪 Expectancy T3 | 0.37 | [0.10, | 0.65] | .008 | 0.45 | [0.17, | 0.73] | .002 | 0.55 | [0.32, | 0.78] | <.001 | 0.57 | [0.37, | 0.77] | <.001 |
| Cross-lagged coefficients |  |  |  |  |  |  |  |  |  |  |  |  |  |  |  |  |
| Support T1 🡪 Expectancy T2 | 0.25 | [-0.04, | 0.53] | .086 | 0.14 | [-0.16, | 0.43] | .363 | 0.05 | [-0.21, | 0.31] | .708 | 0.35 | [0.07, | 0.62] | .013 |
| Support T2 🡪 Expectancy T3 | 0.15 | [-0.20, | 0.50] | .396 | -0.21 | [-0.51, | 0.09] | .166 | -0.11 | [-0.42, | 0.21] | .502 | -0.34 | [-0.62, | -0.06] | .017 |
| Support T1 🡪 Expectancy T3 | -0.28 | [-0.58, | 0.03] | .075 | 0.05 | [-0.24, | 0.34] | .739 | 0.03 | [-0.27, | 0.32] | .852 | 0.18 | [-0.13, | 0.48] | .253 |
| Expectancy T1 🡪 Support T2 | 0.03 | [-0.15, | 0.21] | .749 | 0.18 | [-0.04, | 0.41] | .107 | 0.08 | [-0.11, | 0.27] | .405 | 0.10 | [-0.12, | 0.32] | .360 |
| Difference |  |  |  |  |  |  |  |  |  |  |  |  |  |  |  |  |
| Stability coefficients |  |  |  |  |  |  |  |  |  |  |  |  |  |  |  |  |
| Support T1 🡪 Support T2 | -0.03 | [-0.25, | 0.20] | .822 | -0.02 | [-0.27, | 0.24] | .898 | -0.02 | [-0.25, | 0.21] | .862 | -0.07 | [-0.32, | 0.18] | .578 |
| Expectancy T1 🡪 Expectancy T2 | 0.07 | [-0.28, | 0.42] | .700 | 0.04 | [-0.35, | 0.43] | .855 | 0.17 | [-0.14, | 0.48] | .273 | 0.09 | [-0.22, | 0.41] | .554 |
| Expectancy T2 🡪 Expectancy T3 | -0.02 | [-0.32, | 0.29] | .913 | -0.02 | [-0.32, | 0.28] | .901 | -0.05 | [-0.37, | 0.27] | .764 | -0.09 | [-0.40, | 0.23] | .577 |
| Expectancy T1 🡪 Expectancy T3 | -0.11 | [-0.43, | 0.20] | .480 | -0.20 | [-0.52, | 0.12] | .220 | -0.34 | [-0.61, | -0.08] | .011 | -0.38 | [-0.62, | -0.15] | .002 |
| Cross-lagged coefficients |  |  |  |  |  |  |  |  |  |  |  |  |  |  |  |  |
| Support T1 🡪 Expectancy T2 | -0.31 | [-0.62, | -0.01] | .046 | -0.05 | [-0.38, | 0.28] | .769 | -0.09 | [-0.38, | 0.20] | .524 | -0.26 | [-0.57, | 0.04] | .090 |
| Support T2 🡪 Expectancy T3 | -0.07 | [-0.47, | 0.33] | .738 | 0.28 | [-0.07, | 0.63] | .119 | 0.10 | [-0.27, | 0.47] | .591 | 0.29 | [-0.04, | 0.62] | .084 |
| Support T1 🡪 Expectancy T3 | 0.24 | [-0.13, | 0.60] | .205 | -0.03 | [-0.36, | 0.30] | .871 | 0.09 | [-0.27, | 0.44] | .640 | 0.04 | [-0.30, | 0.38] | .820 |
| Expectancy T1 🡪 Support T2 | -0.02 | [-0.23, | 0.19] | .859 | -0.11 | [-0.36, | 0.14] | .380 | -0.07 | [-0.28, | 0.15] | .551 | -0.01 | [-0.25, | 0.24] | .944 |

**Table I5**

*Stability and Cross-Lagged Coefficients Path Models for Transfer and Non-Transfer Students and Positive STV in the Fall 2019 Data*

|  | Most Difficult Course | | | | | | | | Most Important Course | | | | | | | |
| --- | --- | --- | --- | --- | --- | --- | --- | --- | --- | --- | --- | --- | --- | --- | --- | --- |
|  | Peer support | | | | Faculty support | | | | Peer support | | | | Faculty support | | | |
|  | β | 95% CI | | *p* | β | 95% CI | | *p* | β | 95% CI | | *p* | β | 95% CI | | *p* |
| Non-transfer student |  |  |  |  |  |  |  |  |  |  |  |  |  |  |  |  |
| Stability coefficients |  |  |  |  |  |  |  |  |  |  |  |  |  |  |  |  |
| Support T1 🡪 Support T2 | 0.71 | [0.62, | 0.81] | <.001 | 0.64 | [0.53, | 0.75] | <.001 | 0.69 | [0.60, | 0.79] | <.001 | 0.64 | [0.53, | 0.74] | <.001 |
| Positive STV T1 🡪 Positive STV T2 | 0.59 | [0.48, | 0.71] | <.001 | 0.60 | [0.48, | 0.73] | <.001 | 0.58 | [0.47, | 0.70] | <.001 | 0.58 | [0.47, | 0.70] | <.001 |
| Positive STV T2 🡪 Positive STV T3 | 0.41 | [0.24, | 0.57] | <.001 | 0.42 | [0.26, | 0.58] | <.001 | 0.38 | [0.25, | 0.52] | <.001 | 0.38 | [0.24, | 0.52] | <.001 |
| Positive STV T1 🡪 Positive STV T3 | 0.25 | [0.10, | 0.40] | .001 | 0.22 | [0.06, | 0.39] | .009 | 0.37 | [0.24, | 0.51] | <.001 | 0.38 | [0.24, | 0.51] | <.001 |
| Cross-lagged coefficients |  |  |  |  |  |  |  |  |  |  |  |  |  |  |  |  |
| Support T1 🡪 Positive STV T2 | 0.00 | [-0.12, | 0.11] | .963 | -0.02 | [-0.15, | 0.12] | .803 | 0.05 | [-0.08, | 0.18] | .475 | 0.03 | [-0.10, | 0.15] | .674 |
| Support T2 🡪 Positive STV T3 | 0.00 | [-0.16, | 0.17] | .961 | 0.07 | [-0.07, | 0.21] | .304 | 0.08 | [-0.08, | 0.23] | .346 | -0.03 | [-0.16, | 0.11] | .705 |
| Support T1 🡪 Positive STV T3 | 0.03 | [-0.15, | 0.20] | .745 | 0.04 | [-0.10, | 0.18] | .589 | -0.01 | [-0.15, | 0.13] | .878 | 0.07 | [-0.05, | 0.20] | .261 |
| Positive STV T1 🡪 Support T2 | 0.05 | [-0.04, | 0.14] | .305 | 0.01 | [-0.09, | 0.12] | .799 | 0.13 | [0.03, | 0.23] | .009 | 0.04 | [-0.06, | 0.15] | .437 |
| Transfer students |  |  |  |  |  |  |  |  |  |  |  |  |  |  |  |  |
| Stability coefficients |  |  |  |  |  |  |  |  |  |  |  |  |  |  |  |  |
| Support T1 🡪 Support T2 | 0.71 | [0.51, | 0.92] | <.001 | 0.64 | [0.40, | 0.88] | <.001 | 0.74 | [0.54, | 0.95] | <.001 | 0.68 | [0.45, | 0.91] | <.001 |
| Positive STV T1 🡪 Positive STV T2 | 0.51 | [0.29, | 0.73] | <.001 | 0.45 | [0.25, | 0.66] | <.001 | 0.38 | [0.15, | 0.60] | .001 | 0.35 | [0.15, | 0.56] | .001 |
| Positive STV T2 🡪 Positive STV T3 | 0.41 | [0.11, | 0.71] | .007 | 0.51 | [0.16, | 0.87] | .005 | 0.47 | [0.27, | 0.66] | <.001 | 0.51 | [0.28, | 0.73] | <.001 |
| Positive STV T1 🡪 Positive STV T3 | 0.20 | [-0.12, | 0.52] | .220 | 0.23 | [-0.12, | 0.58] | .204 | 0.51 | [0.33, | 0.70] | <.001 | 0.54 | [0.36, | 0.72] | <.001 |
| Cross-lagged coefficients |  |  |  |  |  |  |  |  |  |  |  |  |  |  |  |  |
| Support T1 🡪 Positive STV T2 | 0.25 | [-0.04, | 0.54] | .087 | 0.45 | [0.20, | 0.71] | <.001 | 0.19 | [-0.05, | 0.43] | .120 | 0.38 | [0.18, | 0.58] | <.001 |
| Support T2 🡪 Positive STV T3 | 0.43 | [0.13, | 0.74] | .005 | -0.37 | [-0.73, | -0.01] | .047 | 0.14 | [-0.17, | 0.45] | .365 | -0.11 | [-0.30, | 0.07] | .234 |
| Support T1 🡪 Positive STV T3 | -0.41 | [-0.74, | -0.08] | .015 | 0.26 | [-0.08, | 0.60] | .132 | -0.21 | [-0.49, | 0.08] | .154 | -0.03 | [-0.26, | 0.20] | .791 |
| Positive STV T1 🡪 Support T2 | 0.08 | [-0.13, | 0.29] | .470 | 0.10 | [-0.15, | 0.35] | .432 | 0.04 | [-0.14, | 0.22] | .676 | 0.06 | [-0.15, | 0.28] | .574 |
| Difference |  |  |  |  |  |  |  |  |  |  |  |  |  |  |  |  |
| Stability coefficients |  |  |  |  |  |  |  |  |  |  |  |  |  |  |  |  |
| Support T1 🡪 Support T2 | 0.00 | [-0.22, | 0.22] | .998 | 0.00 | [-0.26, | 0.26] | .982 | -0.05 | [-0.28, | 0.17] | .652 | -0.05 | [-0.30, | 0.21] | .723 |
| Positive STV T1 🡪 Positive STV T2 | 0.09 | [-0.16, | 0.33] | .500 | 0.15 | [-0.09, | 0.39] | .227 | 0.21 | [-0.05, | 0.46] | .107 | 0.23 | [-0.01, | 0.46] | .056 |
| Positive STV T2 🡪 Positive STV T3 | -0.01 | [-0.35, | 0.34] | .970 | -0.10 | [-0.49, | 0.29] | .625 | -0.09 | [-0.33, | 0.15] | .483 | -0.12 | [-0.39, | 0.14] | .369 |
| Positive STV T1 🡪 Positive STV T3 | 0.05 | [-0.30, | 0.41] | .777 | -0.01 | [-0.39, | 0.38] | .981 | -0.14 | [-0.37, | 0.08] | .216 | -0.17 | [-0.39, | 0.06] | .151 |
| Cross-lagged coefficients |  |  |  |  |  |  |  |  |  |  |  |  |  |  |  |  |
| Support T1 🡪 Positive STV T2 | -0.25 | [-0.56, | 0.06] | .107 | -0.47 | [-0.76, | -0.19] | .001 | -0.14 | [-0.41, | 0.13] | .308 | -0.35 | [-0.59, | -0.11] | .004 |
| Support T2 🡪 Positive STV T3 | -0.43 | [-0.77, | -0.08] | .015 | 0.44 | [0.05, | 0.83] | .026 | -0.07 | [-0.42, | 0.28] | .696 | 0.09 | [-0.14, | 0.31] | .465 |
| Support T1 🡪 Positive STV T3 | 0.44 | [0.07, | 0.81] | .021 | -0.22 | [-0.59, | 0.15] | .238 | 0.19 | [-0.12, | 0.51] | .227 | 0.10 | [-0.16, | 0.36] | .442 |
| Positive STV T1 🡪 Support T2 | -0.03 | [-0.26, | 0.20] | .805 | -0.09 | [-0.36, | 0.18] | .527 | 0.09 | [-0.11, | 0.30] | .386 | -0.02 | [-0.26, | 0.22] | .869 |

**Table I6**

*Stability and Cross-Lagged Coefficients Path Models for Transfer and Non-Transfer Students and Cost in the Fall 2019 Data*

|  | Most Difficult Course | | | | | | | | Most Important Course | | | | | | | |
| --- | --- | --- | --- | --- | --- | --- | --- | --- | --- | --- | --- | --- | --- | --- | --- | --- |
|  | Peer support | | | | Faculty support | | | | Peer support | | | | Faculty support | | | |
|  | β | 95% CI | | *p* | β | 95% CI | | *p* | β | 95% CI | | *p* | β | 95% CI | | *p* |
| Non-transfer student |  |  |  |  |  |  |  |  |  |  |  |  |  |  |  |  |
| Stability coefficients |  |  |  |  |  |  |  |  |  |  |  |  |  |  |  |  |
| Support T1 🡪 Support T2 | 0.73 | [0.63, | 0.82] | <.001 | 0.64 | [0.54, | 0.75] | <.001 | 0.72 | [0.63, | 0.81] | <.001 | 0.64 | [0.54, | 0.75] | <.001 |
| Cost T1 🡪 Cost T2 | 0.44 | [0.30, | 0.58] | <.001 | 0.44 | [0.30, | 0.58] | <.001 | 0.60 | [0.47, | 0.72] | <.001 | 0.59 | [0.46, | 0.71] | <.001 |
| Cost T2 🡪 Cost T3 | 0.47 | [0.32, | 0.63] | <.001 | 0.46 | [0.30, | 0.62] | <.001 | 0.38 | [0.20, | 0.55] | <.001 | 0.37 | [0.20, | 0.54] | <.001 |
| Cost T1 🡪 Cost T3 | 0.26 | [0.11, | 0.42] | .001 | 0.26 | [0.10, | 0.42] | .001 | 0.26 | [0.12, | 0.40] | <.001 | 0.27 | [0.12, | 0.41] | <.001 |
| Cross-lagged coefficients |  |  |  |  |  |  |  |  |  |  |  |  |  |  |  |  |
| Support T1 🡪 Cost T2 | 0.00 | [-0.13, | 0.12] | .974 | -0.07 | [-0.20, | 0.06] | .301 | 0.00 | [-0.11, | 0.10] | .945 | -0.04 | [-0.16, | 0.08] | .531 |
| Support T2 🡪 Cost T3 | -0.05 | [-0.22, | 0.12] | .572 | 0.02 | [-0.13, | 0.17] | .769 | 0.03 | [-0.15, | 0.22] | .727 | 0.09 | [-0.10, | 0.28] | .353 |
| Support T1 🡪 Cost T3 | 0.24 | [0.06, | 0.42] | .008 | 0.11 | [-0.03, | 0.24] | .127 | 0.02 | [-0.15, | 0.20] | .804 | -0.01 | [-0.21, | 0.20] | .962 |
| Cost T1 🡪 Support T2 | 0.12 | [0.02, | 0.21] | .014 | -0.01 | [-0.13, | 0.10] | .805 | 0.09 | [0.00, | 0.18] | .044 | -0.05 | [-0.15, | 0.05] | .331 |
| Transfer students |  |  |  |  |  |  |  |  |  |  |  |  |  |  |  |  |
| Stability coefficients |  |  |  |  |  |  |  |  |  |  |  |  |  |  |  |  |
| Support T1 🡪 Support T2 | 0.74 | [0.54, | 0.95] | <.001 | 0.67 | [0.44, | 0.89] | <.001 | 0.74 | [0.54, | 0.95] | <.001 | 0.69 | [0.47, | 0.91] | <.001 |
| Cost T1 🡪 Cost T2 | 0.29 | [0.07, | 0.52] | .011 | 0.32 | [0.09, | 0.55] | .007 | 0.33 | [0.03, | 0.63] | .029 | 0.31 | [0.02, | 0.61] | .038 |
| Cost T2 🡪 Cost T3 | 0.28 | [0.02, | 0.54] | .039 | 0.21 | [-0.08, | 0.50] | .149 | 0.55 | [0.38, | 0.73] | <.001 | 0.58 | [0.39, | 0.76] | <.001 |
| Cost T1 🡪 Cost T3 | 0.25 | [0.02, | 0.49] | .033 | 0.27 | [0.02, | 0.52] | .032 | 0.52 | [0.34, | 0.70] | <.001 | 0.53 | [0.34, | 0.73] | <.001 |
| Cross-lagged coefficients |  |  |  |  |  |  |  |  |  |  |  |  |  |  |  |  |
| Support T1 🡪 Cost T2 | -0.10 | [-0.35, | 0.14] | .405 | 0.01 | [-0.26, | 0.29] | .920 | 0.06 | [-0.17, | 0.29] | .589 | 0.11 | [-0.16, | 0.38] | .413 |
| Support T2 🡪 Cost T3 | -0.16 | [-0.48, | 0.16] | .331 | 0.13 | [-0.21, | 0.47] | .451 | 0.25 | [-0.02, | 0.52] | .067 | 0.16 | [-0.09, | 0.40] | .216 |
| Support T1 🡪 Cost T3 | 0.14 | [-0.20, | 0.47] | .432 | -0.11 | [-0.38, | 0.17] | .452 | -0.12 | [-0.41, | 0.18] | .441 | -0.18 | [-0.35, | 0.00] | .046 |
| Cost T1 🡪 Support T2 | 0.01 | [-0.17, | 0.18] | .953 | -0.11 | [-0.35, | 0.14] | .390 | -0.05 | [-0.26, | 0.16] | .631 | -0.13 | [-0.33, | 0.07] | .199 |
| Difference |  |  |  |  |  |  |  |  |  |  |  |  |  |  |  |  |
| Stability coefficients |  |  |  |  |  |  |  |  |  |  |  |  |  |  |  |  |
| Support T1 🡪 Support T2 | -0.02 | [-0.24, | 0.20] | .864 | -0.02 | [-0.27, | 0.23] | .851 | -0.02 | [-0.25, | 0.21] | .851 | -0.05 | [-0.30, | 0.20] | .705 |
| Cost T1 🡪 Cost T2 | 0.15 | [-0.12, | 0.41] | .269 | 0.13 | [-0.14, | 0.39] | .362 | 0.27 | [-0.06, | 0.59] | .105 | 0.28 | [-0.05, | 0.60] | .092 |
| Cost T2 🡪 Cost T3 | 0.19 | [-0.11, | 0.50] | .215 | 0.25 | [-0.08, | 0.58] | .135 | -0.18 | [-0.42, | 0.07] | .153 | -0.20 | [-0.46, | 0.05] | .119 |
| Cost T1 🡪 Cost T3 | 0.01 | [-0.27, | 0.29] | .936 | -0.01 | [-0.31, | 0.28] | .936 | -0.26 | [-0.49, | -0.04] | .023 | -0.27 | [-0.51, | -0.03] | .028 |
| Cross-lagged coefficients |  |  |  |  |  |  |  |  |  |  |  |  |  |  |  |  |
| Support T1 🡪 Cost T2 | 0.10 | [-0.17, | 0.37] | .467 | -0.08 | [-0.39, | 0.22] | .593 | -0.07 | [-0.32, | 0.19] | .605 | -0.15 | [-0.44, | 0.14] | .317 |
| Support T2 🡪 Cost T3 | 0.11 | [-0.26, | 0.48] | .553 | -0.11 | [-0.48, | 0.26] | .570 | -0.22 | [-0.55, | 0.11] | .189 | -0.07 | [-0.38, | 0.25] | .682 |
| Support T1 🡪 Cost T3 | 0.10 | [-0.28, | 0.49] | .593 | 0.21 | [-0.10, | 0.52] | .177 | 0.14 | [-0.21, | 0.48] | .430 | 0.17 | [-0.09, | 0.44] | .203 |
| Cost T1 🡪 Support T2 | 0.11 | [-0.09, | 0.31] | .268 | 0.09 | [-0.18, | 0.36] | .497 | 0.15 | [-0.08, | 0.37] | .214 | 0.08 | [-0.15, | 0.30] | .488 |

**Table I7**

*Stability and Cross-Lagged Coefficients Path Models for FG and CG Students and Expectancy in the Fall 2020 Data*

|  | Most Difficult Course | | | | | | | | Most Important Course | | | | | | | |
| --- | --- | --- | --- | --- | --- | --- | --- | --- | --- | --- | --- | --- | --- | --- | --- | --- |
|  | Peer support | | | | Faculty support | | | | Peer support | | | | Faculty support | | | |
|  | β | 95% CI | | *p* | β | 95% CI | | *p* | β | 95% CI | | *p* | β | 95% CI | | *p* |
| CG student |  |  |  |  |  |  |  |  |  |  |  |  |  |  |  |  |
| Stability coefficients |  |  |  |  |  |  |  |  |  |  |  |  |  |  |  |  |
| Support T1 🡪 Support T2 | 0.68 | [0.57, | 0.80] | <.001 | 0.60 | [0.42, | 0.78] | <.001 | 0.71 | [0.59, | 0.82] | <.001 | 0.62 | [0.47, | 0.78] | <.001 |
| Expectancy T1 🡪 Expectancy T2 | 0.15 | [-0.03, | 0.34] | .095 | 0.10 | [-0.08, | 0.28] | .274 | 0.36 | [0.20, | 0.52] | <.001 | 0.35 | [0.19, | 0.51] | <.001 |
| Expectancy T2 🡪 Expectancy T3 | 0.38 | [0.22, | 0.55] | <.001 | 0.40 | [0.22, | 0.58] | <.001 | 0.59 | [0.41, | 0.78] | <.001 | 0.59 | [0.41, | 0.78] | <.001 |
| Expectancy T1 🡪 Expectancy T3 | 0.37 | [0.22, | 0.51] | <.001 | 0.35 | [0.21, | 0.50] | <.001 | 0.20 | [0.04, | 0.35] | .012 | 0.22 | [0.05, | 0.38] | .009 |
| Cross-lagged coefficients |  |  |  |  |  |  |  |  |  |  |  |  |  |  |  |  |
| Support T1 🡪 Expectancy T2 | 0.16 | [-0.02, | 0.35] | .086 | 0.25 | [0.07, | 0.43] | .007 | 0.03 | [-0.14, | 0.19] | .752 | 0.17 | [0.01, | 0.32] | .043 |
| Support T2 🡪 Expectancy T3 | -0.12 | [-0.37, | 0.14] | .368 | 0.04 | [-0.12, | 0.20] | .656 | 0.15 | [-0.13, | 0.42] | .289 | -0.14 | [-0.30, | 0.01] | .074 |
| Support T1 🡪 Expectancy T3 | 0.14 | [-0.14, | 0.42] | .320 | 0.00 | [-0.19, | 0.18] | .987 | -0.16 | [-0.41, | 0.10] | .227 | 0.03 | [-0.13, | 0.19] | .716 |
| Expectancy T1 🡪 Support T2 | 0.14 | [0.03, | 0.25] | .016 | 0.09 | [-0.08, | 0.26] | .289 | -0.01 | [-0.12, | 0.10] | .861 | 0.03 | [-0.11, | 0.18] | .649 |
| FG students |  |  |  |  |  |  |  |  |  |  |  |  |  |  |  |  |
| Stability coefficients |  |  |  |  |  |  |  |  |  |  |  |  |  |  |  |  |
| Support T1 🡪 Support T2 | 0.77 | [0.68, | 0.86] | <.001 | 0.65 | [0.54, | 0.76] | <.001 | 0.75 | [0.67, | 0.84] | <.001 | 0.65 | [0.55, | 0.75] | <.001 |
| Expectancy T1 🡪 Expectancy T2 | 0.41 | [0.27, | 0.56] | <.001 | 0.38 | [0.23, | 0.53] | <.001 | 0.40 | [0.29, | 0.52] | <.001 | 0.37 | [0.25, | 0.49] | <.001 |
| Expectancy T2 🡪 Expectancy T3 | 0.49 | [0.35, | 0.62] | <.001 | 0.48 | [0.34, | 0.62] | <.001 | 0.52 | [0.37, | 0.67] | <.001 | 0.52 | [0.36, | 0.68] | <.001 |
| Expectancy T1 🡪 Expectancy T3 | 0.31 | [0.16, | 0.46] | <.001 | 0.26 | [0.12, | 0.41] | .001 | 0.16 | [0.01, | 0.31] | .034 | 0.17 | [0.02, | 0.32] | .026 |
| Cross-lagged coefficients |  |  |  |  |  |  |  |  |  |  |  |  |  |  |  |  |
| Support T1 🡪 Expectancy T2 | -0.02 | [-0.18, | 0.14] | .821 | 0.08 | [-0.09, | 0.25] | .339 | -0.05 | [-0.20, | 0.10] | .492 | 0.09 | [-0.06, | 0.24] | .255 |
| Support T2 🡪 Expectancy T3 | 0.01 | [-0.16, | 0.17] | .955 | 0.06 | [-0.12, | 0.24] | .542 | 0.16 | [0.01, | 0.31] | .042 | 0.08 | [-0.13, | 0.30] | .460 |
| Support T1 🡪 Expectancy T3 | -0.02 | [-0.20, | 0.16] | .825 | 0.08 | [-0.09, | 0.25] | .361 | -0.11 | [-0.31, | 0.08] | .244 | -0.08 | [-0.29, | 0.13] | .454 |
| Expectancy T1 🡪 Support T2 | -0.06 | [-0.16, | 0.04] | .215 | 0.01 | [-0.11, | 0.13] | .864 | 0.00 | [-0.10, | 0.10] | .997 | 0.03 | [-0.09, | 0.14] | .654 |
| Difference |  |  |  |  |  |  |  |  |  |  |  |  |  |  |  |  |
| Stability coefficients |  |  |  |  |  |  |  |  |  |  |  |  |  |  |  |  |
| Support T1 🡪 Support T2 | -0.09 | [-0.23, | 0.05] | .222 | -0.06 | [-0.26, | 0.15] | .603 | -0.05 | [-0.19, | 0.10] | .515 | -0.03 | [-0.21, | 0.16] | .774 |
| Expectancy T1 🡪 Expectancy T2 | -0.26 | [-0.49, | -0.03] | .027 | -0.28 | [-0.52, | -0.04] | .020 | -0.04 | [-0.24, | 0.16] | .666 | -0.02 | [-0.22, | 0.18] | .810 |
| Expectancy T2 🡪 Expectancy T3 | -0.11 | [-0.32, | 0.11] | .331 | -0.08 | [-0.31, | 0.15] | .494 | 0.08 | [-0.16, | 0.31] | .538 | 0.07 | [-0.17, | 0.32] | .553 |
| Expectancy T1 🡪 Expectancy T3 | 0.06 | [-0.15, | 0.26] | .603 | 0.09 | [-0.12, | 0.30] | .400 | 0.04 | [-0.18, | 0.25] | .752 | 0.05 | [-0.17, | 0.27] | .669 |
| Cross-lagged coefficients |  |  |  |  |  |  |  |  |  |  |  |  |  |  |  |  |
| Support T1 🡪 Expectancy T2 | 0.18 | [-0.06, | 0.43] | .146 | 0.17 | [-0.08, | 0.41] | .186 | 0.08 | [-0.14, | 0.30] | .483 | 0.08 | [-0.14, | 0.30] | .495 |
| Support T2 🡪 Expectancy T3 | -0.12 | [-0.42, | 0.18] | .433 | -0.02 | [-0.26, | 0.22] | .872 | -0.01 | [-0.32, | 0.31] | .961 | -0.22 | [-0.49, | 0.04] | .099 |
| Support T1 🡪 Expectancy T3 | 0.16 | [-0.17, | 0.49] | .338 | -0.08 | [-0.33, | 0.17] | .526 | -0.04 | [-0.36, | 0.28] | .790 | 0.11 | [-0.15, | 0.37] | .414 |
| Expectancy T1 🡪 Support T2 | 0.20 | [0.05, | 0.34] | .008 | 0.08 | [-0.13, | 0.28] | .449 | -0.01 | [-0.16, | 0.14] | .899 | 0.01 | [-0.18, | 0.19] | .938 |

**Table I8**

*Stability and Cross-Lagged Coefficients Path Models for FG and CG Students and Positive STV in the Fall 2020 Data*

|  | Most Difficult Course | | | | | | | | Most Important Course | | | | | | | |
| --- | --- | --- | --- | --- | --- | --- | --- | --- | --- | --- | --- | --- | --- | --- | --- | --- |
|  | Peer support | | | | Faculty support | | | | Peer support | | | | Faculty support | | | |
|  | β | 95% CI | | *p* | β | 95% CI | | *p* | β | 95% CI | | *p* | β | 95% CI | | *p* |
| CG student |  |  |  |  |  |  |  |  |  |  |  |  |  |  |  |  |
| Stability coefficients |  |  |  |  |  |  |  |  |  |  |  |  |  |  |  |  |
| Support T1 🡪 Support T2 | 0.71 | [0.60, | 0.82] | <.001 | 0.61 | [0.44, | 0.77] | <.001 | 0.71 | [0.60, | 0.82] | <.001 | 0.62 | [0.46, | 0.78] | <.001 |
| Positive STV T1 🡪 Positive STV T2 | 0.61 | [0.49, | 0.73] | <.001 | 0.60 | [0.48, | 0.72] | <.001 | 0.61 | [0.46, | 0.76] | <.001 | 0.59 | [0.44, | 0.75] | <.001 |
| Positive STV T2 🡪 Positive STV T3 | 0.40 | [0.26, | 0.55] | <.001 | 0.40 | [0.26, | 0.54] | <.001 | 0.60 | [0.45, | 0.76] | <.001 | 0.63 | [0.47, | 0.78] | <.001 |
| Positive STV T1 🡪 Positive STV T3 | 0.44 | [0.28, | 0.61] | <.001 | 0.44 | [0.27, | 0.61] | <.001 | 0.32 | [0.14, | 0.50] | .001 | 0.29 | [0.12, | 0.46] | .001 |
| Cross-lagged coefficients |  |  |  |  |  |  |  |  |  |  |  |  |  |  |  |  |
| Support T1 🡪 Positive STV T2 | 0.03 | [-0.10, | 0.16] | .632 | 0.06 | [-0.07, | 0.19] | .356 | 0.10 | [-0.03, | 0.23] | .136 | 0.12 | [-0.01, | 0.25] | .077 |
| Support T2 🡪 Positive STV T3 | -0.01 | [-0.18, | 0.16] | .880 | 0.03 | [-0.14, | 0.20] | .739 | 0.18 | [0.01, | 0.35] | .042 | -0.15 | [-0.26, | -0.04] | .007 |
| Support T1 🡪 Positive STV T3 | -0.03 | [-0.20, | 0.15] | .756 | 0.00 | [-0.17, | 0.17] | .990 | -0.06 | [-0.25, | 0.12] | .501 | 0.14 | [0.04, | 0.25] | .009 |
| Positive STV T1 🡪 Support T2 | -0.01 | [-0.12, | 0.11] | .873 | 0.09 | [-0.05, | 0.23] | .186 | -0.05 | [-0.19, | 0.09] | .446 | 0.01 | [-0.14, | 0.16] | .911 |
| FG students |  |  |  |  |  |  |  |  |  |  |  |  |  |  |  |  |
| Stability coefficients |  |  |  |  |  |  |  |  |  |  |  |  |  |  |  |  |
| Support T1 🡪 Support T2 | 0.76 | [0.68, | 0.85] | <.001 | 0.66 | [0.55, | 0.76] | <.001 | 0.75 | [0.66, | 0.84] | <.001 | 0.67 | [0.57, | 0.78] | <.001 |
| Positive STV T1 🡪 Positive STV T2 | 0.62 | [0.51, | 0.74] | <.001 | 0.62 | [0.51, | 0.74] | <.001 | 0.60 | [0.51, | 0.70] | <.001 | 0.60 | [0.50, | 0.71] | <.001 |
| Positive STV T2 🡪 Positive STV T3 | 0.44 | [0.26, | 0.61] | <.001 | 0.43 | [0.25, | 0.61] | <.001 | 0.58 | [0.43, | 0.74] | <.001 | 0.57 | [0.42, | 0.73] | <.001 |
| Positive STV T1 🡪 Positive STV T3 | 0.33 | [0.17, | 0.48] | <.001 | 0.32 | [0.15, | 0.49] | <.001 | 0.21 | [0.08, | 0.34] | .002 | 0.23 | [0.09, | 0.37] | .001 |
| Cross-lagged coefficients |  |  |  |  |  |  |  |  |  |  |  |  |  |  |  |  |
| Support T1 🡪 Positive STV T2 | -0.05 | [-0.18, | 0.09] | .496 | -0.01 | [-0.13, | 0.11] | .835 | -0.01 | [-0.14, | 0.12] | .864 | 0.00 | [-0.12, | 0.13] | .979 |
| Support T2 🡪 Positive STV T3 | -0.10 | [-0.26, | 0.05] | .194 | -0.01 | [-0.18, | 0.17] | .955 | -0.14 | [-0.34, | 0.06] | .171 | 0.08 | [-0.08, | 0.25] | .330 |
| Support T1 🡪 Positive STV T3 | 0.09 | [-0.09, | 0.27] | .323 | 0.07 | [-0.11, | 0.25] | .416 | 0.18 | [-0.02, | 0.37] | .079 | -0.14 | [-0.30, | 0.03] | .104 |
| Positive STV T1 🡪 Support T2 | -0.05 | [-0.15, | 0.05] | .318 | 0.01 | [-0.09, | 0.12] | .824 | 0.06 | [-0.04, | 0.16] | .229 | -0.04 | [-0.14, | 0.06] | .433 |
| Difference |  |  |  |  |  |  |  |  |  |  |  |  |  |  |  |  |
| Stability coefficients |  |  |  |  |  |  |  |  |  |  |  |  |  |  |  |  |
| Support T1 🡪 Support T2 | -0.05 | [-0.19, | 0.09] | .463 | -0.05 | [-0.24, | 0.14] | .610 | -0.04 | [-0.18, | 0.10] | .593 | -0.05 | [-0.24, | 0.14] | .623 |
| Positive STV T1 🡪 Positive STV T2 | -0.02 | [-0.18, | 0.15] | .835 | -0.02 | [-0.19, | 0.15] | .788 | 0.01 | [-0.17, | 0.18] | .942 | -0.01 | [-0.20, | 0.17] | .904 |
| Positive STV T2 🡪 Positive STV T3 | -0.03 | [-0.26, | 0.20] | .782 | -0.03 | [-0.26, | 0.20] | .803 | 0.02 | [-0.20, | 0.24] | .844 | 0.05 | [-0.17, | 0.28] | .630 |
| Positive STV T1 🡪 Positive STV T3 | 0.12 | [-0.11, | 0.34] | .314 | 0.12 | [-0.12, | 0.36] | .311 | 0.11 | [-0.12, | 0.33] | .344 | 0.06 | [-0.15, | 0.28] | .578 |
| Cross-lagged coefficients |  |  |  |  |  |  |  |  |  |  |  |  |  |  |  |  |
| Support T1 🡪 Positive STV T2 | 0.08 | [-0.11, | 0.27] | .409 | 0.07 | [-0.10, | 0.25] | .413 | 0.11 | [-0.07, | 0.29] | .238 | 0.12 | [-0.07, | 0.30] | .207 |
| Support T2 🡪 Positive STV T3 | 0.09 | [-0.14, | 0.32] | .443 | 0.03 | [-0.21, | 0.28] | .783 | 0.32 | [0.06, | 0.58] | .018 | -0.23 | [-0.43, | -0.03] | .022 |
| Support T1 🡪 Positive STV T3 | -0.12 | [-0.37, | 0.13] | .357 | -0.08 | [-0.32, | 0.17] | .548 | -0.24 | [-0.51, | 0.03] | .083 | 0.28 | [0.08, | 0.47] | .005 |
| Positive STV T1 🡪 Support T2 | 0.04 | [-0.11, | 0.19] | .592 | 0.08 | [-0.09, | 0.26] | .356 | -0.11 | [-0.28, | 0.06] | .187 | 0.05 | [-0.13, | 0.23] | .606 |

**Table I9**

*Stability and Cross-Lagged Coefficients Path Models for FG and CG Students and, Positive STV in the Fall 2020 Data*

|  | Most Difficult Course | | | | | | | | Most Important Course | | | | | | | |
| --- | --- | --- | --- | --- | --- | --- | --- | --- | --- | --- | --- | --- | --- | --- | --- | --- |
|  | Peer support | | | | Faculty support | | | | Peer support | | | | Faculty support | | | |
|  | β | 95% CI | | *p* | β | 95% CI | | *p* | β | 95% CI | | *p* | β | 95% CI | | *p* |
| CG student |  |  |  |  |  |  |  |  |  |  |  |  |  |  |  |  |
| Stability coefficients |  |  |  |  |  |  |  |  |  |  |  |  |  |  |  |  |
| Support T1 🡪 Support T2 | 0.71 | [0.60, | 0.82] | <.001 | 0.61 | [0.45, | 0.76] | <.001 | 0.71 | [0.60, | 0.83] | <.001 | 0.63 | [0.47, | 0.78] | <.001 |
| Cost T1 🡪 Cost T2 | 0.38 | [0.22, | 0.53] | <.001 | 0.37 | [0.21, | 0.53] | <.001 | 0.34 | [0.18, | 0.51] | <.001 | 0.34 | [0.18, | 0.50] | <.001 |
| Cost T2 🡪 Cost T3 | 0.45 | [0.29, | 0.61] | <.001 | 0.45 | [0.28, | 0.62] | <.001 | 0.63 | [0.50, | 0.77] | <.001 | 0.64 | [0.50, | 0.78] | <.001 |
| Cost T1 🡪 Cost T3 | 0.21 | [0.04, | 0.37] | .015 | 0.22 | [0.04, | 0.39] | .015 | 0.20 | [0.05, | 0.35] | .009 | 0.17 | [0.01, | 0.32] | .033 |
| Cross-lagged coefficients |  |  |  |  |  |  |  |  |  |  |  |  |  |  |  |  |
| Support T1 🡪 Cost T2 | -0.03 | [-0.18, | 0.11] | .660 | -0.07 | [-0.23, | 0.09] | .364 | 0.04 | [-0.13, | 0.21] | .659 | -0.10 | [-0.27, | 0.07] | .228 |
| Support T2 🡪 Cost T3 | 0.02 | [-0.21, | 0.24] | .897 | -0.05 | [-0.24, | 0.15] | .642 | -0.25 | [-0.45, | -0.04] | .019 | -0.17 | [-0.32, | -0.02] | .025 |
| Support T1 🡪 Cost T3 | -0.16 | [-0.39, | 0.08] | .191 | 0.01 | [-0.21, | 0.24] | .903 | 0.20 | [0.01, | 0.39] | .036 | 0.11 | [-0.04, | 0.27] | .147 |
| Cost T1 🡪 Support T2 | 0.05 | [-0.07, | 0.17] | .411 | -0.08 | [-0.21, | 0.04] | .183 | 0.14 | [0.02, | 0.26] | .023 | 0.05 | [-0.08, | 0.18] | .428 |
| FG students |  |  |  |  |  |  |  |  |  |  |  |  |  |  |  |  |
| Stability coefficients |  |  |  |  |  |  |  |  |  |  |  |  |  |  |  |  |
| Support T1 🡪 Support T2 | 0.75 | [0.66, | 0.84] | <.001 | 0.67 | [0.58, | 0.77] | <.001 | 0.75 | [0.67, | 0.84] | <.001 | 0.66 | [0.56, | 0.76] | <.001 |
| Cost T1 🡪 Cost T2 | 0.38 | [0.25, | 0.52] | <.001 | 0.39 | [0.26, | 0.53] | <.001 | 0.30 | [0.15, | 0.44] | <.001 | 0.30 | [0.15, | 0.44] | <.001 |
| Cost T2 🡪 Cost T3 | 0.35 | [0.22, | 0.48] | <.001 | 0.33 | [0.19, | 0.47] | <.001 | 0.49 | [0.36, | 0.62] | <.001 | 0.50 | [0.36, | 0.64] | <.001 |
| Cost T1 🡪 Cost T3 | 0.44 | [0.30, | 0.59] | <.001 | 0.46 | [0.32, | 0.61] | <.001 | 0.26 | [0.11, | 0.41] | .001 | 0.26 | [0.10, | 0.41] | .001 |
| Cross-lagged coefficients |  |  |  |  |  |  |  |  |  |  |  |  |  |  |  |  |
| Support T1 🡪 Cost T2 | 0.08 | [-0.06, | 0.22] | .255 | 0.10 | [-0.05, | 0.25] | .186 | 0.00 | [-0.13, | 0.14] | .962 | 0.09 | [-0.05, | 0.23] | .204 |
| Support T2 🡪 Cost T3 | 0.12 | [-0.06, | 0.29] | .207 | -0.12 | [-0.28, | 0.05] | .171 | 0.02 | [-0.16, | 0.20] | .812 | 0.02 | [-0.17, | 0.21] | .825 |
| Support T1 🡪 Cost T3 | -0.06 | [-0.26, | 0.14] | .527 | 0.14 | [-0.04, | 0.31] | .135 | 0.17 | [0.00, | 0.35] | .050 | 0.04 | [-0.16, | 0.24] | .682 |
| Cost T1 🡪 Support T2 | -0.04 | [-0.15, | 0.07] | .503 | 0.07 | [-0.05, | 0.19] | .234 | -0.01 | [-0.10, | 0.09] | .874 | 0.03 | [-0.09, | 0.14] | .666 |
| Difference |  |  |  |  |  |  |  |  |  |  |  |  |  |  |  |  |
| Stability coefficients |  |  |  |  |  |  |  |  |  |  |  |  |  |  |  |  |
| Support T1 🡪 Support T2 | -0.04 | [-0.18, | 0.10] | .569 | -0.07 | [-0.25, | 0.12] | .486 | -0.04 | [-0.19, | 0.10] | .567 | -0.03 | [-0.22, | 0.15] | .720 |
| Cost T1 🡪 Cost T2 | -0.01 | [-0.21, | 0.20] | .945 | -0.02 | [-0.23, | 0.18] | .825 | 0.05 | [-0.17, | 0.27] | .667 | 0.04 | [-0.18, | 0.26] | .717 |
| Cost T2 🡪 Cost T3 | 0.10 | [-0.11, | 0.31] | .358 | 0.12 | [-0.10, | 0.34] | .268 | 0.14 | [-0.05, | 0.33] | .136 | 0.14 | [-0.06, | 0.34] | .167 |
| Cost T1 🡪 Cost T3 | -0.24 | [-0.46, | -0.02] | .035 | -0.25 | [-0.47, | -0.02] | .034 | -0.06 | [-0.27, | 0.15] | .598 | -0.09 | [-0.31, | 0.13] | .413 |
| Cross-lagged coefficients |  |  |  |  |  |  |  |  |  |  |  |  |  |  |  |  |
| Support T1 🡪 Cost T2 | -0.11 | [-0.31, | 0.09] | .268 | -0.17 | [-0.39, | 0.04] | .117 | 0.04 | [-0.18, | 0.25] | .752 | -0.20 | [-0.42, | 0.03] | .082 |
| Support T2 🡪 Cost T3 | -0.10 | [-0.38, | 0.18] | .488 | 0.07 | [-0.18, | 0.32] | .589 | -0.27 | [-0.54, | 0.00] | .054 | -0.19 | [-0.43, | 0.05] | .123 |
| Support T1 🡪 Cost T3 | -0.09 | [-0.40, | 0.22] | .557 | -0.12 | [-0.41, | 0.16] | .398 | 0.03 | [-0.23, | 0.28] | .832 | 0.07 | [-0.18, | 0.32] | .573 |
| Cost T1 🡪 Support T2 | 0.09 | [-0.07, | 0.25] | .290 | -0.15 | [-0.32, | 0.02] | .074 | 0.15 | [-0.01, | 0.30] | .061 | 0.03 | [-0.14, | 0.19] | .760 |

**Table I10**

*Stability and Cross-Lagged Coefficients Path Models for Transfer and Non-Transfer Students and Expectancy in the Fall 2020 Data*

|  | Most Difficult Course | | | | | | | | Most Important Course | | | | | | | |
| --- | --- | --- | --- | --- | --- | --- | --- | --- | --- | --- | --- | --- | --- | --- | --- | --- |
|  | Peer support | | | | Faculty support | | | | Peer support | | | | Faculty support | | | |
|  | β | 95% CI | | *p* | β | 95% CI | | *p* | β | 95% CI | | *p* | β | 95% CI | | *p* |
| Non-transfer student |  |  |  |  |  |  |  |  |  |  |  |  |  |  |  |  |
| Stability coefficients |  |  |  |  |  |  |  |  |  |  |  |  |  |  |  |  |
| Support T1 🡪 Support T2 | 0.70 | [0.61, | 0.78] | <.001 | 0.68 | [0.59, | 0.77] | <.001 | 0.70 | [0.62, | 0.78] | <.001 | 0.68 | [0.60, | 0.76] | <.001 |
| Expectancy T1 🡪 Expectancy T2 | 0.32 | [0.20, | 0.43] | <.001 | 0.29 | [0.16, | 0.41] | <.001 | 0.38 | [0.28, | 0.49] | <.001 | 0.35 | [0.24, | 0.45] | <.001 |
| Expectancy T2 🡪 Expectancy T3 | 0.42 | [0.29, | 0.54] | <.001 | 0.41 | [0.28, | 0.54] | <.001 | 0.54 | [0.40, | 0.67] | <.001 | 0.54 | [0.40, | 0.69] | <.001 |
| Expectancy T1 🡪 Expectancy T3 | 0.37 | [0.24, | 0.49] | <.001 | 0.33 | [0.20, | 0.46] | <.001 | 0.22 | [0.10, | 0.34] | <.001 | 0.22 | [0.09, | 0.34] | .001 |
| Cross-lagged coefficients |  |  |  |  |  |  |  |  |  |  |  |  |  |  |  |  |
| Support T1 🡪 Expectancy T2 | 0.06 | [-0.07, | 0.19] | .359 | 0.11 | [-0.02, | 0.25] | .097 | 0.02 | [-0.11, | 0.14] | .813 | 0.17 | [0.06, | 0.28] | .004 |
| Support T2 🡪 Expectancy T3 | -0.12 | [-0.28, | 0.04] | .132 | 0.01 | [-0.16, | 0.17] | .947 | 0.17 | [0.00, | 0.33] | .050 | -0.04 | [-0.22, | 0.14] | .666 |
| Support T1 🡪 Expectancy T3 | 0.10 | [-0.07, | 0.27] | .244 | 0.10 | [-0.06, | 0.27] | .203 | -0.17 | [-0.34, | 0.01] | .061 | 0.00 | [-0.18, | 0.18] | .971 |
| Expectancy T1 🡪 Support T2 | 0.01 | [-0.08, | 0.10] | .786 | 0.04 | [-0.06, | 0.14] | .397 | 0.03 | [-0.07, | 0.12] | .586 | 0.06 | [-0.04, | 0.15] | .226 |
| Transfer students |  |  |  |  |  |  |  |  |  |  |  |  |  |  |  |  |
| Stability coefficients |  |  |  |  |  |  |  |  |  |  |  |  |  |  |  |  |
| Support T1 🡪 Support T2 | 0.74 | [0.61, | 0.88] | <.001 | 0.50 | [0.24, | 0.75] | <.001 | 0.75 | [0.62, | 0.88] | <.001 | 0.51 | [0.29, | 0.73] | <.001 |
| Expectancy T1 🡪 Expectancy T2 | 0.16 | [-0.13, | 0.45] | .267 | 0.12 | [-0.17, | 0.41] | .416 | 0.44 | [0.25, | 0.63] | <.001 | 0.43 | [0.23, | 0.63] | <.001 |
| Expectancy T2 🡪 Expectancy T3 | 0.45 | [0.26, | 0.65] | <.001 | 0.48 | [0.28, | 0.68] | <.001 | 0.59 | [0.41, | 0.76] | <.001 | 0.59 | [0.43, | 0.75] | <.001 |
| Expectancy T1 🡪 Expectancy T3 | 0.34 | [0.17, | 0.52] | <.001 | 0.34 | [0.17, | 0.51] | <.001 | 0.08 | [-0.14, | 0.29] | .492 | 0.08 | [-0.13, | 0.30] | .449 |
| Cross-lagged coefficients |  |  |  |  |  |  |  |  |  |  |  |  |  |  |  |  |
| Support T1 🡪 Expectancy T2 | 0.10 | [-0.15, | 0.35] | .418 | 0.29 | [0.03, | 0.56] | .031 | -0.03 | [-0.22, | 0.17] | .800 | 0.02 | [-0.20, | 0.24] | .835 |
| Support T2 🡪 Expectancy T3 | 0.29 | [0.01, | 0.57] | .043 | 0.15 | [-0.08, | 0.38] | .213 | 0.02 | [-0.22, | 0.26] | .876 | -0.08 | [-0.23, | 0.07] | .282 |
| Support T1 🡪 Expectancy T3 | -0.15 | [-0.46, | 0.16] | .352 | -0.07 | [-0.30, | 0.15] | .510 | 0.04 | [-0.21, | 0.29] | .757 | 0.02 | [-0.13, | 0.16] | .843 |
| Expectancy T1 🡪 Support T2 | -0.01 | [-0.13, | 0.10] | .839 | 0.02 | [-0.19, | 0.24] | .823 | -0.15 | [-0.27, | -0.04] | .010 | -0.05 | [-0.22, | 0.12] | .584 |
| Difference |  |  |  |  |  |  |  |  |  |  |  |  |  |  |  |  |
| Stability coefficients |  |  |  |  |  |  |  |  |  |  |  |  |  |  |  |  |
| Support T1 🡪 Support T2 | -0.05 | [-0.21, | 0.11] | .551 | 0.18 | [-0.09, | 0.45] | .184 | -0.05 | [-0.21, | 0.10] | .524 | 0.17 | [-0.06, | 0.40] | .150 |
| Expectancy T1 🡪 Expectancy T2 | 0.16 | [-0.16, | 0.46] | .328 | 0.17 | [-0.15, | 0.49] | .304 | -0.06 | [-0.28, | 0.16] | .599 | -0.08 | [-0.31, | 0.15] | .483 |
| Expectancy T2 🡪 Expectancy T3 | -0.03 | [-0.27, | 0.20] | .771 | -0.07 | [-0.31, | 0.17] | .567 | -0.05 | [-0.28, | 0.17] | .649 | -0.05 | [-0.26, | 0.17] | .660 |
| Expectancy T1 🡪 Expectancy T3 | 0.02 | [-0.19, | 0.24] | .840 | -0.01 | [-0.22, | 0.20] | .919 | 0.14 | [-0.11, | 0.39] | .263 | 0.13 | [-0.12, | 0.38] | .304 |
| Cross-lagged coefficients |  |  |  |  |  |  |  |  |  |  |  |  |  |  |  |  |
| Support T1 🡪 Expectancy T2 | -0.04 | [-0.32, | 0.24] | .770 | -0.18 | [-0.48, | 0.12] | .240 | 0.04 | [-0.19, | 0.27] | .734 | 0.15 | [-0.10, | 0.39] | .244 |
| Support T2 🡪 Expectancy T3 | -0.41 | [-0.74, | -0.09] | .012 | -0.14 | [-0.42, | 0.14] | .331 | 0.15 | [-0.15, | 0.44] | .325 | 0.04 | [-0.19, | 0.28] | .730 |
| Support T1 🡪 Expectancy T3 | 0.25 | [-0.11, | 0.60] | .170 | 0.18 | [-0.09, | 0.45] | .200 | -0.21 | [-0.51, | 0.10] | .181 | -0.01 | [-0.24, | 0.22] | .921 |
| Expectancy T1 🡪 Support T2 | 0.02 | [-0.12, | 0.17] | .744 | 0.02 | [-0.22, | 0.25] | .884 | 0.18 | [0.03, | 0.33] | .019 | 0.11 | [-0.09, | 0.30] | .286 |

**Table I11**

*Stability and Cross-Lagged Coefficients Path Models for Transfer and Non-Transfer Students and Positive STV in the Fall 2020 Data*

|  | Most Difficult Course | | | | | | | | Most Important Course | | | | | | | |
| --- | --- | --- | --- | --- | --- | --- | --- | --- | --- | --- | --- | --- | --- | --- | --- | --- |
|  | Peer support | | | | Faculty support | | | | Peer support | | | | Faculty support | | | |
|  | β | 95% CI | | *p* | β | 95% CI | | *p* | β | 95% CI | | *p* | β | 95% CI | | *p* |
| Non-transfer student |  |  |  |  |  |  |  |  |  |  |  |  |  |  |  |  |
| Stability coefficients |  |  |  |  |  |  |  |  |  |  |  |  |  |  |  |  |
| Support T1 🡪 Support T2 | 0.71 | [0.63, | 0.78] | <.001 | 0.69 | [0.61, | 0.77] | <.001 | 0.70 | [0.62, | 0.78] | <.001 | 0.70 | [0.62, | 0.79] | <.001 |
| Positive STV T1 🡪 Positive STV T2 | 0.61 | [0.52, | 0.70] | <.001 | 0.61 | [0.52, | 0.70] | <.001 | 0.60 | [0.51, | 0.70] | <.001 | 0.58 | [0.48, | 0.68] | <.001 |
| Positive STV T2 🡪 Positive STV T3 | 0.37 | [0.24, | 0.51] | <.001 | 0.36 | [0.23, | 0.50] | <.001 | 0.61 | [0.48, | 0.74] | <.001 | 0.61 | [0.48, | 0.74] | <.001 |
| Positive STV T1 🡪 Positive STV T3 | 0.41 | [0.29, | 0.54] | <.001 | 0.41 | [0.28, | 0.54] | <.001 | 0.26 | [0.14, | 0.39] | <.001 | 0.27 | [0.14, | 0.40] | <.001 |
| Cross-lagged coefficients |  |  |  |  |  |  |  |  |  |  |  |  |  |  |  |  |
| Support T1 🡪 Positive STV T2 | 0.01 | [-0.10, | 0.11] | .924 | 0.01 | [-0.08, | 0.10] | .789 | 0.04 | [-0.06, | 0.14] | .432 | 0.10 | [0.00, | 0.20] | .041 |
| Support T2 🡪 Positive STV T3 | -0.07 | [-0.20, | 0.06] | .310 | 0.09 | [-0.07, | 0.25] | .283 | 0.02 | [-0.12, | 0.16] | .760 | 0.04 | [-0.08, | 0.17] | .502 |
| Support T1 🡪 Positive STV T3 | 0.09 | [-0.06, | 0.23] | .229 | 0.01 | [-0.16, | 0.18] | .916 | -0.02 | [-0.17, | 0.12] | .767 | -0.05 | [-0.17, | 0.07] | .434 |
| Positive STV T1 🡪 Support T2 | -0.04 | [-0.12, | 0.05] | .370 | 0.02 | [-0.07, | 0.10] | .695 | 0.04 | [-0.05, | 0.14] | .383 | -0.03 | [-0.11, | 0.06] | .572 |
| Transfer students |  |  |  |  |  |  |  |  |  |  |  |  |  |  |  |  |
| Stability coefficients |  |  |  |  |  |  |  |  |  |  |  |  |  |  |  |  |
| Support T1 🡪 Support T2 | 0.75 | [0.61, | 0.88] | <.001 | 0.48 | [0.26, | 0.70] | <.001 | 0.75 | [0.61, | 0.88] | <.001 | 0.51 | [0.29, | 0.72] | <.001 |
| Positive STV T1 🡪 Positive STV T2 | 0.65 | [0.49, | 0.81] | <.001 | 0.64 | [0.48, | 0.79] | <.001 | 0.61 | [0.44, | 0.77] | <.001 | 0.61 | [0.44, | 0.79] | <.001 |
| Positive STV T2 🡪 Positive STV T3 | 0.62 | [0.45, | 0.78] | <.001 | 0.60 | [0.43, | 0.77] | <.001 | 0.51 | [0.28, | 0.74] | <.001 | 0.46 | [0.23, | 0.68] | <.001 |
| Positive STV T1 🡪 Positive STV T3 | 0.28 | [0.09, | 0.47] | .003 | 0.29 | [0.10, | 0.48] | .003 | 0.17 | [-0.04, | 0.37] | .111 | 0.21 | [0.01, | 0.41] | .040 |
| Cross-lagged coefficients |  |  |  |  |  |  |  |  |  |  |  |  |  |  |  |  |
| Support T1 🡪 Positive STV T2 | -0.05 | [-0.24, | 0.14] | .601 | 0.07 | [-0.14, | 0.27] | .536 | -0.01 | [-0.17, | 0.15] | .922 | -0.03 | [-0.21, | 0.14] | .718 |
| Support T2 🡪 Positive STV T3 | -0.12 | [-0.35, | 0.11] | .302 | -0.09 | [-0.23, | 0.06] | .258 | 0.06 | [-0.32, | 0.45] | .742 | -0.13 | [-0.30, | 0.04] | .123 |
| Support T1 🡪 Positive STV T3 | 0.04 | [-0.21, | 0.28] | .769 | 0.10 | [-0.05, | 0.24] | .182 | 0.06 | [-0.30, | 0.42] | .750 | 0.02 | [-0.14, | 0.19] | .783 |
| Positive STV T1 🡪 Support T2 | -0.06 | [-0.22, | 0.10] | .472 | 0.17 | [-0.02, | 0.35] | .076 | -0.15 | [-0.30, | 0.00] | .051 | 0.00 | [-0.18, | 0.19] | .984 |
| Difference |  |  |  |  |  |  |  |  |  |  |  |  |  |  |  |  |
| Stability coefficients |  |  |  |  |  |  |  |  |  |  |  |  |  |  |  |  |
| Support T1 🡪 Support T2 | -0.04 | [-0.20, | 0.12] | .627 | 0.21 | [-0.02, | 0.45] | .075 | -0.05 | [-0.20, | 0.11] | .546 | 0.20 | [-0.03, | 0.43] | .092 |
| Positive STV T1 🡪 Positive STV T2 | -0.04 | [-0.22, | 0.14] | .688 | -0.03 | [-0.21, | 0.15] | .769 | 0.00 | [-0.19, | 0.19] | .979 | -0.03 | [-0.23, | 0.17] | .739 |
| Positive STV T2 🡪 Positive STV T3 | -0.25 | [-0.46, | -0.03] | .025 | -0.24 | [-0.46, | -0.02] | .031 | 0.10 | [-0.16, | 0.37] | .451 | 0.16 | [-0.11, | 0.42] | .244 |
| Positive STV T1 🡪 Positive STV T3 | 0.13 | [-0.10, | 0.36] | .258 | 0.12 | [-0.11, | 0.36] | .313 | 0.10 | [-0.14, | 0.34] | .417 | 0.06 | [-0.17, | 0.30] | .598 |
| Cross-lagged coefficients |  |  |  |  |  |  |  |  |  |  |  |  |  |  |  |  |
| Support T1 🡪 Positive STV T2 | 0.06 | [-0.16, | 0.27] | .614 | -0.05 | [-0.28, | 0.17] | .647 | 0.05 | [-0.14, | 0.24] | .613 | 0.13 | [-0.07, | 0.33] | .187 |
| Support T2 🡪 Positive STV T3 | 0.05 | [-0.22, | 0.32] | .699 | 0.17 | [-0.05, | 0.39] | .119 | -0.04 | [-0.45, | 0.36] | .837 | 0.18 | [-0.04, | 0.39] | .102 |
| Support T1 🡪 Positive STV T3 | 0.05 | [-0.23, | 0.33] | .713 | -0.09 | [-0.31, | 0.13] | .432 | -0.08 | [-0.47, | 0.31] | .685 | -0.07 | [-0.28, | 0.13] | .490 |
| Positive STV T1 🡪 Support T2 | 0.02 | [-0.16, | 0.20] | .823 | -0.15 | [-0.36, | 0.05] | .148 | 0.19 | [0.01, | 0.37] | .034 | -0.03 | [-0.23, | 0.18] | .795 |

**Table I12**

*Stability and Cross-Lagged Coefficients Path Models for Transfer and Non-Transfer Students and Cost in the Fall 2020 Data*

|  | Most Difficult Course | | | | | | | | Most Important Course | | | | | | | |
| --- | --- | --- | --- | --- | --- | --- | --- | --- | --- | --- | --- | --- | --- | --- | --- | --- |
|  | Peer support | | | | Faculty support | | | | Peer support | | | | Faculty support | | | |
|  | β | 95% CI | | *p* | β | 95% CI | | *p* | β | 95% CI | | *p* | β | 95% CI | | *p* |
| Non-transfer student |  |  |  |  |  |  |  |  |  |  |  |  |  |  |  |  |
| Stability coefficients |  |  |  |  |  |  |  |  |  |  |  |  |  |  |  |  |
| Support T1 🡪 Support T2 | 0.70 | [0.62, | 0.78] | <.001 | 0.70 | [0.62, | 0.77] | <.001 | 0.70 | [0.62, | 0.78] | <.001 | 0.70 | [0.62, | 0.77] | <.001 |
| Cost T1 🡪 Cost T2 | 0.37 | [0.26, | 0.49] | <.001 | 0.38 | [0.26, | 0.49] | <.001 | 0.38 | [0.26, | 0.50] | <.001 | 0.38 | [0.26, | 0.50] | <.001 |
| Cost T2 🡪 Cost T3 | 0.44 | [0.33, | 0.55] | <.001 | 0.44 | [0.32, | 0.55] | <.001 | 0.60 | [0.50, | 0.70] | <.001 | 0.60 | [0.49, | 0.70] | <.001 |
| Cost T1 🡪 Cost T3 | 0.27 | [0.15, | 0.39] | <.001 | 0.26 | [0.13, | 0.39] | <.001 | 0.23 | [0.11, | 0.35] | <.001 | 0.22 | [0.10, | 0.34] | <.001 |
| Cross-lagged coefficients |  |  |  |  |  |  |  |  |  |  |  |  |  |  |  |  |
| Support T1 🡪 Cost T2 | 0.01 | [-0.10, | 0.13] | .838 | 0.02 | [-0.11, | 0.14] | .790 | -0.03 | [-0.14, | 0.09] | .669 | -0.04 | [-0.16, | 0.07] | .447 |
| Support T2 🡪 Cost T3 | 0.13 | [-0.02, | 0.28] | .093 | -0.08 | [-0.25, | 0.08] | .324 | -0.02 | [-0.16, | 0.12] | .772 | 0.01 | [-0.15, | 0.16] | .941 |
| Support T1 🡪 Cost T3 | -0.18 | [-0.34, | -0.02] | .028 | -0.02 | [-0.18, | 0.14] | .811 | 0.14 | [0.00, | 0.28] | .044 | -0.04 | [-0.19, | 0.12] | .645 |
| Cost T1 🡪 Support T2 | 0.01 | [-0.08, | 0.11] | .795 | 0.00 | [-0.09, | 0.09] | .986 | 0.05 | [-0.04, | 0.14] | .260 | 0.01 | [-0.07, | 0.10] | .762 |
| Transfer students |  |  |  |  |  |  |  |  |  |  |  |  |  |  |  |  |
| Stability coefficients |  |  |  |  |  |  |  |  |  |  |  |  |  |  |  |  |
| Support T1 🡪 Support T2 | 0.74 | [0.59, | 0.88] | <.001 | 0.50 | [0.28, | 0.72] | <.001 | 0.74 | [0.59, | 0.88] | <.001 | 0.50 | [0.28, | 0.72] | <.001 |
| Cost T1 🡪 Cost T2 | 0.31 | [0.10, | 0.52] | .004 | 0.31 | [0.10, | 0.52] | .004 | 0.13 | [-0.11, | 0.37] | .304 | 0.14 | [-0.10, | 0.37] | .265 |
| Cost T2 🡪 Cost T3 | 0.34 | [0.09, | 0.59] | .008 | 0.34 | [0.08, | 0.61] | .010 | 0.44 | [0.23, | 0.65] | <.001 | 0.39 | [0.14, | 0.64] | .002 |
| Cost T1 🡪 Cost T3 | 0.42 | [0.19, | 0.64] | <.001 | 0.45 | [0.21, | 0.69] | <.001 | 0.30 | [0.12, | 0.49] | .001 | 0.28 | [0.08, | 0.47] | .006 |
| Cross-lagged coefficients |  |  |  |  |  |  |  |  |  |  |  |  |  |  |  |  |
| Support T1 🡪 Cost T2 | -0.08 | [-0.29, | 0.14] | .485 | -0.07 | [-0.28, | 0.14] | .513 | 0.01 | [-0.21, | 0.24] | .904 | 0.06 | [-0.17, | 0.30] | .609 |
| Support T2 🡪 Cost T3 | -0.18 | [-0.54, | 0.17] | .319 | 0.00 | [-0.21, | 0.21] | .977 | -0.39 | [-0.69, | -0.08] | .013 | -0.16 | [-0.38, | 0.06] | .154 |
| Support T1 🡪 Cost T3 | 0.11 | [-0.23, | 0.45] | .521 | 0.12 | [-0.13, | 0.36] | .348 | 0.30 | [0.05, | 0.56] | .021 | 0.22 | [0.05, | 0.38] | .010 |
| Cost T1 🡪 Support T2 | -0.06 | [-0.20, | 0.07] | .345 | -0.03 | [-0.24, | 0.17] | .740 | 0.05 | [-0.08, | 0.17] | .455 | -0.03 | [-0.22, | 0.17] | .803 |
| Difference |  |  |  |  |  |  |  |  |  |  |  |  |  |  |  |  |
| Stability coefficients |  |  |  |  |  |  |  |  |  |  |  |  |  |  |  |  |
| Support T1 🡪 Support T2 | -0.04 | [-0.20, | 0.13] | .660 | 0.20 | [-0.03, | 0.43] | .091 | -0.04 | [-0.20, | 0.13] | .658 | 0.20 | [-0.04, | 0.43] | .100 |
| Cost T1 🡪 Cost T2 | 0.06 | [-0.18, | 0.30] | .612 | 0.06 | [-0.18, | 0.31] | .609 | 0.26 | [-0.01, | 0.52] | .061 | 0.24 | [-0.02, | 0.51] | .074 |
| Cost T2 🡪 Cost T3 | 0.10 | [-0.18, | 0.38] | .481 | 0.09 | [-0.19, | 0.38] | .527 | 0.16 | [-0.07, | 0.39] | .177 | 0.21 | [-0.06, | 0.47] | .135 |
| Cost T1 🡪 Cost T3 | -0.15 | [-0.40, | 0.11] | .262 | -0.20 | [-0.47, | 0.08] | .159 | -0.08 | [-0.30, | 0.14] | .499 | -0.06 | [-0.29, | 0.18] | .642 |
| Cross-lagged coefficients |  |  |  |  |  |  |  |  |  |  |  |  |  |  |  |  |
| Support T1 🡪 Cost T2 | 0.09 | [-0.15, | 0.33] | .477 | 0.09 | [-0.16, | 0.33] | .484 | -0.04 | [-0.30, | 0.22] | .763 | -0.11 | [-0.37, | 0.16] | .428 |
| Support T2 🡪 Cost T3 | 0.31 | [-0.08, | 0.69] | .117 | -0.09 | [-0.36, | 0.18] | .525 | 0.37 | [0.03, | 0.70] | .032 | 0.16 | [-0.10, | 0.43] | .227 |
| Support T1 🡪 Cost T3 | -0.29 | [-0.66, | 0.08] | .127 | -0.14 | [-0.43, | 0.16] | .361 | -0.16 | [-0.46, | 0.13] | .272 | -0.26 | [-0.48, | -0.03] | .029 |
| Cost T1 🡪 Support T2 | 0.08 | [-0.09, | 0.24] | .357 | 0.04 | [-0.19, | 0.26] | .755 | 0.01 | [-0.15, | 0.16] | .948 | 0.04 | [-0.18, | 0.26] | .726 |

**References**

Benden, D. K., & Lauermann, F. (2022). Students’ motivational trajectories and academic success in math-intensive study programs: Why short-term motivational assessments matter. *Journal of Educational Psychology*, *114*(5), 1062–1085. https://doi.org/10.1037/edu0000708

Beymer, P. N., Benden, D. K., & Sachisthal, M. S. M. (2022). Exploring the dynamics of situated expectancy-value theory: A panel network analysis. *Learning and Individual Differences*, *100*, Article 102233.

Beymer, P. N., Ferland, M., & Flake, J. K. (2022). Validity evidence for a short scale of college students’ perceptions of cost. *Current Psychology*, *41*(11), 7937–7956. https://doi.org/10.1007/s12144-020-01218-w

Chen, F. F. (2007). Sensitivity of goodness of fit indexes to lack of measurement invariance. *Structural Equation Modeling*, *14*(3), 464–504. https://doi.org/10.1080/10705510701301834

Cheung, G. W., & Rensvold, R. B. (2002). Evaluating goodness-of-fit indexes for testing measurement invariance. *Structural Equation Modeling*, *9*(2), 233–255. https://doi.org/10.1207/S15328007SEM0902_5

Eccles, J. S., & Wigfield, A. (2020). From expectancy-value theory to situated expectancy-value theory: A developmental, social cognitive, and sociocultural perspective on motivation. *Contemporary Educational Psychology*, *61*, Article 101859. https://doi.org/10.1016/j.cedpsych.2020.101859

Flake, J. K., Barron, K. E., Hulleman, C., McCoach, B. D., & Welsh, M. E. (2015). Measuring cost: The forgotten component of expectancy-value theory. *Contemporary Educational Psychology*, *41*, 232–244. https://doi.org/10.1016/j.cedpsych.2015.03.002

Gehlbach, H. (2015). Seven survey sins. *Journal of Early Adolescence*, *35*(5–6), 883–897. https://doi.org/10.1177/0272431615578276

Gogol, K., Brunner, M., Goetz, T., Martin, R., Ugen, S., Keller, U., Fischbach, A., & Preckel, F. (2014). “My questionnaire is too long!” The assessments of motivational-affective constructs with three-item and single-item measures. *Contemporary Educational Psychology*, *39*(3), 188–205. https://doi.org/10.1016/j.cedpsych.2014.04.002

Hoffman, M., Richmond, J., Morrow, J., & Salomone, K. (2002). Investigating “sense of belonging” in first-year college students. *Journal of College Student Retention*, *4*(3), 227–256. https://doi.org/10.2190/dryc-cxq9-jq8v-ht4v

Hu, L., & Bentler, P. M. (1999). Cutoff criteria for fit indexes in covariance structure analysis: Conventional criteria versus new alternatives. *Structural Equation Modeling*, *6*(1), 1–55. https://doi.org/10.1080/10705519909540118

Marsh, H. W., Hau, K., & Wen, Z. (2004). In search of golden rules: Comment on hypothesis-testing approaches to setting cutoff values for fit indexes and dangers in overgeneralizing Hu and Bentler’ s (1999) findings. *Structural Equation Modeling*, *11*, 37–41. https://doi.org/10.1207/s15328007sem1103

Perez, T., Cromley, J. G., & Kaplan, A. (2014). The role of identity development, values, and costs in college STEM retention. *Journal of Educational Psychology*, *106*(1), 315–329. https://doi.org/10.1037/a0034027

Saris, W. E., Revilla, M., Krosnick, J. A., & Shaeffer, E. M. (2010). Comparing questions with agree/disagree response options to questions with item-specific response options. *Survey Research Methods*, *4*(1), 61–79. https://doi.org/10.18148/srm/2010.v4i1.2682

Wentzel, K. R., Battle, A., Russell, S. L., & Looney, L. B. (2010). Social supports from teachers and peers as predictors of academic and social motivation. *Contemporary Educational Psychology*, *35*(3), 193–202. https://doi.org/10.1016/j.cedpsych.2010.03.002
